# Diagnostic value of biomarkers for paediatric urinary tract infections in primary care: systematic review and meta-analysis

**DOI:** 10.1186/s12875-021-01530-9

**Published:** 2021-09-27

**Authors:** Hanne A. Boon, Thomas Struyf, Dominique Bullens, Ann Van den Bruel, Jan Y. Verbakel

**Affiliations:** 1 EPI-Centre, Academic Centre for General Practice, KU Leuven, Kapucijnenvoer 7, 3000 Leuven, Belgium; 2 Department of Microbiology, Immunology and Transplantation, KU Leuven, Herestraat 49, Box 811, 3000 Leuven, Belgium; 3 Clinical Division of Pediatrics, UZ Leuven, Herestraat 49, 3000 Leuven, Belgium; 4 Nuffield Department of Primary Care Health Sciences, University of Oxford, Radcliffe Primary Care Building, Radcliffe Observatory Quarter, Woodstock Road, Oxford, OX2 6GG UK

**Keywords:** Urinary tract infections, Child, Primary health care, Diagnosis, Biomarkers, Meta-analysis

## Abstract

**Background:**

Accurate diagnosis of urinary tract infection is essential as children left untreated may suffer permanent renal injury.

**Aim:**

To compare the diagnostic values of biomarkers or clinical prediction rules for urinary tract infections in children presenting to ambulatory care.

**Design and setting:**

Systematic review and meta-analysis of ambulatory care studies.

**Methods:**

Medline, Embase, WOS, CINAHL, Cochrane library, HTA and DARE were searched until 21 May 2021. We included diagnostic studies on urine or blood biomarkers for cystitis or pyelonephritis in children below 18 years of age. We calculated sensitivity, specificity and likelihood ratios. Data were pooled using a bivariate random effects model and a Hierarchical Summary Receiver Operating Characteristic analysis.

**Results:**

Seventy-five moderate to high quality studies were included in this review and 54 articles in the meta-analyses. The area under the receiver-operating-characteristics curve to diagnose cystitis was 0.75 (95%CI 0.62 to 0.83, *n* = 9) for C-reactive protein, 0.71 (95% CI 0.62 to 0.80, *n* = 4) for procalcitonin, 0.93 (95% CI 0.91 to 0.96, *n* = 22) for the dipstick test (nitrite or leukocyte esterase ≥trace), 0.94 (95% CI 0.58 to 0.98, *n* = 9) for urine white blood cells and 0.98 (95% CI 0.92 to 0.99, *n* = 12) for Gram-stained bacteria. For pyelonephritis, C-reactive protein < 20 mg/l had LR- of 0.10 (95%CI 0.04–0.30) to 0.22 (95%CI 0.09–0.54) in children with signs suggestive of urinary tract infection.

**Conclusions:**

Clinical prediction rules including the dipstick test biomarkers can support family physicians while awaiting urine culture results. CRP and PCT have low accuracy for cystitis, but might be useful for pyelonephritis.

**Supplementary Information:**

The online version contains supplementary material available at 10.1186/s12875-021-01530-9.

## Introduction

Paediatric urinary tract infections (UTI) could be considered serious since they may trigger systemic infection and result in kidney scarring [1]. UTIs occur in nearly 6% of all acutely ill children presenting to ambulatory care [2]. Prompt diagnosis and treatment is vital to prevent renal injury [3, 4]. Infants with kidney involvement are at risk of complications such as bacteraemia or meningitis [5]. Antibiotic treatment should be guided by urine culture results in childhood UTI, however test results are generally only available 24 h after sampling [3].

Rapid urine tests might improve early diagnosis and might reduce the use of ineffective antibiotics [6]. Two other systematic reviews have been published on the diagnostic accuracy of urine biomarkers for UTIs in children [7, 8]. No single rapid urine test was found that could replace urine culture. Urine Gram-stain was the most accurate test; however, in practice it is not routinely performed in ambulatory care settings, due to logistic challenges. These reviews were published more than 10 years ago (searched until 2009), and did not include study data from ambulatory care settings separately.

In children, a blood sample via finger prick testing can be easily obtained. At present, point-of-care tests are available that measure biomarkers such as C-reactive protein (CRP) or procalcitonin (PCT) on a droplet of blood accurately and rapidly [9]. Such rapid tests might be useful for ruling out UTI in children presenting to ambulatory care.

The aim of this review was to collate the most recent evidence on the diagnostic value of urine or blood biomarkers for paediatric UTIs in ambulatory care settings, in order to identify the most useful combination of clinical features and laboratory tests to rule in or rule out UTI, with confidence.

## Method

### Protocol registration

The protocol was registered a priori on Prospero (CRD42019122174) and the study is reported following the Preferred Reporting Items for Systematic Reviews and Meta-analyses guidelines (Additional file 1).

### Information sources

Six electronic databases (Medline, Embase, WOS, DARE and HTA, Cochrane library and CINAHL) were searched using a comprehensive search strategy which was developed in close collaboration with a biomedical librarian and included both indexed terms as well as free text (Additional file 2). We conducted the search on 16 January 2019, 27 January 2020 and 21 May 2021. Additionally, we checked the references of systematic reviews [7, 8, 10, 11] and guidelines [3, 4, 12]. Five reviewers individually selected studies in pairs (HB, TS, JV, AVdB, AG) and two reviewers (JV, AVdB) resolved conflicts independently. The full text screening was performed independently in pairs (HB, TS). A list of excluded studies with reason why is provided in Additional file 3. We deduplicated studies in Endnote X8.2 (Clarivate Analytics, USA) and used the Covidence online software for study selection (Veritas Health Innovation, Australia).

### Eligibility criteria

We included all studies that compared the diagnostic accuracy of urine or blood biomarkers for UTI in children below 18 years of age. We defined cystitis as bacterial growth on urine culture, pyelonephritis as changes on DMSA scan or Ultrasound and bacteraemia with associated UTI as growth of the same pathogen on urine culture and blood culture. Only studies in acutely ill children were included, excluding studies in healthy children. Eligible study designs were prospective cross-sectional diagnostic accuracy studies, nested case control studies and retrospective cohort studies. Ambulatory care was defined as family practices, emergency departments, walk-in clinics, health centres, and hospital outpatient departments.

We excluded studies in children from high risk groups (malnourished, neurogenic bladder) or in admitted children. We excluded case-control studies, letters, comments, and conference abstracts. Additionally, studies with a total sample size < 50 were excluded because those studies are more prone to selection bias [13, 14]. We did not apply any language, time or country restrictions.

### Data collection

Two reviewers extracted 2 × 2 tables (=true positives, false positives, false negatives and true negatives) for each biomarker in duplicate together with the study characteristics (HB, AG). If information was missing, we contacted the study authors (*n* = 36). Eight authors provided non-published data [15–23]. We excluded multiple publications based on the same study results (same study authors, study period, setting and index tests). If a 2 × 2 table contained a cell that had a zero value, we applied a continuity correction (replaced 0 by 0.5). If no threshold was reported for leucocyte esterase (LE), we assumed any discoloration as the positivity threshold for that particular study (‘trace’). Thresholds for urine leukocytes, measured with automatic urine microscopy were converted to microliter (μl) according to the manufacturer’s instructions [24, 25].

### Quality assessment

We assessed risk of bias with the Quality Assessment of Diagnostic Accuracy Studies criteria (QUADAS-2) using Revman version 5.3 (The Cochrane Collaboration Review Manager, Copenhagen). HB and AG assessed the risk of bias and applicability independently and disagreements were discussed during a consensus meeting (HB, TS, AVdB, JV). All retrospective studies were considered at high risk for selection bias, because those studies might overestimate the diagnostic accuracy of the index test [26]. We referred to urine culture thresholds used in guidelines for assessing the risk of bias for the reference standard [3, 4, 12].

### Data analysis

We used R statistical software version 3.5.1 (R Foundation, Austria) to calculate sensitivity, specificity and likelihood ratios for UTI (mada package in R version 0.8.5). We provided likelihood ratios in dumbbell plots displaying the change in disease probability following a positive or negative test (GitHub, Susannah Fleming) [27, 28]. We considered biomarkers or clinical prediction rules useful for ruling out UTI if their negative likelihood ratios (LR-) were ≤ 0.25 and useful to rule in UTI if their positive likelihood ratio (LR+) was ≥4 [29, 30]. We further specified LR+ between 1 to 2, 2 to 5, 5 to 10, and > 10 as a ‘slight’, ‘moderate’, ‘large’ and ‘very large’ increase in probability (considered as ‘red flags’). LR- between 1 to 0.5, 0.5 to 0.2, 0.2 to 0.1, and < 0.1 were interpreted as a ‘slight’, ‘moderate’, ‘large’, and ‘very large’ decrease in probability [4, 29].

We estimated summary parameters using a bivariate random effects meta-analysis for biomarkers assumed to be dichotomous (e.g. nitrite) whenever three or more primary studies were available [31]. If we suspected substantial clinical heterogeneity of a specific study, we excluded that study from the meta-analysis. When multiple thresholds where reported for continuous biomarkers (e.g. CRP), we conducted a Hierarchical Summary Receiver Operating Characteristic (HSROC) meta-analysis and calculated the area under the receiver operating characteristic curve (AUC) (diagmeta package in R version 0.4) [32]. For the LR’s derived from the HSROC model, we used bootstrapping (coxed package in R version 0.3.3) to construct 95% confidence intervals (95% CI).

To assess statistical heterogeneity, we inspected the dumbbell plots, conducted chi-square testing and performed subgroup analyses by adding covariates in a meta-regression if ten or more studies were available for this analysis. We performed subgroup analyses for design, prevalence and urine collection method. Additionally, we conducted sensitivity analyses to check the robustness of our results by excluding outlying values or data whenever we suspected clinical heterogeneity.

## Results

### Study selection and characteristics

**Fig. 1:**
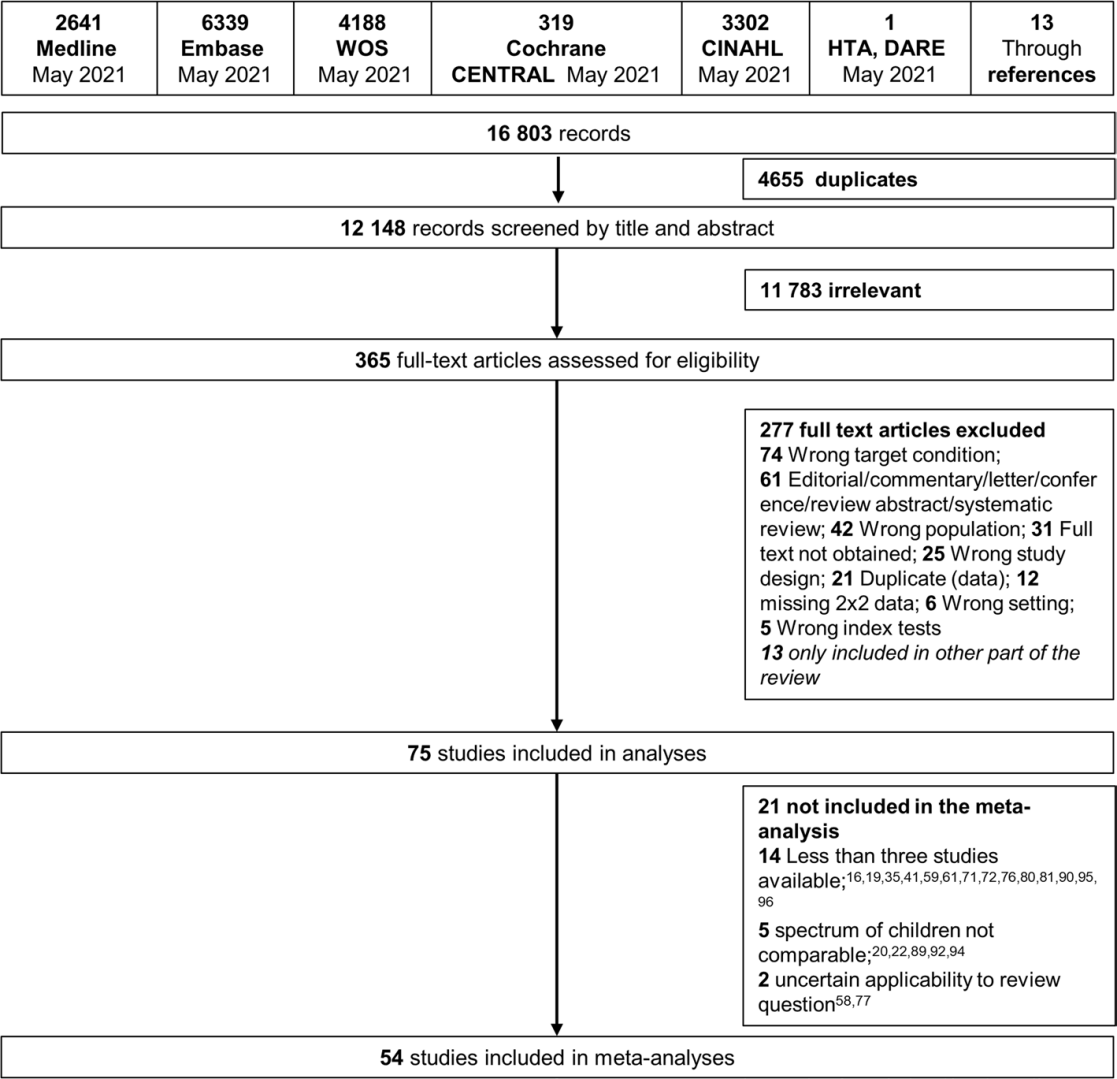
PRISMA flow diagram of included studies. PRISMA = Preferred Reporting Items for a Systematic review and Meta-analysis of Diagnostic Test Accuracy Studies

We screened 12,148 studies, of which we evaluated 355 on full text (Fig. 1). Ultimately, we included 75 studies reporting the diagnostic accuracy of 20 urine biomarkers (*n* = 60) [15, 18, 23–25, 33–87], four blood biomarkers (*n* = 15) [17, 18, 20–22, 40, 83, 84, 88–94], and four prediction rules (*n* = 4) [15, 16, 19, 70, 95, 96].

Most studies were performed at the emergency department (*n* = 53), while other settings were outpatient departments (*n* = 12), health centres (*n* = 7) or mixed settings including family practices (*n* = 3). Data from 54 studies were included in the meta-analysis, of which 40 studies had a prospective design. We included 67 studies on cystitis [15–18, 21, 23–25, 33–60, 62–88, 91, 93, 95, 96], seven on pyelonephritis [20, 22, 61, 89, 92–94], and four on bacteraemia with associated UTI [19, 36, 65, 90]. The total number of included patients was 117,531 for UTI, 628 for pyelonephritis, and 6320 for bacteraemia. Median prevalence of cystitis was 12.0% (range 1.3 to 67.5%) and for pyelonephritis, prevalence was as high as 62.9 to 72.2% in children with a positive urine dipstick test or growth on urine culture.

Studies on the diagnosis of cystitis were either in acutely ill children (*n* = 4), febrile children (*n* = 9), children with signs suggestive of UTI or suspicion of UTI by the physician (*n* = 18), or children for whom an additional urine sample or test result was available (*n* = 36).

Prediction rules for diagnosis of cystitis, were based on clinical features together with the urine dipstick test biomarkers [15, 16, 95] or blood biomarkers with urinalysis [96]. All study characteristics are listed in more detail in Additional file 4.

### Diagnostic accuracies

Table 1 shows the summary of findings table, while Additional file 5 shows the dumbbell plots. Table 2 shows the variables of each clinical prediction rule.

**Table 1:** Summary of findings

**Which biomarkers and predictions rules are useful for ruling out UTI in children?**							
**Population** acutely ill children < 18 years of age							
**Index test** biomarkers or clinical prediction rules							
**Reference standard** urine culture (cystitis), DMSA scan (pyelonephritis)							
**Target condition** urinary tract infection (cystitis, pyelonephritis)							
**Setting** primary care, outpatient							
**Positive likelihood ratio (LR+)**				**Negative likelihood ratio (LR-)**			
**> 10** = very large increase in probability				**0–0.1** = very large decrease in probability			
**5–10** = large increase in probability				**0.1–0.2** = large decrease in probability			
**2–5** = moderate increase in probability				**0.2–0.5** = moderate decrease in probability			
**1–2** = slight increase in probability				**0.5–1** = slight decrease in probability			
**Outcome**	**Biomarkers**	**Number of patients** (Number of studies)	**LR+ (95%CI)**	**LR- (95%CI)**	**AUC (95%CI)**	**Quality of the evidence (**GRADE**)**	**Comments**
**Cystitis** (positive urine culture)	N	55,402 (26^a^)	39 (21–73)	0.61 (0.55–0.68)	0.79 (0.63–0.91)	⨁⨁⨁◯ due to indirectness	^d^
	LE	65,204 (26^b^)			0.94 (0.80–0.98)	⨁⨁⨁◯ due to indirectness and risk of bias	^d^
	trace		8 (6–14)	0.13 (0.09–0.25)			
	+		13 (9–19)	0.16 (0.11–0.31)			
	++		28 (15–114)	0.24 (0.14–0.76)			
	+++		61 (26–994)	0.35 (0.15–0.96)			
	N or LE	25,238 (22^a^)	9 (6–13)	0.13 (0.10–0.18)	0.93 (0.91–0.96)	⨁⨁⨁◯ due to indirectness	^d^
	N and LE	38,070 (10^a^)	115 (33–394)	0.65 (0.62–0.68)	0.51 (0.38–0.83)	⨁⨁⨁◯ due to indirectness	^d^
	Protein ≥trace	665 (3^a^)	3 (2–3)	0.69 (0.56–0.83)	0.70 (0.47–0.91)	⨁◯◯◯ due to inconsistency, indirectness and imprecision	^d^
	WBC ≥5/hpf (manual)	21,763 (18^b^)	6 (6–25)	0.27 (0.22–0.69)	0.90 (0.77–0.98)	⨁⨁⨁◯ due to indirectness	^d^
	WBC ≥10/μL (automatic)	56,286 (9^b^)	4 (2–6)	0.13 (0.06–0.23)	0.91 (0.58–0.98)	⨁⨁⨁◯ due to indirectness	^d^
	B ≥ 1/hpf	2979 (6^b^)	3 (2–8)	0.11 (0.06–0.24)	0.93 (0.25–1.00)	⨁◯◯◯ due to risk of bias, indirectness and imprecision	^d^
	Gram stain ≥1/hpf	13,945 (12^b^)	20 (14–34)	0.10 (0.06–0.15)	0.98 (0.92–0.99)	⨁⨁⨁⨁	^d^
	CRP ≥10 mg/L	13,729 (9^b^)	2 (1–2)	0.38 (0.24–0.77)	0.75 (0.62–0.83)	⨁⨁◯◯ due to inconsistency and indirectness	
	PCT ≥0.25 ng/mL	6585 (4^b^)	2 (2–8)	0.56 (0.24–0.69)	0.71 (0.62–0.80)	⨁⨁◯◯ due to risk of bias and indirectness	^e^
	DUTY (+LE, N, Hb) ≥5 points	2277 (1^c^)	5 (5–6)	0.22 (0.13–0.37)	/	⨁⨁⨁◯ due to imprecision	
	UTIcalc (+LE, N)	229 (1^c^)	18 (10–33)	0.05 (0.01–0.26)	/	⨁◯◯◯ due to indirectness and imprecision	^f^
**Pyelonephritis** (positive DMSA scan)	WBCc 12 to 20 × 10^3^/μL	209 (2^c^)	1.62 to 3.57	0.35 to 0.83	/	⨁◯◯◯ due to serious indirectness and imprecision	^g^
	CRP ≥20 mg/L	7179 (5^c^)	1.32 to 2.74	0.10 to 0.37	/	⨁⨁◯◯ due to imprecision and indirectness	
	PCT ≥2 ng/mL	436 (5^c^)	2.93 to 14.22	0.32 to 0.74	/	⨁◯◯◯ due to inconsistency, indirectness and imprecision	^g^

GRADE Working group grades of evidence

High quality: further research is very unlikely to change our confidence in the estimate of effect. ⨁⨁⨁⨁

Moderate quality: further research is likely to have an important impact on our confidence in the estimate of effect and may change the estimate. ⨁⨁⨁◯

Low quality: Further research is very likely to have an important impact on our confidence in the estimate of effect and is likely to change the estimate. ⨁⨁◯◯

Very low quality: We are very uncertain about the estimate. ⨁◯◯◯

*DMSA* Dimercaptosuccinic acid scan, *95%CI* 95% confidence intervals, *AUC* Area Under the Receiver Operating Characteristic (ROC) Curve Analysis, Assessment, Development and Evaluations, *N* Nitrite (urine), *LE* Leukocyte esterase (urine), *Hb* Hemoglobin (urine), *WBC* White blood cell (urine), *B* Unstained bacteria (urine), *CRP* C-reactive protein (blood), *PCT* Procalcitonin (blood), *DUTY* Diagnosis of Urinary Tract Infections in Young children, *UTIcalc* UTI calculator, *hpf* High-power field, *μL* Microliter, *mg/L* Milligram per liter, *ng/mL* Nanogram per milliliter, *mm*^*3*^ Cubic milliliter

^a^Bivariate random effects model

^b^HSROC model

^c^Descriptive statistics

^d^The majority of studies included patients with suspicion of UTI or with UTI features, and therefore the results might not be applicable for patients without suspicion of UTI or UTI features

^e^Majority of children had fever without a source for infection

^f^Derived in setting where there was high circumcision rate in boys

^g^In children with high pre-test probability of UTI

**Table 2:** Variables of clinical prediction rules for urinary tract infections

**Clinical prediction rule**	**Variables**
**UTIcalc score**	Clinical features together with dipstick test (leukocyte esterase and nitrite), and/or urine white blood cells and/or gram stain
	- Age < 12 m
	- Temperature ≥ 39 °C
	- Nonblack race
	- Female or uncircumcised mail
	- Other fever source
**DUTY score, clean catch samples**	Clinical features with dipstick test:
	- Pain or crying while urinating (2 points)
	- Smelly urine (2 points)
	- History of UTI (1 point)
	- Absence of cough (2 points)
	- Score > 6 on severity of illness scale 0–10 (2 points)
	- Leukocyte esterase positive (2 points)
	- Nitrite positive (3 points)
	- Blood positive (1 point)
**DUTY score, nappy pad samples**	Clinical features with dipstick test:
	- Female gender (1 points)
	- Smelly urine (2 points)
	- Darker urine (1 point)
	- No nappy rash (4 points)
	- Leukocyte esterase positive (2 points)
	- Nitrite positive (3 points
**Kuppermann score**	≥1 abnormal blood or urine biomarkers:
	- Absolute neutrophil count ≤4090/μL
	- Procalcitonin ≤1.71 ng/mL
	- Leukocyte esterase ≥trace
	- Nitrite positive
	- White blood cells ≥5/hpf
**NICE traffic light score**	Amber or red colour on the NICE traffic light score or urine dipstick positive

*M* Months, *°C* Degrees Celsius, *μL* Microliter, *ng/mL* Nanogram per milliliter, *hpf* High power-field

#### Diagnosis of cystitis

For ruling out cystitis, a urine dipstick with both a negative result for LE and nitrite corresponds with a LR- of 0.11 (95% CI 0.08–0.17, *n* = 7) for low prevalence studies (< 10%) [53, 62–65, 69, 79]. No dipstick biomarker combination provides a very large decrease in probability, e.g. all LR-‘s were ≥ 0.10.

**Fig. 2:**
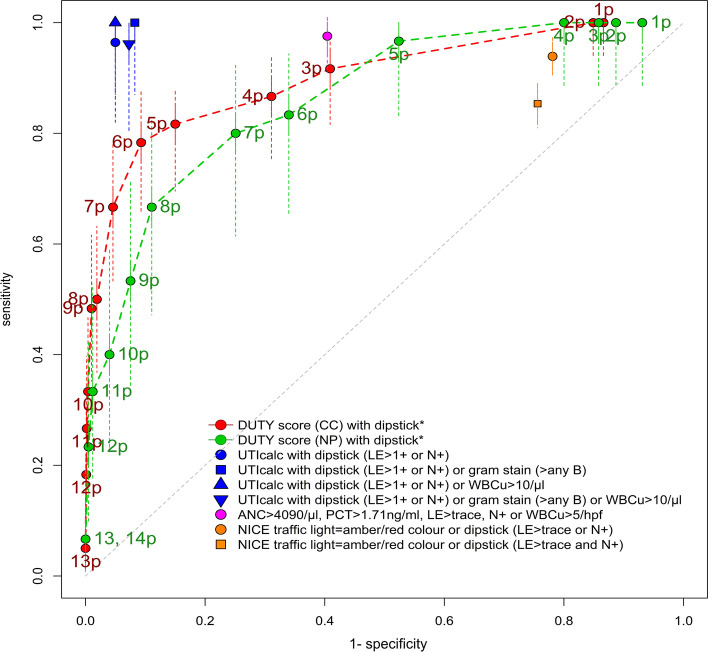
ROC curve of clinical prediction rules for cystitis. Receiver Operating Characteristic Curve (ROC) analysis showing sensitivity versus 1-specificity at each threshold. The thresholds for a positive rule are shown next to each point on the graph. Each colour represents the diagnostic test accuracy of one prediction rule for urinary tract infection in children. Confidence intervals of the estimates (sensitivities) are indicated as dashed lines. p = points; DUTY = diagnosis of urinary tract tractions in young children; CC = clean catch samples; NP = nappy pad samples; UTI = urinary tract infection; PCT = procalcitonin; ANC = absolute neutrophil count; NICE = National Institute for Health and Care Excellence; LE = Leucocyte Esterase, N = Nitrite, WBCu = urine white blood cells, B = urine bacteria, hpf = high power field, μl = microliter, ng = nanogram, ml = milliliter, derivation studies are indicated with an asterisk (*)

Interpreting the urine dipstick biomarker results as part of the UTIcalc score further decreases the LR- to 0.05 (95% CI 0.01–0.26) for cystitis [15, 16]. The UTIcalc score incorporates age < 12 months, temperature ≥ 39 °C, non-African American, female or uncircumcised male, and other fever source and was the most useful prediction rule for both diagnosing and ruling out UTI (Fig. 2). The DUTY score ≥ 5 points has a LR+ of 5.41 (95%CI 4.65 to 6.28) and LR- of 0.22 (95%CI 0.13 to 0.37). The NICE traffic light system with the dipstick biomarkers gives a LR- of 0.28 (95% CI 0.19–0.42) [95] and a diagnostic tree by Kuppermann et al. (ANC ≤4090/μl, PCT ≤1.71 ng/ml, LE and nitrite negative, WBCu < 5/hpf) a LR- of 0.05 (95% CI 0.01–0.17) in children < 60 days old [96].

Either nitrite or LE (LR+ 7.09 95% CI 3.81–13.18, *n* = 7) and WBCu on manual microscopy ≥5/hpf (LR+ 29.21, 95% CI 11.05–54.12, *n* = 7) are red flags for cystitis in settings with low pre-test probability (< 10%).

For diagnosing cystitis if pre-test probability is higher (≥10%), nitrite, WBCu ≥10/μl on automatic microscopy, or Gram stained bacteria give a very large increase in post-test probability. Presence of nitrite increases post-test probability at all ages at a LR+ of 38.34 (95% CI 18.49–79.50, *n* = 16) for children below 5 years of age. Neutrophilic Gelatinase Associated Lipocalin (NGAL) ≥39.1 ng/ml, a protein found in urine, might be very accurate for both diagnosing (LR+ of 20.73, 95% CI 11.38–37.38) and ruling out cystitis (LR- of 0.04, 95% CI 0.01–0.21), based on one study [51]. Human Neutrophilic Peptides (HNP) 1–3 and Human Defensin (HD) 5 give a LR- of 0.08 (95% CI 0.02–0.37) and 0.03 (95% CI 0.00–0.40) [68].

WBCc ≥15,000/μl or ≥ 17,400/μl increase probability of cystitis moderately, giving a LR+ of 2.33 to 2.79 in children <5 years of age [17, 18, 84]. ANC ≥10,000/μl gives a LR+ of 4.07, 95% CI 3.38–4.90 [18]. Low WBCc or ANC decrease post-test probability of UTI only slightly, with a LR- of 0.61 (95% CI 0.56–0.66) to 0.78 (95% CI 0.74–0.81) [18].

CRP ≥55 mg/l or 80 mg/l give a LR+ of 3.56 (95% CI 2.02–5.12, *n* = 9) and 4.38 (95% CI 2.02–5.13, *n* = 9) for cystitis. If CRP is not elevated (< 5 mg/l), the LR- is 0.35 (95% CI 0.26–0.63, *n* = 3) when pre-test probability is low (< 10%) [17, 21, 91]. PCT ≥2 ng/ml gives a LR+ of 4.19 (95% CI 3.72–17.53, *n* = 4) and LR- of 0.79 (95% CI 0.57–0.94, *n* = 4), while the lowest thresholds (≥1 ng/ml) corresponds to a LR+ of 2.01 (95%CI 1.91–6.68, *n* = 4) and LR- of 0.53 (95% CI 0.29–0.70, *n* = 4) [18, 21, 88, 91].

#### Diagnosis of pyelonephritis

**Fig. 3:**
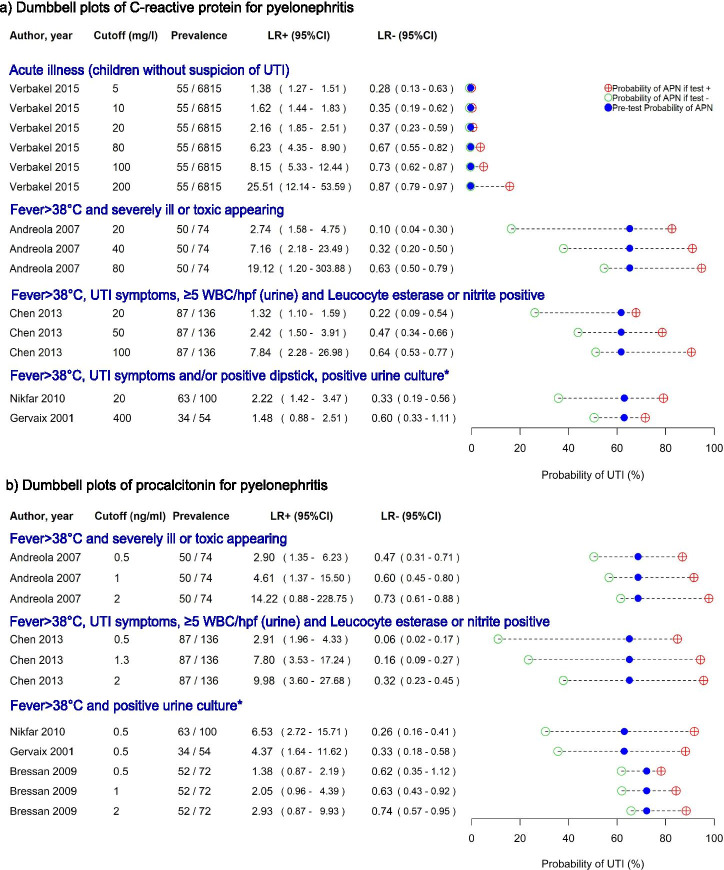
Likelihood ratios and post-test disease probabilities for pyelonephritis (dumbbell plots). UTI = urinary tract infection; n = sample size, Prev = prevalence; 95%CI = 95% Confidence Interval; LR+ = positive likelihood ratio; LR- = negative likelihood ratio, mg = milligram, ml = milliliter ng = nanogram, °C = degrees Celsius, WBC = white blood cells (urine), hpf = high power field, *positive urine culture = growth of one uropathogen

The dumbbell plots of blood tests for pyelonephritis are shown in Fig. 3. In febrile children that are seriously ill, CRP < 20 mg/l gives a LR- of 0.10 (95% CI 0.04–0.30, *n* = 1) for pyelonephritis [20]. In febrile children with a positive urine dipstick test and ≥ 5WBCu/hpf, CRP < 20 mg/l corresponds with LR- of 0.22 (95% CI 0.09–0.54, *n* = 1) [94]. In children without signs suggestive of UTI, or with confirmed urinary infection, CRP < 20 mg/l gave LR-‘s above 0.25.

PCT < 0.5 ng/ml lowers the probability of pyelonephritis moderately, (LR- 0.26 to 0.62) in febrile children with a positive urine culture. PCT ≥0.5 ng/ml gives a moderate to large increase in probability of pyelonephritis [20, 89, 92, 94]. WBCc < 16,500/μl or 15,000/μl and ANC < 10,000/μl give a slight to moderate decrease in probability of pyelonephritis [20, 94]. (Supplementary Figure S16).

### Quality assessment

Bias was present for patient selection, caused by retrospective sampling (*n* = 20) [16, 19, 24, 34–36, 38, 43, 50, 52, 53, 69, 71, 74, 76, 81, 82, 86, 88, 90], convenience sampling (*n* = 8) [21, 37, 39, 41, 46, 51, 61, 65], or including a narrow spectrum of patients (*n* = 4) [72, 77, 83, 90]. All retrospective studies were considered at high risk for selection bias, because these studies might overestimate the diagnostic accuracy of the index test [26]. In five studies, biomarker thresholds were not pre-specified [17, 61, 70, 83, 90], and in nine studies, culture thresholds were not adapted for the collection method [18, 21, 41, 47, 57, 69, 70, 72, 78]. Bias in flow and timing was caused by partial verification (*n* = 7) [17, 20, 61, 87, 91, 93, 95], differential verification (*n* = 2) [33, 44], or inappropriate exclusions from the analyses (*n* = 3) [65, 77, 90] (Additional file 6).

### Additional analyses

Statistical heterogeneity between studies was present (*p* < 0.001), however subgroup analyses for design, collection method, or prevalence were not statistically significant (*p*-values ≥0.11). The LR+ of CRP for cystitis varied between the primary studies. One prospective study reported CRP ≥ 20 mg/l in children <3 months to correspond with a LR+ of 12.49 (95% CI 6.27–24.86) while other studies found a LR+ of 4.25 (95% CI 3.84–4.70) for the same threshold. Exclusion of studies with UTI prevalence above 10% gave an AUC for CRP of 0.76 (95% CI 0.45–0.92, *n* = 3), exclusion of retrospective studies an AUC of 0.76 (95% CI, 0.64–0.84, *n* = 7). For PCT, excluding one retrospective study with UTI prevalence above 20% gave an AUC of 0.72 (95% CI 0.47–0.85, *n* = 3) for cystitis.

## Discussion

### Summary

The UTIcalc incorporating the dipstick biomarkers is an accurate (LR- of 0.05) and relatively simple alternative for primary care, low-resource settings or situations where a consultation in-person is impractical. Parents might assess symptoms and demographic features of clinical prediction rules at home or in the waiting room before the consultation.

Other biomarkers that are useful for ruling out and ruling in cystitis are urine Gram stain (LR- 0.10, LR+ 19.67) and LE with nitrite present on the urine dipstick test (LR- 0.13, LR+ 8.08). Biomarkers such as NGAL < 39.1 ng/ml and HD5 < 174 mg/mgCr might be very useful for ruling out (LR- ≤0.05), based on one study. WBC on manual microscopy (≥5/hpf) (LR+ 6.25) moderately increases the probability of cystitis.

Systemic inflammatory markers, such as CRP and PCT offer little added value for cystitis (AUC 0.75 and 0.71), whereas in children with signs suggestive of UTI, CRP < 20 mg/l might be useful for ruling out and PCT ≥2 ng/ml for ruling in pyelonephritis. Other blood markers, such as WBCc and ANC, are less useful for diagnosing pyelonephritis.

### Strengths and limitations

The main strength of our study was the selection of ambulatory care studies by a comprehensive search to provide relevant information for primary care physicians in low-prevalence settings such as family practices, emergency departments, health centres and outpatient departments where ruling out UTIs is most important.

The prevalence of cystitis varied between the studies and there were no studies available that were performed in family practice only. The majority of studies were performed at the emergency department. To limit the potential impact of spectrum bias on the results, we performed separate analyses on studies with pre-test probability < 10%, and excluded two studies from the meta-analyses where we suspected very low applicability for family practice [58, 77]. There was low risk of bias due to non-consecutive recruitment, as only eight studies included a convenience sample. Most studies (*n* = 43) included children with either UTI symptoms, fever without source or suspicion by the treating physician, making the results less applicable for children that do not present with UTI features or have low suspicion of UTI.

For pyelonephritis, it was not feasible to pool results due to the heterogeneity regarding patient selection. We therefore restricted the analyses to descriptive statistics for this outcome. New studies should investigate the accuracy of CRP for pyelonephritis at lower thresholds (< 10 mg/l or < 5 mg/l) in children with signs suggestive of UTI.

### Comparison with existing literature

In this study, we confirmed that bacteria on urine Gram stain is the most accurate biomarker compared to urine culture, however Gram stain is not feasible to perform systematically on sampled urine in ambulatory care settings [8].

Previous reviews on UTI with searches up to 2009 were limited by merely investigating urine biomarkers and not providing results for outpatient settings separately [7, 8]. We found only one systematic review including studies on blood biomarkers for UTI, however only one study on CRP and no studies on PCT were available at that time. For LE, we provided summary estimates per threshold separately (>trace, 1+, 2+, 3+) whereas previous reviews with meta-analyses only provide results for >trace.

Other studies found that devices for rapid antibiotic susceptibility testing might be useful [97, 98], or other technologies to detect bacteria such as colorimetric systems [99], FISH, MALDI-TOF and multiplex PCR [100]. The applicability of these findings for children in the outpatient setting remains unclear and most of these tests still require 4 to 12 h before the results are available.

### Implications for research and practice

Nitrite and LE have good diagnostic value compared to urine culture in children presenting to primary care with signs or symptoms of UTI. Using a clinical prediction rule such as the UTIcalc score together with the dipstick test is useful to support decision-making while awaiting urine culture results. Whenever the UTIcalc score with dipstick test is negative, 99.7% of urine cultures could be avoided, while 3% of UTIs will be missed compared to 12% when only using the dipstick test.

Systemic inflammatory markers such as WBCc, ANC, CRP and PCT offer little additional value for cystitis, whereas for pyelonephritis, CRP < 20 mg/l is useful for ruling out and PCT ≥ 2 ng/ml for ruling in.

Future research should focus on validating the UTIcalc and DUTY score or assess the usefulness of clinical prediction rules performed at home by parents or as part of telemedicine visits.

## Supplementary Information

**Additional file 1: Table S1.** PRISMA - Diagnostic test Accuracy Studies checklist.

**Additional file 2: Table S2.** Electronic search strategy (Embase).

**Additional file 3: Table S3.** List of excluded studies with reasons why (full text screening) (*n* = 277).

**Additional file 4: Table S4.** Characteristics of included studies.

**Additional file 5: Figures S1–21.** Dumbbell plots of biomarkers, point-of-care tests and prediction rules for urinary tract infection.

**Additional file 6: Figures S22–23.** Risk of bias and applicability assessment.

## Data Availability

Full datasets can be obtained from the corresponding author on reasonable request. JV affirms that the manuscript is an honest, accurate and transparent account of the study being reported; that no important aspects of the study have been omitted; and that any discrepancies from the study as planned (and, if relevant, registered) have been explained.
