# Supplementary material for: Diagnostic value of biomarkers for paediatric urinary tract infections in primary care: systematic review and meta-analysis
Source: BMC Fam Pract. 2021 Sep 27;22:193. doi: 10.1186/s12875-021-01530-9 (PMC8474745; doi:10.1186/s12875-021-01530-9)
Supplement: Supplementary file 2 — Additional file 2: Table S2. Electronic search strategy (Embase). [file 12875_2021_1530_MOESM2_ESM.docx]

**Additional file 2. (Table S2.** Electronic search strategy (Embase)

**Table S2: Search strategy (Embase)**

The other search strategies for Medline (through Pubmed), Web of Science, Cochrane library, Cinahl and HTA/DARE are available from the corresponding author on request.

| **Concept 1: Urinary tract infections (bladder, kidney)** |
| --- |
| "Urinary Tract Infections"[Mesh] OR "Infection/urine"[Mesh] OR "cystitis"[Mesh] OR "pyelitis"[Mesh] OR "Pyonephrosis"[Mesh] OR "Pyelonephritis"[Mesh] OR "Urethritis"[Mesh] OR urinary-tract-infection*[tiab] OR UTI[tiab] OR ((infection*[tiab] OR inflam*[tiab]) AND (bladder[tiab] OR kidney[tiab] OR “urinary tract”[tiab])) OR bacteriuria*[tiab] OR pyuria*[tiab] OR bladder-infection*[tiab] OR “bladder inflammation”[tiab] OR cystitis[tiab] OR cystitides[tiab] OR urethritis[tiab] OR pyelocystitis[tiab] OR cystopyelitis[tiab] OR kidney-infection*[tiab] OR pyelitis[tiab] OR pyelonephritis[tiab] OR pyelonephritides[tiab] OR urosepsis[tiab] OR pyonephrosis[tiab] |
| **Concept 2: Signs and symptoms, diagnostic tests, clinical prediction rules** |
| "Signs and Symptoms"[Mesh] OR "Diagnostic Techniques and Procedures"[Mesh] OR "Diagnosis"[Mesh:NoExp] OR "Clinical Decision-Making"[Mesh] OR "Decision Support Techniques"[Mesh] OR "Decision Trees"[Mesh] OR "diagnosis"[Subheading] OR "Reagent Kits, Diagnostic"[Mesh] OR "Point-of-Care Systems"[Mesh] OR "Biomarkers"[Mesh] OR "C-Reactive Protein"[Mesh] OR "Lactic Acid"[Mesh] OR "Procalcitonin"[Mesh] OR "Nitrates/urine"[Mesh] OR "leukocyte esterase" [Supplementary Concept] OR "Peroxidase/urine"[Mesh] OR "Lactoferrin/urine"[Mesh] OR "Immunoglobulin A, Secretory/urine"[Mesh] OR diagnos*[tiab] OR sign[tiab] OR signs[tiab] OR symptom*[tiab] OR clinical-feature*[tiab] OR clinical-assessment[tiab] OR anamnesis[tiab] OR “medical history”[tiab] OR symptom-evaluation*[tiab] OR symptom-assessment*[tiab] OR physical-examination*[tiab] OR clinical-examination*[tiab] OR clinical-impression*[tiab] OR intuition[tiab] OR “gut feeling”[tiab] OR prediction-rule*[tiab] OR decision-tree*[tiab] OR decision-support-techni*[tiab] OR decision-model*[tiab] OR decision-support-model*[tiab] OR apgar-score*[tiab] OR visual-analogue-scale*[tiab] OR “generally unwell”[tiab] OR lethargy[tiab] OR consciousness[tiab] OR confusion[tiab] OR disorientation[tiab] OR convulsion*[tiab] OR irritability[tiab] OR edema[tiab] OR deshydration[tiab] OR jaundice[tiab] OR pallor[tiab] OR sleepiness[tiab] OR capillary-refill-time*[tiab] OR vital-sign*[tiab] OR parameter*[tiab] OR saturation*[tiab] OR heart-rate*[tiab] OR pulse*[tiab] OR blood-pressure*[tiab] OR body-temperature*[tiab] OR fever[tiab] OR pyrexia[tiab] OR shivering[tiab] OR chills[tiab] OR hypotherm*[tiab] OR respiratory-rate*[tiab] OR dyspnea[tiab] OR hypoxia[tiab] OR tachypnea[tiab] OR cyanosis[tiab] OR “failure to thrive”[tiab] OR failure-to-thrive[tiab] OR weight[tiab] OR feeding[tiab] OR “fluid intake”[tiab] OR constipation[tiab] OR diarrhea[tiab] OR nausea[tiab] OR vomiting[tiab] OR “urine appearance”[tiab] OR oliguria[tiab] OR polyuria[tiab] OR dysuria[tiab] OR mictalgia[tiab] OR “malodorous urine”[tiab] OR “cloudy urine”[tiab] OR “smelly urine”[tiab] OR incontinence[tiab] OR stranguria[tiab] OR frequency[tiab] OR urgency[tiab] OR haematuria[tiab] OR hematuria[tiab] OR “flank pain”[tiab] OR “back pain”[tiab] OR “suprapubic pain”[tiab] OR “suprapubic discomfort”[tiab] OR “abdominal pain”[tiab] OR “abdominal tenderness”[tiab] OR “costovertebral angle pain”[tiab] OR “costovertebral angle tenderness”[tiab] OR palpation[tiab] OR “painful kidney”[tiab] OR “palpable kidney”[tiab] OR percussion[tiab] OR uncircumcis*[tiab] OR “dysplastic kidney”[tiab] OR "Vesico-Ureteral Reflux"[tiab] OR laboratory-technique*[tiab] OR laboratory-test*[tiab] OR lab-test*[tiab] OR hematology-test*[tiab] OR hematological-test*[tiab] OR hematologic-test*[tiab] OR blood-test*[tiab] OR blood-gas*[tiab] OR point-of-care-test*[tiab] OR point-of-care-system*[tiab] OR POC-test*[tiab] OR POCT[tiab] OR near-patient-test*[tiab] OR rapid-test*[tiab] OR point-of-care-techn*[tiab] OR bedside-test*[tiab] OR biomarker*[tiab] OR marker*[tiab] OR biochemical-marker*[tiab] OR immunologic-test*[tiab] OR immunological-test*[tiab] OR “CRP”[tiab] OR “c reactive protein”[tiab] OR “c-reactive protein”[tiab] OR procalcitonin[tiab] OR nitrate[tiab] OR leucocyte[tiab] OR “erythrocyte sedimentation”[tiab] OR “glomerular filtration”[tiab] OR GFR[tiab] OR urea[tiab] OR lactoferrin[tiab] OR antimicrobial-peptide*[tiab] OR myeloperoxidase[tiab] OR interleukin*[tiab] OR “xanthine oxidase”[tiab] OR heparin-binding-protein*[tiab] OR “secretory IgA”[tiab] OR proteinase-inhibitor*[tiab] OR electrolyte*[tiab] OR “full blood count”[tiab] OR FBC[tiab] OR “lactic acid”[tiab] OR LDH[tiab] OR lactate[tiab] OR urine-test*[tiab] OR urinalysis[tiab] OR cytology[tiab] OR kidney-function-test*[tiab] OR urine-culture*[tiab] OR reagent-strip*[tiab] OR dipstick[tiab] OR test-strip*[tiab] |
| **Concept 3: Children 0-18y** |
| "Child"[Mesh] OR"Adolescent"[Mesh] OR "Infant"[Mesh] OR "Minors"[Mesh] OR baby[tiab] OR babies[tiab] OR newborn*[tiab] OR neonat*[tiab] OR perinatal*[tiab] OR postnatal*[tiab] OR post-natal*[tiab] OR premature*[tiab] OR preterm*[tiab] OR pre-term*[tiab] OR child*[tiab] OR schoolchild*[tiab] OR pediatric*[tiab] OR paediatric*[tiab] OR toddler*[tiab] OR infant*[tiab] OR infancy[tiab] OR preteen*[tiab] OR pre-teen*[tiab] OR prepubertal*[tiab] OR prepubescent*[tiab] OR pubescent*[tiab] OR puberty[tiab] OR preschool*[tiab] OR pre-school*[tiab] OR boy*[tiab] OR girl*[tiab] OR minor*[tiab] OR kid[tiab] OR kids[tiab] OR offspring[tiab] OR adolescen*[tiab] OR teens[tiab] OR teenager*[tiab] OR youth*[tiab] OR student*[tiab] OR underage*[tiab] OR juvenile*[tiab] OR junior*[tiab] OR puerile*[tiab] OR young*[tiab] OR ”day old”[tiab] OR “days old”[tiab] OR “month old”[tiab] OR “months old”[tiab] OR “age 1”[tiab] OR “age one”[tiab] OR “ages 1”[tiab] OR “ages one” OR 1-year-old*[tiab] OR one-year-old*[tiab] OR “1 year of age”[tiab] OR “age 2”[tiab] OR “age two”[tiab] OR “ages 2”[tiab] OR “ages two”[tiab] OR 2-year-old*[tiab] OR 2-years-old*[tiab] OR two-year-old*[tiab] OR two-years-old*[tiab] OR “2 years of age”[tiab] OR “age 3”[tiab] OR “age three”[tiab] OR “ages 3”[tiab] OR “ages three”[tiab] OR 3-year-old*[tiab] OR three-year-old*[tiab] OR 3-years-old*[tiab] OR three-years-old*[tiab] OR “3 years of age”[tiab] OR “age 4”[tiab] OR “age four”[tiab] OR “ages 4”[tiab] OR “ages four”[tiab] OR 4-year-old*[tiab] OR four-year-old*[tiab] OR 4-years-old*[tiab] OR four-years-old*[tiab] OR “4 years of age”[tiab] OR “age 5”[tiab] OR “age five”[tiab] OR “ages 5”[tiab] OR “ages five”[tiab] OR 5-year-old*[tiab] OR five-year-old*[tiab] OR 5-years-old*[tiab] OR five-years-old*[tiab] OR “5 years of age”[tiab] OR “age 6”[tiab] OR “age six”[tiab] OR “ages 6”[tiab] OR “ages six”[tiab] OR 6-year-old*[tiab] OR six-year-old*[tiab] OR 6-years-old*[tiab] OR six-years-old*[tiab] OR “6 years of age”[tiab] OR “age 7”[tiab] OR “age seven”[tiab] OR “ages 7”[tiab] OR “ages seven”[tiab] OR 7-year-old*[tiab] OR seven-year-old*[tiab] OR 7-years-old*[tiab] OR seven-years-old*[tiab] OR “7 years of age”[tiab] OR “age 8”[tiab] OR “age eight”[tiab] OR “ages 8”[tiab] OR “ages eight”[tiab] OR 8-year-old*[tiab] OR eight-year-old*[tiab] OR 8-years-old*[tiab] OR eight-years-old*[tiab] OR “8 years of age”[tiab] OR “age 9”[tiab] OR “age nine”[tiab] OR “ages 9”[tiab] OR “ages nine”[tiab] OR 9-year-old*[tiab] OR nine-year-old*[tiab] OR 9-years-old*[tiab] OR nine-years-old*[tiab] OR “9 years of age”[tiab] OR “age 10”[tiab] OR “age ten”[tiab] OR “ages 10”[tiab] OR “ages ten”[tiab] OR 10-year-old*[tiab] OR ten-year-old*[tiab] OR 10-years-old*[tiab] OR ten-years-old*[tiab] OR “10 years of age”[tiab] OR “age 12”[tiab] OR “age twelve”[tiab] OR “ages 12”[tiab] OR “ages twelve”[tiab] OR 12-year-old*[tiab] OR twelve-year-old*[tiab] OR 12-years-old*[tiab] OR twelve-years-old*[tiab] OR “12 years of age”[tiab] OR “age 14”[tiab] OR “age fourteen”[tiab] OR “ages 14”[tiab] OR “ages fourteen”[tiab] OR 14-year-old*[tiab] OR fourteen-year-old*[tiab] OR 14-years-old*[tiab] OR fourteen-years-old*[tiab] OR “14 years of age”[tiab] |
| **Concept 4: Outpatients, ambulatory care** |
| "Ambulatory Care"[Mesh] OR "Ambulatory Care Facilities"[Mesh] OR "Office Visits"[Mesh] OR "Outpatient Clinics, Hospital"[Mesh] OR "General Practice"[Mesh] OR "Family Practice"[Mesh] OR "General Practitioners"[Mesh] OR "Physicians, Primary Care"[Mesh] OR "Physicians, Family"[Mesh] OR "Primary Health Care"[Mesh] OR "Emergency Medical Services"[Mesh] OR "Emergency Service, Hospital"[Mesh] OR "After-Hours Care"[Mesh] OR ambulatory[tiab] OR outpatient*[tiab] OR “primary health care”[tiab] OR “primary care”[tiab] OR “primary healthcare”[tiab] OR “prehospital care”[tiab] OR “after-hours”[tiab] OR “out-of-hours”[tiab] OR office-visit*[tiab] OR clinic-visit*[tiab] OR house-visit*[tiab] OR home-visit*[tiab] OR emergency-medical-service*[tiab] OR “emergency care”[tiab] OR “emergency healthcare”[tiab] OR emergency-service*[tiab] OR “urgent care”[tiab] OR “accident and emergency”[tiab] OR emergency-department*[tiab] OR emergency-unit*[tiab] OR emergency-ward*[tiab] OR health-center*[tiab] OR health-centre*[tiab] OR “polyclinic*”[tiab] OR community-health-service*[tiab] OR “community health care”[tiab] OR “community healthcare”[tiab] OR community-health-facilit*[tiab] OR community-health-clinic*[tiab] OR walk-in-center*[tiab] OR walk-in-centre*[tiab] OR walk-in-clinic*[tiab] OR gp[tiab] OR general-practi*[tiab] OR general-physician*[tiab] OR general-doctor*[tiab] OR family-practi*[tiab] OR family-doctor*[tiab] OR family-physician*[tiab] OR emergency-doctor*[tiab] OR emergency-physician*[tiab] OR emergency-practitioner*[tiab] |
