# Supplementary material for: Diagnostic value of biomarkers for paediatric urinary tract infections in primary care: systematic review and meta-analysis
Source: BMC Fam Pract. 2021 Sep 27;22:193. doi: 10.1186/s12875-021-01530-9 (PMC8474745; doi:10.1186/s12875-021-01530-9)
Supplement: Supplementary file 3 — Additional file 3: Table S3. List of excluded studies with reasons why (full text screening) (n = 277). [file 12875_2021_1530_MOESM3_ESM.docx]

**Additional file 3. (Table S3).** List of excluded studies with reasons why (full text screening) (n=277)

**Table S3. List of excluded studies with reasons why (full text screening) (n=277)**

| **Reference** | **Reason for exclusion** |
| --- | --- |
| 1. Alghounaim M, Ostrow O, Timberlake K, Richardson SE, Koyle M, Science M. Antibiotic Prescription Practice for Pediatric Urinary Tract Infection in a Tertiary Center. Pediatr Emerg Care. 2021 Mar 1;37(3):150-154. doi: 10.1097/PEC.0000000000001780. PMID: 30829843. | Wrong population (adults, healthy children, malnourished children, ..) |
| 2. (2019), Clinical prediction rule to identify febrile infants 60 days and younger at low risk for serious bacterial infections. J Paediatr Child Health, 55: 1511-1511. <https://doi.org/10.1111/jpc.14651> | Editorial/commentary/letter/conference abstract |
| 3. Yao SHW, Ong GY, Maconochie IK, Lee KP, Chong SL. Analysis of emergency department prediction tools in evaluating febrile young infants at risk for serious infections. Emerg Med J. 2019 Dec;36(12):729-735. doi: 10.1136/emermed-2018-208210. Epub 2019 Oct 25. PMID: 31653694. | Wrong target condition (serious bacterial infection, vesical-urethral reflux (VUR), renal scarring, contamination ...) |
| 4. Williams-Smith JA, Fougère Y, Pauchard JY, Asner S, Gehri M, Crisinel PA. Epidemiology and risk factors for serious bacterial infections in children aged 0 to 36 months presenting with fever without source. Swiss Med Wkly. 2018;147:61S. | Editorial/commentary/letter/conference abstract |
| 5. Von Dohlen M, Jones J. A Clinical Prediction Rule to Identify Febrile Infants 60 Days and Younger at Low Risk for Serious Bacterial Infections: Kuppermann N, Dayan PS, Levine DA, et al. JAMA Pediatrics. 2019;173(4):342-351. J Emerg Med. 2019;57(3):421 | Editorial/commentary/letter/conference abstract |
| 6. Chaudhari PP, Monuteaux MC, Bachur RG. Should the Absence of Urinary Nitrite Influence Empiric Antibiotics for Urinary Tract Infection in Young Children? *Pediatr Emerg Care.* 2020;36(10):481-485. | Double (duplicate study or multiple publication on same data) |
| 7. Salas Gómez-Pablos P, Jimenez Morgades E, Martinez Martinez S, Blanco Suarez A, Perez Jové J. Application of a screening algorithm for urine cultures from the primary health to improve the microbiological diagnosis of urinary tract infections. *Clin Chem Lab Med.* 2019;57(4):eA55-eA56. | Editorial/commentary/letter/conference abstract |
| 8. Nomura O, Ihara T, Inoue N, Sakakibara H, Hirokoshi Y. Predicting serious bacterial infection in febrile young infants utilizing body temperature. *Pediatr Int.* 2019;61(5):449-452. | Wrong target condition (serious bacterial infection, vesical-urethral reflux (VUR), renal scarring, contamination ...) |
| 9. Morrissey L, Singh V, Science M, Ostrow O. 109 Promoting Diagnostic and Antimicrobial Stewardship with Urinary Tract Infections in the Paediatric Emergency Department: A Choosing Wisely Initiative...96th Annual Conference of the Canadian Paediatric Society, June 6-8 2019, Toronto, Canada. *Paediatrics & Child Health (1205-7088).* 2019;24:e41-e42. | Editorial/commentary/letter/conference abstract |
| 10. Mekonnen MT, Desta M, Yeshitela B, et al. The incidence of acute febrile illness among children in Butajira, South-Central Ethiopia. *Am J Trop Med Hyg.* 2019;101(5):153. | Wrong target condition (serious bacterial infection, vesical-urethral reflux (VUR), renal scarring, contamination ...) |
| 11. Martinson K, Kochar A, Smith N, Kelly A, Sutherland L. A retrospective review of the “step-by-step” approach in the management of young infants presenting to hospital with a history or presence of fever. *J Paediatr Child Health.* 2019;55:11-12. | Editorial/commentary/letter/conference abstract |
| 12. Malia L, Strumph K, Smith S, Brancato J, Johnson ST, Chicaiza H. Fast and Sensitive: Automated Point-of-Care Urine Dips. *Pediatr Emerg Care.* 2017. | Double (duplicate study or multiple publication on same data) |
| 13. Maduemem KE, Rodriguez YD, Fraser B. How Sensitive are Dipstick Urinalysis and Microscopy in Making Diagnosis of Urinary Tract Infection in Children? *Int J Prev Med.* 2019;10. | Wrong population (adults, healthy children, malnourished children, ..) |
| 14. Arienzo A, Cellitti V, Ferrante V, Losito F, Stalio O, Murgia L, Marino R, Cristofano F, Orrù M, Visca P, Di Somma S, Silvestri L, Ziparo V, Antonini G. A new point-of-care test for the rapid detection of urinary tract infections. Eur J Clin Microbiol Infect Dis. 2020 Feb;39(2):325-332. doi: | Wrong population (adults, healthy children, malnourished children, ..) |
| 15. Leger L, Juvet P, Pauchard JY. Urinary dipstick: Diagnostic efficiency in infants aged less of 3 months old with upper urinary tract infection. *Swiss Med Wkly.* 2018;147:59S. | Editorial/commentary/letter/conference abstract |
| 16. Kuppermann N, Dayan PS, Levine DA, et al. A Clinical Prediction Rule to Identify Febrile Infants 60 Days and Younger at Low Risk for Serious Bacterial Infections. *Jama Pediatrics.* 2019;173(4):342-351. | Double (duplicate study or multiple publication on same data) |
| 17. Kim JH, Lee JY, Cho HR, Lee JS, Ryu JM, Lee J. High Concentration of C-Reactive Protein Is Associated With Serious Bacterial Infection in Previously Healthy Children Aged 3 to 36 Months With Fever and Extreme Leukocytosis. *Pediatr Emerg Care.* 2019;35(5):347-352. | Wrong target condition (serious bacterial infection, vesical-urethral reflux (VUR), renal scarring, contamination ...) |
| 18. Kim H, Kim HR, Kim TH, Lee MK. Age-Specific Cutoffs of the Sysmex UF-1000i Automated Urine Analyzer for Rapid Screening of Urinary Tract Infections in Outpatients. *Ann Lab Med.* 2019;39(3):322-326. | Necessary data not reported (no 2x2 table extraction possible, data not reported for UTI separately,…) |
| 19. Jprn U. The Role of Presepsin and Other Markers in Early Diagnosis and Evaluation of Treatment of Pediatric Bacterial Infections. *http://wwwwhoint/trialsearch/Trial2aspx?TrialID=JPRN-UMIN000012539.* 2013. | Editorial/commentary/letter/conference abstract |
| 20. Jacob R, John-Denny B, Donaldson K, White B, Outhred A, McCaskill ME. Educational intervention does not reduce clean catch urine contamination rates in children presenting to the emergency department. Do we need to consider other collection methods? *J Paediatr Child Health.* 2019;55:16-17. | Wrong target condition (serious bacterial infection, vesical-urethral reflux (VUR), renal scarring, contamination ...) |
| 21. Han S, Choi SW, Cho YS. Diagnostic markers of serious bacterial infections in infants aged 29 to 90 days. *Signa Vitae.* 2019;15(1):22-26. | Wrong target condition (serious bacterial infection, vesical-urethral reflux (VUR), renal scarring, contamination ...) |
| 22. Guarino S, Capalbo D, Scalzone E, et al. Pilot study showed that poor feeding, especially with leucocyturia, increased the odds of non-febrile urinary tract infections in children who were not toilet trained. *Acta Paediatr.* | Wrong population (adults, healthy children, malnourished children, ..) |
| 23. Gonzalez M, Salmon A, Garcia S, et al. Prevalence of urinary tract infection in infants with high fever in the emergency department. *Anales De Pediatria.* 2019;91(6):386-393. | Necessary data not reported (no 2x2 table extraction possible, data not reported for UTI separately,…) |
| 24. Gomez B, Diaz H, Carro A, Benito J, Mintegi S. Performance of blood biomarkers to rule out invasive bacterial infection in febrile infants under 21 days old. *Arch Dis Child.* 2019;104(6):547-551. | Wrong target condition (serious bacterial infection, vesical-urethral reflux (VUR), renal scarring, contamination ...) |
| 25. Epalza C, Hallin M, Busson L, et al. Role of Viral Molecular Panels in Diagnosing the Etiology of Fever in Infants Younger Than 3 Months. *Clin Pediatr (Phila).* 2020;59(1):45-52. | Wrong target condition (serious bacterial infection, vesical-urethral reflux (VUR), renal scarring, contamination ...) |
| 26. Chiu IM, Huang LC, Chen IL, Tang KS, Huang YH. Diagnostic values of C-reactive protein and complete blood cell to identify invasive bacterial infection in young febrile infants. *Pediatr Neonatol.* 2019;60(2):197-200. | Wrong target condition (serious bacterial infection, vesical-urethral reflux (VUR), renal scarring, contamination ...) |
| 27. Bassett J, Stultz J, Hofstetter M, et al. Reducing overdiagnosis and treatment of pediatric urinary tract infections. *J Investig Med.* 2019;67(2):599-600. | Editorial/commentary/letter/conference abstract |
| 28. Alghounaim M, Ostrow O, Timberlake K, Richardson SE, Science M. Antibiotic prescription practice for pediatric urinary tract infection in a tertiary center. *Open Forum Infectious Diseases.* 2017;4:S350. | Wrong population (adults, healthy children, malnourished children, ..) |
| 29. Young Jae K. Usefulness of procalcitonin to predict serious bacterial infection in febrile pediatric patients. *Clin Chem.* 2016;62(10):S186. | Wrong target condition (serious bacterial infection, vesical-urethral reflux (VUR), renal scarring, contamination ...) |
| 30. Winkens RA, Leffers P, Trienekens TA, Stobberingh EE. The validity of urine examination for urinary tract infections in daily practice. *Fam Pract.* 1995;12(3):290-293. | Wrong population (adults, healthy children, malnourished children, ..) |
| 31. Waterfield T, Maney JA, Hanna M, Fairley D, Shields MD. Point-of-care testing for procalcitonin in identifying bacterial infections in young infants: A diagnostic accuracy study. *BMC Pediatr.* 2018;18(1). | Wrong target condition (serious bacterial infection, vesical-urethral reflux (VUR), renal scarring, contamination ...) |
| 32. Villanustre Ordonez C, Buznego Sanchez R, Rodicio Garcia M, et al. Comparative study of semiquantitative methods (leukocytes, nitrite test and uricult) with urine culture for the diagnosis of urinary tract infection during infancy. *An Esp Pediatr.* 1994;41(5):325-328. | Full text not found |
| 33. Velasco R, Gomez B, Hernandez-Bou S, et al. Validation of a predictive model for identifying febrile young infants with altered urinalysis at low risk of invasive bacterial infection. *Eur J Clin Microbiol Infect Dis.* 2017;36(2):281-284. | Double (duplicate study or multiple publication on same data) |
| 34. Velasco R, Benito H, Mozun R, Trujillo JE, Merino PA, Mintegi S. Febrile young infants with altered urinalysis at low risk for invasive bacterial infection. a Spanish Pediatric Emergency Research Network's Study. *Pediatr Infect Dis J.* 2015;34(1):17-21. | Double (duplicate study or multiple publication on same data) |
| 35. Velasco R, Benito H, Mozun R, Trujillo JE, Merino P. Febrile young infants with altered urinalysis at low risk for invasive bacterial infection. A Spanish pediatric emergency research network's (RISeuP-SPERG) study. *Eur J Emerg Med.* 2014;21(6):460-461. | Editorial/commentary/letter/conference abstract |
| 36. Ünal B, Delibaş A, Kuyucu N. Comparison of Different Urinalysis Techniques in the Diagnosis of Urinary Tract Infection Among Febrile Children Without an Apparent Origin of Fever. *Journal of Pediatric Infection / Cocuk Enfeksiyon Dergisi.* 2011;5(2):47-53. | Full text not found |
| 37. Thabet L, Siala E, Kaabachi O, Ben Hamida A, Kechrid A. The role of rapid tests in the screening of childhood urinary tract infection. *Tunis Med.* 2002;80(6):338-340. | Full text not found |
| 38. Taşar MA, Demir H, Atay G, Arikan Fİ, Dallar YB. The value of C-reactive protein, procalcitonin, interleukin-6 levels to predict urinary tract infection in children with fever without a focus. *Cocuk Enfeksiyon Dergisi.* 2014;8(4):165-170. | Necessary data not reported (no 2x2 table extraction possible, data not reported for UTI separately,…) |
| 39. Stansfeld JM. Urinary tract infections in children: diagnosis and treatment in general practice. *Practitioner.* 1977;218(1303):59-64. | Full text not found |
| 40. Shaikh N, Shope TR, Hoberman A, Vigliotti A, Kurs-Lasky M, Martin JM. Association Between Uropathogen and Pyuria. *Pediatrics.* 2016;138(1). | Wrong outcomes |
| 41. Scholten HG. Urological aspects of the diagnosis of pyelonephritis in infants. *Maandschr Kindergeneeskd.* 1965;33(8):269-285. | Full text not found |
| 42. Rodríguez Cervilla J, Alonso Alonso C, Fraga Bermúdez JM, et al. Urinary tract infection in children: Clinical and analytical prospective study for differential diagnosis in children with suspicion of an infectious disease. *Rev Esp Pediatr.* 2001;57(338):144-152. | Wrong outcomes |
| 43. Rodríguez Caballero AM, Novoa Vázquez P, Pérez Ruiz A, Carmona Pérez A, Cano Fernández J, Sánchez Bayle M. Assessment of leukocyturia in the diagnosis of urinary tract infections. *Rev Esp Pediatr.* 2001;57(340):305-308. | Full text not found |
| 44. Park K, Lee HE, Baek MK, Choi H. The application of urinary biomarkers to the diagnosis of febrile urinary tract infection and the prediction of subsequent scarring in young children less than 4 years old. *J Urol.* 2013;189(4):e268. | Editorial/commentary/letter/conference abstract |
| 45. Nys S, Bartelds AIM, Donker GA, Stobberingh EE. Urinary tract infections in a paediatric general practice population in the Netherlands: diagnostic performances and antimicrobial susceptibility of the isolated uropathogens. *Eur J Public Health.* 2007;17:180-180. | Full text not found |
| 46. Novak R, Powell K, Christopher N. Optimal diagnostic testing for urinary tract infection in young children. *Pediatr Dev Pathol.* 2004;7(3):226-230. | Full text not found |
| 47. Nosrati A, Ben Tov A, Reif S. Diagnostic markers of serious bacterial infections in febrile infants younger than 90 days old. *Pediatr Int.* 2014;56(1):47-52. | Wrong target condition (serious bacterial infection, vesical-urethral reflux (VUR), renal scarring, contamination ...) |
| 48. Nigrovic LE, Mahajan PV, Blumberg SM, et al. The Yale Observation Scale Score and the Risk of Serious Bacterial Infections in Febrile Infants. *Pediatrics.* 2017;140(1). | Wrong target condition (serious bacterial infection, vesical-urethral reflux (VUR), renal scarring, contamination ...) |
| 49. Morris CM, Tefuarani N, Ripa P, Laki R, Vince JD. Urinary tract infection in infants and young children presenting with fever without a focus in Port Moresby. *P N G Med J.* 2007;50(3-4):145-151. | Full text not found |
| 50. Mena Castro E, Vasquez DM, Chestaro L, De Luna E, Guzman M. Urinary tract infections in children. Arch Domin Pediatr. 1992;28(1):3-7. | Full text not found |
| 51. McCormick MC. The recognition of urinary tract infections in office-based pediatric practice. Need for a systematic approach to the use of urine cultures in ambulatory care. *Clin Pediatr (Phila).* 1978;17(9):713-717. | Wrong study outcome |
| 52. Maniaci V, Dauber A, Weiss S, Nylen E, Becker KL, Bachur R. Procalcitonin in young febrile infants for the detection of serious bacterial infections. *Pediatrics.* 2008;122(4):701-710. | Wrong target condition (serious bacterial infection, vesical-urethral reflux (VUR), renal scarring, contamination ...) |
| 53. Mahajan P, Kuppermann N, Mejias A, et al. Association of RNA Biosignatures With Bacterial Infections in Febrile Infants Aged 60 Days or Younger. *JAMA.* 2016;316(8):846-857. | Necessary data not reported (no 2x2 table extraction possible, data not reported for UTI separately,…) |
| 54. LeanosMiranda A, ContrerasHernandez I, Camacho R, VillagomezSalcedo E, CervantesGorayeb I. Diagnostic performance of some urine assays in urinary tract infection. *Revista De Investigacion Clinica-Clinical and Translational Investigation.* 1996;48(2):117-123. | Full text not found |
| 55. Leaños-Miranda A, Contreras-Hernández I, Camacho R, Villagómez-Salcedo E, Cervantes-Gorayeb I. Diagnostic yield of various urine tests in urinary tract infections. *Revista de investigación clínica; organo del Hospital de Enfermedades de la Nutrición.* 1996;48(2):117-123. | Full text not found |
| 56. Lam SK, Ha PK, Fung HT. Diagnosing urinary tract infection in young children ( <2 years old) in an accident and emergency department in Hong Kong. *Hong Kong Journal of Emergency Medicine.* 2010;17(4):420. | Editorial/commentary/letter/conference abstract |
| 57. Lai SW, Ng KC. Retrospective analysis of inflammatory parameters in acute pyelonephritis. *Scand J Urol Nephrol.* 2003;37(3):250-252. | Full text not found |
| 58. Kramer MS, Tange SM, Mills EL, Ciampi A, Bernstein ML, Drummond KN. Role of the complete blood count in detecting occult focal bacterial infection in the young febrile child. *J Clin Epidemiol.* 1993;46(4):349-357. | Necessary data not reported (no 2x2 table extraction possible, data not reported for UTI separately,…) |
| 59. Kowalsky RH, Rabiner JE, Tunik MG. Duration of fever: A predictor of UTI? *Pediatr Emerg Care.* 2009;25(10):710. | Editorial/commentary/letter/conference abstract |
| 60. Kazi BA, Buffone GJ, Revell PA, Chandramohan L, Dowlin MD, Cruz AT. Performance characteristics of urinalyses for the diagnosis of pediatric urinary tract infection. *Am J Emerg Med.* 2013;31(9):1405-1407. | Necessary data not reported (no 2x2 table extraction possible, data not reported for UTI separately,…) |
| 61. Kazi BA, Buffone GJ, Revell PA, Dowlin MD, Cruz AT, Chandramohan L. Performance of point-of-care versus laboratory urinalysis in the pediatric Emergency Department. *Pediatr Emerg Care.* 2013;29(10):1139-1140. | Wrong study outcome |
| 62. Kal'tianis PA, Bachiulis VP. Simultaneous microscopic and bacterioscopic examination of children's urine in the polyclinic. *Pediatriia.* 1985(6):36-38. | Full text not found |
| 63. Jones JR, Carstaris KL, Tanen DA. Urinalysis is not reliable to detect a urinary tract infection (UTI) in febrile infants presenting to the emergency department. *Ann Emerg Med.* 2006;48(4):S61-S61. | Editorial/commentary/letter/conference abstract |
| 64. Jeena PM, Coovadia HM, Adhikari MA. Bacteriuria in children attending a primary health care clinic: a prospective study of catheter stream urine samples. *Ann Trop Paediatr.* 1996;16(4):293-298. | Full text not found |
| 65. Irwin AD, Barton T, Grant A, Williams R, Carrol ED. SepsiTest™ molecular diagnosis of bacteraemia in febrile paediatric patients. *Clin Microbiol Infect.* 2012;18:505. | Wrong target condition (serious bacterial infection, vesical-urethral reflux (VUR), renal scarring, contamination ...) |
| 66 Hsiao AL, Chen L, Baker MD. Incidence and predictors of serious bacterial infections among 57-to 180-day-old infants. *Pediatrics.* 2006;117(5):1695-1701. | Wrong target condition (serious bacterial infection, vesical-urethral reflux (VUR), renal scarring, contamination ...) |
| 67. Birnie K, Hay AD, Wootton M, et al. Results from multivariable logistic regression models examining associations of symptoms, signs and urine dipstick tests with separate routine health service and research laboratory results. *Figshare.* 2017. | Double (duplicate study or multiple publication on same data) |
| 68. Hewstone AS. Laboratory diagnosis of urinary tract infection in children. *Aust Fam Physician.* 1976;5(10):1353-1356. | Full text not found |
| 69. Hernández-Bou S, Trenchs Sainz De La Maza V, Alarcón Gamarra M, Camacho Díaz JA, Gené Giralt A, Luaces Cubells C. Etiology and clinical course of urinary tract infections in infants less than 3 months-old. *Enferm Infecc Microbiol Clin.* 2015;33(8):516-520. | Full text not found |
| 70. Haimovici M, Felea D, Iordache C. Diagnosis of infantile urinary tract infections in ambulatory practice. *Revista medico-chirurgicala a Societaţii de Medici ş̧i Naturaliş̧ti din Iaş̧i.* 1982;86(4):669-671. | Full text not found |
| 71. Golabek B, Slowik M, Ploska-Urbanek B, Paruszkiewicz G, Drejewicz H. Defects of the low urinary tract as a cause of recurrent urinary tract infection in girls. *Polski merkuriusz lekarski : organ Polskiego Towarzystwa Lekarskiego.* 2002;12(70):265-268. | Wrong target condition (serious bacterial infection, vesical-urethral reflux (VUR), renal scarring, contamination ...) |
| 72. Garcia C, Gonzalez J, Arruebarrena D, et al. Dipstick in pediatric nephrology outpatients. *Nefrologia.* 1997;17(3):250-256. | Full text not found |
| 73. Garcia C, Gonzalez J, Arruebarrena D, et al. Dipstick in pediatric nephrology outpatients. *Nefrologia.* 1997;17(3):250-256. | Double (duplicate study or multiple publication on same data) |
| 74. Galetto-Lacour A, Leroy S, Bressan S, et al. Derivation of a decision algorithm to predict acute pyelonephritis in febrile children without source. *Swiss Med Wkly.* 2013;143:47S. | Editorial/commentary/letter/conference abstract |
| 75. De S, Williams GJ, Hayen A, et al. Value of white cell count in predicting serious bacterial infection in febrile children under 5 years of age. *Arch Dis Child.* 2014;99(6):493-499. | Wrong target condition (serious bacterial infection, vesical-urethral reflux (VUR), renal scarring, contamination ...) |
| 76. De S, Williams GJ, Hayen A, et al. Value of white cell count in predicting serious bacterial infection in febrile children under 5 years of age. *Postgrad Med J.* 2015;91(1073):138-144. | Double (duplicate study or multiple publication on same data) |
| 77. Dayan PS, Bennett J, Best R, et al. Test characteristics of the urine Gram stain in infants <= 60 days of age with fever. *Pediatr Emerg Care.* 2002;18(1):12-14. | Double (duplicate study or multiple publication on same data) |
| 78. Cusins PJ. Urinary tract infections in general practice. The value of suprapubic bladder aspiration with immediate culture plating. *S Afr Med J.* 1973;47(37):1707-1710. | Wrong target condition (serious bacterial infection, vesical-urethral reflux (VUR), renal scarring, contamination ...) |
| 79. Cuello García CA, Tamez Gómez L, Valdez Ceballos J. Total white blood cell count, erythrosedimentation rate and C-reactive protein for the detection of serious bacterial infections in 0- to 90-day-old infants with fever without a source. *Anales de Pediatria.* 2008;68(2):103-109. | Wrong target condition (serious bacterial infection, vesical-urethral reflux (VUR), renal scarring, contamination ...) |
| 80. Craver RD, Abermanis JG. Dipstick only urinalysis screen for the pediatric emergency room. *Pediatr Nephrol.* 1997;11(3):331-333. | Wrong population (adults, healthy children, malnourished children, ..) |
| 81. Crain EF, Gershel JC. Urinary tract infections in febrile infants younger than 8 weeks of age. *Pediatrics.* 1990;86(3):363-367. | Necessary data not reported (no 2x2 table extraction possible, data not reported for UTI separately,…) |
| 82. Craig JC, Irwig LM, Knight JF, Sureshkumar P, Roy LP. Symptomatic urinary tract infection in preschool Australian children. *J Paediatr Child Health.* 1998;34(2):154-159. | Necessary data not reported (no 2x2 table extraction possible, data not reported for UTI separately,…) |
| 83. Chiu CH, Lin TY, Bullard MJ. Identification of febrile neonates unlikely to have bacterial infections. *Pediatr Infect Dis J.* 1997;16(1):59-63. | Wrong target condition (serious bacterial infection, vesical-urethral reflux (VUR), renal scarring, contamination ...) |
| 84. Chen L. Urine leukocyte esterase as a predictor of urinary tract infections in febrile infants in the emergency department. *Pediatr Res.* 2004;55(4):116A-116A. | Full text not found |
| 85. Cheek J, Teo S, Craig S. Urine clean catch-time to stop the flow? *EMA - Emergency Medicine Australasia.* 2015;27:31-32. | Editorial/commentary/letter/conference abstract |
| 86. Chaudhari PP, Monuteaux MC, Bachur RG. Urine Concentration and Pyuria for Identifying UTI in Infants. *Pediatrics.* 2016;138(5). | Double (duplicate study or multiple publication on same data) |
| 87. Cabedo García VR, Novoa Gómez C, Tirado Balaguer MD, Rodríguez Morquecho N, Rodríguez Bailo MT, Solá Sandtner A. Is the technique used to collect urine important in avoiding contamination of samples? *Atencion primaria / Sociedad Espanola de Medicina de Familia y Comunitaria.* 2004;33(3):140‐144. | Wrong study outcome |
| 88. Buzayan MM, Tobgi RS. Comparison of urine culture, microscopy and nitrite dipstick tests in the detection of urinary tract infection. *Journal of the Bahrain Medical Society.* 2008;20(3):124-127. | Full text not found |
| 89. Butler CC, Sterne JA, Lawton M, et al. Nappy pad urine samples for investigation and treatment of UTI in young children: the 'DUTY' prospective diagnostic cohort study. *Br J Gen Pract.* 2016;66(648):e516-524. | Double (duplicate study or multiple publication on same data) |
| 90. Butler CC, O'Brien K, Pickles T, et al. Childhood urinary tract infection in primary care: a prospective observational study of prevalence, diagnosis, treatment, and recovery. *Br J Gen Pract.* 2015;65(633):e217-223. | Double (duplicate study or multiple publication on same data) |
| 91. Brkic S, Mustafic S, Nuhbegovic S, Ljuca F, Gavran L. Clinical and epidemiology characteristics of urinary tract infections in childhood. *Med Arh.* 2010;64(3):135-138. | Necessary data not reported (no 2x2 table extraction possible, data not reported for UTI separately,…) |
| 92. Bonadio WA, Webster H, Wolfe A, Gorecki D. Correlating infectious outcome with clinical parameters of 1130 consecutive febrile infants aged zero to eight weeks. *Pediatr Emerg Care.* 1993;9(2):84-86. | Wrong study design (case-control study, epidemiologic study, qualitative study, merely prognostic outcome, sample size below 50 patients) |
| 93. Blom M, Sorensen TL, Espersen F, Frimodt-Moller N. Validation of FLEXICULT (TM) SSI-urinary kit for use in the primary health care setting. *Scand J Infect Dis.* 2002;34(6):430-435. | Wrong population (adults, healthy children, malnourished children, ..) |
| 94. Birnie K, Hay AD, Wootton M, et al. Characteristics of children and urine samples collected via clean catch or nappy pads, for the 4808 children with both a routine health service laboratory and research laboratory result. *Figshare.* 2017. | Double (duplicate study or multiple publication on same data) |
| 95. Birnie K, Hay AD, Wootton M, et al. Comparison of microbiological diagnosis of urinary tract infection in young children by routine health service laboratories and a research laboratory: Diagnostic cohort study. *PLoS One.* 2017;12(2):e0171113. | Double (duplicate study or multiple publication on same data) |
| 96. Berg AO, Heidrich FE, Fihn SD, et al. Establishing the cause of genitourinary symptoms in women in a family practice. Comparison of clinical examination and comprehensive microbiology. *JAMA.* 1984;251(5):620-625. | Wrong population (adults, healthy children, malnourished children, ..) |
| 97. Benigno V, Di Peri S, Bianco A, et al. Quantitative determination of urine bacteria in the diagnosis of urinary tract infections in children. *Minerva Pediatr.* 1990;42(4):143-146. | Full text not found |
| 98. Begum M, Khan MK, Hossain MT, et al. Disease Pattern among Children attending Pediatric Outpatient Department in Community Based Medical College Hospital, Bangladesh. *Mymensingh Med J.* 2017;26(4):863-867. | Full text not found |
| 99. Beetz R. Urinary tract infections in children in general practice. *Deutsche medizinische Wochenschrift (1946).* 1994;119(10):363-364. | Full text not found |
| 100. Bauchner H, Philipp B, Dashefsky B, Klein JO. Prevalence of bacteriuria in febrile children. *Pediatr Infect Dis J.* 1987;6(3):239-242. | Wrong population (adults, healthy children, malnourished children, ..) |
| 101. Banuelos-Andrio L, Espino-Hernandez M, Ruperez-Lucas M, Villar-del Campo MC, Romero-Carrasco CI, Rodriguez-Caravaca G. Usefulness of analytical parameters in the management of paediatric patients with suspicion of acute pyelonephritis. Is procalcitonin reliable? *Revista Espanola De Medicina Nuclear E Imagen Molecular.* 2017;36(1):2-6. | Wrong setting (not relevant for ambulatory care, intensive care, admitted patients) |
| 102. Bachur RG, Harper MB. Predictive model for serious bacterial infections among infants younger than 3 months of age. *Pediatrics.* 2001;108(2):311-316. | Wrong target condition (serious bacterial infection, vesical-urethral reflux (VUR), renal scarring, contamination ...) |
| 103. Azab S, Zakaria M, Raafat M, Seief H. The combination of urinary IL-6 and renal biometry as useful diagnostic tools to differentiate acute pyelonephritis from lower urinary tract infection. *International Braz J Urol.* 2016;42(4):810-816. | Wrong setting (not relevant for ambulatory care, intensive care, admitted patients) |
| 104. Arlen AM, Merriman LS, Leong T, et al. Emergency Hospital Admissions for Initial Febrile Urinary Tract Infection: Do Patient Demographics Matter? *J Emerg Med.* 2015;49(6):843-848. | Wrong study outcome |
| 105. Arlen AM, Merriman LS, Kirsch JM, et al. Early effect of American Academy of Pediatrics Urinary Tract Infection Guidelines on radiographic imaging and diagnosis of vesicoureteral reflux in the emergency room setting. *J Urol.* 2015;193(5 Suppl):1760-1765. | Wrong study outcome |
| 106. Alnader MF, Dasoky HA, Al-hamiedeen N. Is C-reactive protein a valuable marker for severe bacterial infection? *Rawal Medical Journal.* 2012;37(2):152-154. | Wrong target condition (serious bacterial infection, vesical-urethral reflux (VUR), renal scarring, contamination ...) |
| 107. Bonadio WA, Smith DS, Sabnis S. The clinical characteristics and infectious outcomes of febrile infants aged 8 to 12 weeks. *Clin Pediatr (Phila).* 1994;33(2):95-99. | Wrong target condition (serious bacterial infection, vesical-urethral reflux (VUR), renal scarring, contamination ...) |
| 108. Conejo MC, Perez MJ, Palomares JC. Evaluation of a new method (Filtrachek-UTI) for the detection of bacteriuria. *Enferm Infecc Microbiol Clin.* 1989;7(7):354-357. | Wrong setting (not relevant for ambulatory care, intensive care, admitted patients) |
| 109. Almeida LR, Fonseca CDL, Fonseca RGD, et al. Laboratory diagnosis of urinary tract infection in children collected at the emergency room of a private children hospital, São Paulo, Brazil. *Clin Chem.* 2012;58(10):A226. | Full text not found |
| 110. Braae M, Mabeck CE, Traeden JB. Diagnosis of urinary tract infection. Experiences from general practice of a dip-slide method (Inculator) for the detection of bacteriuria. *Ugeskr Laeger.* 1971;133(12):555-557. | Full text not found |
| 111. Bergus GR. Urinalysis to diagnose UTI. *J Fam Pract.* 1995;40(6):601-602. | Full text not found |
| 112. Bailey Jr BL. Urinalysis predictive of urine culture results. *J Fam Pract.* 1995;40(1):45-50. | Full text not found |
| 113. Arica V, Arica S, Tutanç M, Gücük S. Analysis of children admitted to emergency department with acute abdominal pain in Van. *Duzce Medical Journal.* 2012;14(1):14-18. | Full text not found |
| 114. Van Den Bruel A, Raes M, Aertgeerts B, Buntinx F. Serious infections in children presenting at the emergency ward in Flanders (Belgium): The diagnostic value of demographic and clinical signs. *Tijdschrift voor Geneeskunde.* 2007;63(18):881-886. | Wrong target condition (serious bacterial infection, vesical-urethral reflux (VUR), renal scarring, contamination ...) |
| 115. Rudinsky SL, Carstairs KL, Reardon JM, Simon LV, Riffenburgh RH, Tanen DA. Serious bacterial infections in febrile infants in the post-pneumococcal conjugate vaccine era. *Acad Emerg Med.* 2009;16(7):585-590. | Wrong study design (case-control study, epidemiologic study, qualitative study, merely prognostic outcome, sample size below 50 patients) |
| 116. Robson J, Lurie N, Hart JT. Ten years' experience in general practice of dip-slide urine culture in children under five years old. *J R Coll Gen Pract.* 1979;29(208):658-661. | Wrong study design (case-control study, epidemiologic study, qualitative study, merely prognostic outcome, sample size below 50 patients) |
| 117. O'Brien K, Stanton N, Edwards A, Hood K, Butler CC. Prevalence of urinary tract infection (UTI) in sequential acutely unwell children presenting in primary care: exploratory study. *Scand J Prim Health Care.* 2011;29(1):19-22. | Necessary data not reported (no 2x2 table extraction possible, data not reported for UTI separately,…) |
| 118. Nibhanipudi KV. A Study to Determine the Incidence of Urinary Tract Infections in Infants and Children Ages 4 Months to 6 Years With Febrile Diarrhea. *Glob Pediatr Health.* 2016;3:2333794x16667175. | Necessary data not reported (no 2x2 table extraction possible, data not reported for UTI separately,…) |
| 119. Naseri M, Alamdaran A. Urinary tract infection and predisposing factors in children. *Iranian Journal of Pediatrics.* 2007;17(3):263-270. | Wrong study design (case-control study, epidemiologic study, qualitative study, merely prognostic outcome, sample size below 50 patients) |
| 120. Nam SW, Kim MK, Seo WH, et al. A rapid blood ngal assay for detection of renal cortical defect in infants with febrile uti: A prospective study. *Arch Dis Child.* 2012;97:A112-A113. | Editorial/commentary/letter/conference abstract |
| 121. Moldovan DA, Baghiu MD, Balas A, Fabian-Frast ER, Boeriu C. Diagnostic accuracy of three biomarkers in identifying serious bacterial infections in children younger than 36 months with fever without source. *Revista Romana De Medicina De Laborator.* 2015;23(3):313-322. | Wrong target condition (serious bacterial infection, vesical-urethral reflux (VUR), renal scarring, contamination ...) |
| 122. Marom R, Sakran W, Antonelli J, et al. Quick identification of febrile neonates with low risk for serious bacterial infection: an observational study. *Arch Dis Child Fetal Neonatal Ed.* 2007;92(1):F15-18. | Wrong target condition (serious bacterial infection, vesical-urethral reflux (VUR), renal scarring, contamination ...) |
| 123. Mahajan P, Grzybowski M, Chen X, et al. Procalcitonin as a marker of serious bacterial infections in febrile children younger than 3 years old. *Acad Emerg Med.* 2014;21(2):171-179. | Wrong target condition (serious bacterial infection, vesical-urethral reflux (VUR), renal scarring, contamination ...) |
| 124. Leroy S, Galetto-lacour A, Bressan S, et al. Derivation of a decision algorithm to predict acute pyelonephritis in febrile children without source. *Pediatr Nephrol.* 2013;28(8):1388. | Editorial/commentary/letter/conference abstract |
| 125. Lee HN, Kwak YH, Jung JY, et al. Are the parents' statements reliable for diagnosis of serious bacterial infection among the febrile children without source? *Intensive Care Medicine Experimental.* 2018;6. | Wrong target condition (serious bacterial infection, vesical-urethral reflux (VUR), renal scarring, contamination ...) |
| 126. Kaplan RL, Harper MB, Baskin MN, Macone AB, Mandl KD. Time to detection of positive cultures in 28- to 90-day-old febrile infants. *Pediatrics.* 2000;106(6):E74. | Wrong study outcome |
| 127. James V, George S, Joseph L, Mohan J. Urinary tract infection in young children presenting as fever without focus. *Pediatr Nephrol.* 2013;28(8):1384. | Editorial/commentary/letter/conference abstract |
| 128. Herr SM, Wald ER, Pitetti RD, Choi SS. Enhanced urinalysis improves identification of febrile infants ages 60 days and younger at low risk for serious bacterial illness. Pediatrics. 2001;108(4):866-871. | Wrong target condition (serious bacterial infection, vesical-urethral reflux (VUR), renal scarring, contamination ...) |
| 129. Haddon RA, Barnett PL, Grimwood K, Hogg GG. Bacteraemia in febrile children presenting to a pae3iatric emergency department. *Med J Aust.* 1999;170(10):475-478. | Double (duplicate study or multiple publication on same data) |
| 130. Garcia FJ, Nager AL. Jaundice as an early diagnostic sign of urinary tract infection in infancy. *Pediatrics.* 2002;109(5):846-851. | Wrong population (adults, healthy children, malnourished children, ..) |
| 131. Factor SH, Schillinger JA, Kalter HD, et al. Diagnosis and management of febrile children using the WHO/UNICEF guidelines for IMCI in Dhaka, Bangladesh. *Bull World Health Organ.* 2001;79(12):1096-1105. | Wrong study outcome |
| 132. Etoubleau C, Reveret M, Brouet D, et al. Moving from bag to catheter for urine collection in non-toilet-trained children suspected of having urinary tract infection: a paired comparison of urine cultures. *J Pediatr.* 2009;154(6):803-806. | Wrong study outcome |
| 133. Dubrovsky AS, Foster BJ, Jednak R, Mok E, McGillivray D. Visibility of the urethral meatus and risk of urinary tract infections in uncircumcised boys. *CMAJ.* 2012;184(15):E796-803. | Wrong index tests |
| 134. de Vos-Kerkhof E, Nijman RG, Vergouwe Y, et al. Impact of a clinical decision model for febrile children at risk for serious bacterial infections at the emergency department: a randomized controlled trial. *PLoS One.* 2015;10(5):e0127620. | Wrong study design (case-control study, epidemiologic study, qualitative study, merely prognostic outcome, sample size below 50 patients) |
| 135. De Rosa R, Grosso S, Avolio M, Modolo M, Stano P, Camporese A. Screening for urinary tract infection in children: Evaluation of diagnostic performance and optimal cut-off for Sysmex UF1000i flow cytometer. *Clin Microbiol Infect.* 2011;17:S524. | Editorial/commentary/letter/conference abstract |
| 136. Cunningham AM, Edwards A, Jones KV, Bourdeaux K, Willock J, Barnes R. Evaluation of a service development to increase detection of urinary tract infections in children. *J Eval Clin Pract.* 2005;11(1):73-76. | Wrong index tests |
| 137. Cruz AT, Mahajan P, Bonsu BK, et al. Accuracy of Complete Blood Cell Counts to Identify Febrile Infants 60 Days or Younger With Invasive Bacterial Infections. *Jama Pediatrics.* 2017;171(11). | Wrong target condition (serious bacterial infection, vesical-urethral reflux (VUR), renal scarring, contamination ...) |
| 138. Corman LI, Foshee WS, Kotchmar GS, Harbision RW. Simplified urinary microscopy to detect significant bacteriuria. *Pediatrics.* 1982;70(1):133-135. | Editorial/commentary/letter/conference abstract |
| 139. Ciragil P, Kurutas EB, Miraloglu M. New markers: urine xanthine oxidase and myeloperoxidase in the early detection of urinary tract infection. *Dis Markers.* 2014;2014:269362. | Wrong population (adults, healthy children, malnourished children, ..) |
| 140. Casey JR, Pichichero ME. A comparison of 2 white blood cell count devices to aid judicious antibiotic prescribing. *Clin Pediatr (Phila).* 2009;48(3):291-294. | Wrong study outcome |
| 141. Burgers R, de Jong TPVM, Visser M, Di Lorenzo C, Dijkgraaf MGW, Benninga MA. Functional Defecation Disorders in Children with Lower Urinary Tract Symptoms. *J Urol.* 2013;189(5):1886-1890. | Wrong target condition (serious bacterial infection, vesical-urethral reflux (VUR), renal scarring, contamination ...) |
| 142. Brockmann Veloso P, Ibarra GX, Silva WI, Hirsch BT. Etiology of acute fever without source in infants consulting at an emergency department. *Rev Chilena Infectol.* 2007;24(1):33-39. | Wrong target condition (serious bacterial infection, vesical-urethral reflux (VUR), renal scarring, contamination ...) |
| 143. Bleeker SE, Moons KG, Derksen-Lubsen G, Grobbee DE, Moll HA. Predicting serious bacterial infection in young children with fever without apparent source. *Acta Paediatr.* 2001;90(11):1226-1232. | Wrong target condition (serious bacterial infection, vesical-urethral reflux (VUR), renal scarring, contamination ...) |
| 144. Bleeker SE, Derksen-Lubsen G, Grobbee DE, Donders AR, Moons KG, Moll HA. Validating and updating a prediction rule for serious bacterial infection in patients with fever without source. *Acta Paediatr.* 2007;96(1):100-104. | Wrong target condition (serious bacterial infection, vesical-urethral reflux (VUR), renal scarring, contamination ...) |
| 145. Bin Salleeh H, McGillivray D, Martin M, Patel H. Duration of fever affects the likelihood of a positive bag urinalysis or catheter culture in young children. J Pediatr. 2010;156(4):629-633. | Wrong reference standard |
| 146. Bicer S. Analysis of children with acute abdominal pain and other acute abdominal symptoms in the pediatric emergency department. *Medical Journal of Bakirkoy.* 2009;5(3):96-102. | Wrong target condition (serious bacterial infection, vesical-urethral reflux (VUR), renal scarring, contamination ...) |
| 147. Barongo AK. Urinary tract infection: Prevalence, pathogens and antimicrobial susceptibility pattern among febrile children at Mwananyamala hospital, Tanzania. *Am J Trop Med Hyg.* 2013;89(5):354. | Editorial/commentary/letter/conference abstract |
| 148. André M, Vernby A, Odenholt I, et al. General practitioners prescribed less antibiotics but used the CRP test more. Diagnosis-prescription studies in 2000-2005. *Lakartidningen.* 2008;105(41):2851-2854. | Full text not found |
| 149. Yan JH, Cai XY, Huang YH. The clinical value of plasma hepcidin levels in predicting bacterial infections in febrile children. *Pediatr Neonatol.* 2018. | Wrong target condition (serious bacterial infection, vesical-urethral reflux (VUR), renal scarring, contamination ...) |
| 150. Putri AU, Rina O, Rosmayanti, Ramayati R, Rusdidjas. Comparison of urine Gram stain and urine culture to diagnose urinary tract infection in children. *Paediatr Indones.* 2013;53(2):121-124. | Wrong setting (not relevant for ambulatory care, intensive care, admitted patients) |
| 151. Huysal K, Budak YU, Karaca AU, et al. Diagnostic accuracy of uriSed automated urine microscopic sediment analyzer and dipstick parameters in predicting urine culture test results. *Biochem Med (Zagreb).* 2013;23(2):211-217. | Wrong population (adults, healthy children, malnourished children, ..) |
| 152. Velasco R, Trujillo JE, Benito H, et al. Value of blood biomarkers to identify young febrile infants diagnosed with uti at higher risk for bacteremia. Initial results. *Arch Dis Child.* 2012;97:A418-A419. | Editorial/commentary/letter/conference abstract |
| 153. Pratt A, Attia MW. Duration of fever and markers of serious bacterial infection. *Pediatr Res.* 2004;55(4):115A-115A. | Wrong target condition (serious bacterial infection, vesical-urethral reflux (VUR), renal scarring, contamination ...) |
| 154. Silverberg DS, Allard MJ, Ulan RA. City wide screening for urinary abnormalities in schoolgirls. *Can Med Assoc J.* 1973;109(10):981-985. | Wrong population (adults, healthy children, malnourished children, ..) |
| 155. Silverberg DS. City wide screening for urinary abnormalities in schoolboys. *Can Med Assoc J.* 1974;111(5):410-412. | Wrong population (adults, healthy children, malnourished children, ..) |
| 156. Pulliam PN, Attia MW, Cronan KM. C-reactive protein in febrile children 1 to 36 months of age with clinically undetectable serious bacterial infection. *Pediatrics.* 2001;108(6):1275-1279. | Wrong target condition (serious bacterial infection, vesical-urethral reflux (VUR), renal scarring, contamination ...) |
| 157. Rudensky B, Sirota G, Erlichman M, Yinnon AM, Schlesinger Y. Neutrophil CD64 expression as a diagnostic marker of bacterial infection in febrile children presenting to a hospital emergency department. *Pediatr Emerg Care.* 2008;24(11):745-748. | Wrong study design (case-control study, epidemiologic study, qualitative study, merely prognostic outcome, sample size below 50 patients) |
| 158. Waldron CA, Thomas-Jones E, Pickles T, et al. Recruitment to diagnosis of urinary tract infections in young children (DUTY) study: An evaluation of the successful methods used in a primary care, prospective cohort study. *Trials.* 2013;14:165DUMMY. | Editorial/commentary/letter/conference abstract |
| 159. Vujevic M, Benzon B, Markic J. New prediction model for diagnosis of bacterial infection in febrile infants younger than 90 days. *Turk J Pediatr.* 2017;59(3):261-268. | Wrong target condition (serious bacterial infection, vesical-urethral reflux (VUR), renal scarring, contamination ...) |
| 160. Shallcross L, Gaskell K, Fox-Lewis A, Bergstrom M, Noursadeghi M. Mismatch between suspected pyelonephritis and microbiological diagnosis: a cohort study from a UK teaching hospital. *J Hosp Infect.* 2018;98(2):219-222. | Wrong population (adults, healthy children, malnourished children, ..) |
| 161. Stalenhoef JE, van Nieuwkoop C, Wilson DC, et al. Biomarker guided triage can reduce hospitalization rate in community acquired febrile urinary tract infection. *J Infect.* 2018;77(1):18-24. | Wrong population (adults, healthy children, malnourished children, ..) |
| 162. Haddon RA, Barnett PL, Grimwood K, Hogg GG. Bacteraemia in febrile children presenting to a pae3iatric emergency department. *Med J Aust.* 1999;170(10):475-478. | Wrong target condition (serious bacterial infection, vesical-urethral reflux (VUR), renal scarring, contamination ...) |
| 163. Fabiani Hurtado NR, Mejía Salas H. Fiebre sin foco en niños menores de 36 meses tratados en el servicio de emergencias del Hospital del Niño "Dr. Ovidio Aliaga Uría". *Revista de la Sociedad Boliviana de Pediatría.* 2014;53(1):3-7. | Wrong target condition (serious bacterial infection, vesical-urethral reflux (VUR), renal scarring, contamination ...) |
| 164. Çalışkan E, Şahin İ, Öztürk CE, Yavuz MT, Ankaralı H, Türkmen-Albayrak H. Üriner Sistem İnfeksiyonlarının Tanısında Kullanılan Mikrobiyolojik Yöntemlerin Karşılaştırılması. *Klimik Journal / Klimik Dergisi.* 2013;25(4):9-12. | Wrong population (adults, healthy children, malnourished children, ..) |
| 165. Chopard M, Howell JM, Place RC, Nam MH, Najafi A, Druckenbrod G. Accuracy of automatic urinalysis in the screening of febrile infants for pediatric urinary tract infection. *Acad Emerg Med.* 2011;18(5):S73. | Editorial/commentary/letter/conference abstract |
| 166. Galetto-Lacour A, Zamora SA, Gervaix A. Bedside Procalcitonin and C-Reactive Protein Tests in Children With Fever Without Localizing Signs of Infection Seen in a Referral Center. *Pediatrics.* 2003;112(5):1054-1060. | Wrong target condition (serious bacterial infection, vesical-urethral reflux (VUR), renal scarring, contamination ...) |
| 167. Manzano S, Bailey B, Gervaix A, Cousineau J, Delvin E, Girodias JB. Markers for bacterial infection in children with fever without source. *Arch Dis Child.* 2011;96(5):440-446. | Wrong target condition (serious bacterial infection, vesical-urethral reflux (VUR), renal scarring, contamination ...) |
| 168. Markic J, Jeroncic A, Polancec D, et al. CD15s is a potential biomarker of serious bacterial infection in infants admitted to hospital. *Eur J Pediatr.* 2013;172(10):1363-1369. | Wrong target condition (serious bacterial infection, vesical-urethral reflux (VUR), renal scarring, contamination ...) |
| 169. Fitzgerald AL, Okafor I, McNamara R, Deiratany S. Point of care urine microscopy to predict urinary tract infection in a paediatric emergency Department. *Arch Dis Child.* 2014;99:A10. | Editorial/commentary/letter/conference abstract |
| 170. Kadish HA, Loveridge B, Tobey J, Bolte RG, Corneli HM. Applying outpatient protocols in febrile infants 1-28 days of age: can the threshold be lowered? *Clin Pediatr (Phila).* 2000;39(2):81-88. | Wrong target condition (serious bacterial infection, vesical-urethral reflux (VUR), renal scarring, contamination ...) |
| 171. Moldovan DA, Baghiu MD, Balas A, Truta ST. The Value of the "Lab-Score" Method in Identifying Febrile Infants at Risk for Serious Bacterial Infections. *J Crit Care Med (Targu Mures).* 2015;1(1):11-17. | Wrong target condition (serious bacterial infection, vesical-urethral reflux (VUR), renal scarring, contamination ...) |
| 172. Nijman RG, Moll HA, Smit FJ, et al. C-reactive protein, procalcitonin and the lab-score for detecting serious bacterial infections in febrile children at the emergency department: A prospective observational study. *Pediatr Infect Dis J.* 2014;33(11):e273-e279. | Wrong target condition (serious bacterial infection, vesical-urethral reflux (VUR), renal scarring, contamination ...) |
| 173. Ferrera PC, Bartfield JM, Snyder HS, Ferrera PC, Bartfield JM, Snyder HS. Neonatal fever: utility of the Rochester criteria in determining low risk for serious bacterial infections. *Am J Emerg Med.* 1997;15(3):299-302. | Wrong target condition (serious bacterial infection, vesical-urethral reflux (VUR), renal scarring, contamination ...) |
| 174. Frohna JG. Diagnostic model appears to be more effective than clinical judgment in detecting serious bacterial infection in young febrile children. *J Pediatr.* 2010;157(5):862-863. | Editorial/commentary/letter/conference abstract |
| 175. Milcent K, Faesch S, Guen CGL, et al. Use of Procalcitonin Assays to Predict Serious Bacterial Infection in Young Febrile Infants. *JAMA Pediatrics.* 2016;170(1):62-69. | Wrong target condition (serious bacterial infection, vesical-urethral reflux (VUR), renal scarring, contamination ...) |
| 176. Nijman RG, Vergouwe Y, Moll HA, et al. Validation of the Feverkidstool and procalcitonin for detecting serious bacterial infections in febrile children. *Pediatr Res.* 2018;83(2):466-476. | Double (duplicate study or multiple publication on same data) |
| 177. Middelkoop SJ, van Pelt LJ, Kampinga GA, Ter Maaten JC, Stegeman CA. Routine tests and automated urinalysis in patients with suspected urinary tract infection at the ED. *Am J Emerg Med.* 2016;34(8):1528-1534. | Wrong population (adults, healthy children, malnourished children, ..) |
| 178. Nijman RG, Vergouwe Y, Thompson M, et al. Clinical prediction model to aid emergency doctors managing febrile children at risk of serious bacterial infections: diagnostic study. *BMJ.* 2013;346:f1706. | Wrong target condition (serious bacterial infection, vesical-urethral reflux (VUR), renal scarring, contamination ...) |
| 179. Nijman RG, Zwinkels RL, van Veen M, et al. Can urgency classification of the Manchester triage system predict serious bacterial infections in febrile children? *Arch Dis Child.* 2011;96(8):715-722. | Wrong target condition (serious bacterial infection, vesical-urethral reflux (VUR), renal scarring, contamination ...) |
| 180. Olaciregui Echenique I, Hernández U, Muñoz JA, Emparanza JI, Landa JJ. Markers that predict serious bacterial infection in infants under 3 months of age presenting with fever of unknown origin. *Arch Dis Child.* 2009;94(7):501-505. | Wrong target condition (serious bacterial infection, vesical-urethral reflux (VUR), renal scarring, contamination ...) |
| 181. Parameswaran K, Gambaratto K, Giglia L. Age-specific predictive findings on urinalysis for positive cultures in children less than 24 months with urinary tract infections. *Paediatrics and Child Health.* 2011;16:45A. | Editorial/commentary/letter/conference abstract |
| 182. Yilmaz A, Gedikbasi A, Kiyak A, et al. S100 calgranulins in urinary tract infection. *Pediatr Nephrol.* 2011;26(9):1706. | Editorial/commentary/letter/conference abstract |
| 183. Winkens R, Nelissen-Arets H, Stobberingh E. Validity of the urine dipslide under daily practice conditions. *Fam Pract.* 2003;20(4):410-412. | Wrong population (adults, healthy children, malnourished children, ..) |
| 184. Pratt A, Attia MW. Duration of fever and markers of serious bacterial infection in young febrile children. *Pediatr Int.* 2007;49(1):31-35. | Wrong target condition (serious bacterial infection, vesical-urethral reflux (VUR), renal scarring, contamination ...) |
| 185. Nys S, van Merode T, Bartelds AI, Stobberingh EE. Urinary tract infections in general practice patients: diagnostic tests versus bacteriological culture. *J Antimicrob Chemother.* 2006;57(5):955-958. | Wrong population (adults, healthy children, malnourished children, ..) |
| 186. Moldovan D, Baghiu D, Boeriu C, Balas A, Kovari A. Assessment and comparison of a lab-score and a clinical prediction model for detecting serious bacterial infections in febrile young children. *Arch Dis Child.* 2014;99:A335. | Editorial/commentary/letter/conference abstract |
| 187. McIsaac WJ, Moineddin R, Ross S. Validation of a decision aid to assist physicians in reducing unnecessary antibiotic drug use for acute cystitis. *Arch Intern Med.* 2007;167(20):2201-2206. | Wrong population (adults, healthy children, malnourished children, ..) |
| 188. McIsaac WJ, Moineddin R, Gagyor I, Mazzulli T. External validation study of a clinical decision aid to reduce unnecessary antibiotic prescriptions in women with acute cystitis. *BMC Fam Pract.* 2017;18(1):89. | Wrong population (adults, healthy children, malnourished children, ..) |
| 189. Lucas-Saez E, Ferrando-Monleon S, Marin-Serra J, et al. Predictive factors for kidney damage in febrile urinary tract infection. Usefulness of procalcitonin. *Nefrologia.* 2014;34(4):451-457. | Wrong target condition (serious bacterial infection, vesical-urethral reflux (VUR), renal scarring, contamination ...) |
| 190. Cheek JA, Craig SS, Seith RW, West A. Urine collection in young children. *EMA - Emergency Medicine Australasia.* 2015;27(4):348-350. | Editorial/commentary/letter/conference abstract |
| 191. Bressan S, Andreola B, Cattelan F, Zangardi T, Perilongo G, Da Dalt L. Predicting Severe Bacterial Infections in Well-Appearing Febrile Neonates Laboratory Markers Accuracy and Duration of Fever. *Pediatr Infect Dis J.* 2010;29(3):227-232. | Wrong target condition (serious bacterial infection, vesical-urethral reflux (VUR), renal scarring, contamination ...) |
| 192. Mahajan P, Kuppermann N, Mejias A, et al. RNA transcriptional profiling for diagnosis of serious bacterial infections (SBIs) in young febrile infants. *Acad Emerg Med.* 2012;19:S5-S6. | Editorial/commentary/letter/conference abstract |
| 193. D'Souza AJ, Shetty A, Krishnan DK. REACTIVE THROMBOCYTOSIS IN FEBRILE CHILDREN WITH SERIOUS BACTERIAL INFECTION. *Journal of Evolution of Medical and Dental Sciences-Jemds.* 2014;3(55):12537-12543. | Wrong target condition (serious bacterial infection, vesical-urethral reflux (VUR), renal scarring, contamination ...) |
| 194. Jones C, Culbreath K, Mehrotra A, Gilligan P, Shofer F. Urinalysis reflex testing in the emergency department. *Acad Emerg Med.* 2011;18(5):S197-S198. | Editorial/commentary/letter/conference abstract |
| 195. Claessens YE, Schmidt J, Batard E, et al. Can C-reactive protein, procalcitonin and mid-regional pro-atrial natriuretic peptide measurements guide choice of in-patient or out-patient care in acute pyelonephritis? Biomarkers In Sepsis (BIS) multicentre study. *Clin Microbiol Infect.* 2010;16(6):753-760. | Wrong population (adults, healthy children, malnourished children, ..) |
| 196. Isaacman DJ, Burke BL. Utility of the serum C-reactive protein for detection of occult bacterial infection in children. *Arch Pediatr Adolesc Med.* 2002;156(9):905-909. | Wrong target condition (serious bacterial infection, vesical-urethral reflux (VUR), renal scarring, contamination ...) |
| 197. Kasmire KE, Vega C, Bennett NJ, Laurich VM. Hypothermia: A Sign of Sepsis in Young Infants in the Emergency Department? *Pediatr Emerg Care.* 2018. | Wrong target condition (serious bacterial infection, vesical-urethral reflux (VUR), renal scarring, contamination ...) |
| 198. Keitel K, Kagoro F, Samaka J, et al. A novel electronic algorithm using host biomarker point-of-care tests for the management of febrile illnesses in Tanzanian children (e-POCT): A randomized, controlled non-inferiority trial. *PLoS Med.* 2017;14(10):1-29. | Wrong target condition (serious bacterial infection, vesical-urethral reflux (VUR), renal scarring, contamination ...) |
| 199. Kerr D, Holland RR, Eckhardt EM, et al. Development of a clinical prediction rule for urinary tract infection. *Ann Emerg Med.* 2016;68(4):S47. | Editorial/commentary/letter/conference abstract |
| 200. Kim JH, Lee JY, Cho HR, Lee JS, Ryu JM, Lee J. High Concentration of C-Reactive Protein Is Associated With Serious Bacterial Infection in Previously Healthy Children Aged 3 to 36 Months With Fever and Extreme Leukocytosis. *Pediatr Emerg Care.* 2017. | Wrong target condition (serious bacterial infection, vesical-urethral reflux (VUR), renal scarring, contamination ...) |
| 201. Lee IS, Park YJ, Jin MH, et al. Usefulness of the procalcitonin test in young febrile infants between 1 and 3 months of age. *Korean J Pediatr.* 2018;61(9):285-290. | Wrong target condition (serious bacterial infection, vesical-urethral reflux (VUR), renal scarring, contamination ...) |
| 202. Little P, Turner S, Rumsby K, et al. Developing clinical rules to predict urinary tract infection in primary care settings: sensitivity and specificity of near patient tests (dipsticks) and clinical scores. *Br J Gen Pract.* 2006;56(529):606-612. | Wrong population (adults, healthy children, malnourished children, ..) |
| 203. Leroy S, Galetto-Lacour A, Bressan S, Andreol B, Dadalt L, Gervaix A. Derivation of a decision algorithm to predict acute pyelonephritis in febrile children without source. *Pediatr Nephrol.* 2014;29(9):1680. | Editorial/commentary/letter/conference abstract |
| 204. Lannergard A, Friman G, Larsson A. Serum amyloid A: A novel serum marker for the detection of systemic inflammatory response in cystitis. *J Urol.* 2003;170(3):804-806. | Wrong population (adults, healthy children, malnourished children, ..) |
| 205. Gauthier M, Gouin S, Phan V, Phan J, Gravel J. Predictive ability of “smelly” urine for urinary tract infection (UTI) in 1 to 36 month old children. *Paediatrics and Child Health.* 2011;16:23A. | Editorial/commentary/letter/conference abstract |
| 206. Gauthier M, Gouin S, Phan V, Phan J, Gravel J. Predictive ability of “smelly” urine for urinary tract infection (UTI) in 1 to 36 month old children. *Paediatrics and Child Health.* 2011;16:23A. | Double (duplicate study or multiple publication on same data) |
| 207. Cubells CL. Implementation of procalcitonin in the management of febrile children. *Pediatr Infect Dis J.* 2012;31(7):793. | Editorial/commentary/letter/conference abstract |
| 208. Choi K, Chang I, Lee JC, et al. Smartphone-Based Urine Reagent Strip Test in the Emergency Department. *Telemedicine and E-Health.* 2016;22(6):534-540. | Wrong population (adults, healthy children, malnourished children, ..) |
| 209. Byington CL, Reynolds CC, Korgenski K, et al. Costs and Infant Outcomes After Implementation of a Care Process Model for Febrile Infants. *Pediatrics.* 2012;130(1):E16-E24. | Wrong study outcome |
| 210. Bonadio WA. Urine culturing technique in febrile infants. *Pediatr Emerg Care.* 1987;3(2):75-78. | Wrong index tests |
| 211. Dudley J, Butler C, Hood K, et al. The Diagnosis of Urinary Tract Infection In Young children (DUTY) study: A clinical algorithm to improve the recognition of Urinary Tract Infection (UTI) in pre-school children. *Pediatr Nephrol.* 2013;28(8):1372. | Editorial/commentary/letter/conference abstract |
| 212. Hay AD, Hood K, Sterne J, et al. The diagnosis of urinary tract infection in young children (duty) study: The development of a clinical algorithm to improve the recognition of urinary tract infection (UTI) in pre-school children presenting to primary care. *Arch Dis Child Educ Pract Ed.* 2013;98:A25. | Editorial/commentary/letter/conference abstract |
| 213. Finch RM, Finch J. Bacteriological counts of urines in general practice. *J R Coll Gen Pract.* 1970;19(93):201-210. | Wrong population (adults, healthy children, malnourished children, ..) |
| 214. Hernandez L, Diaz De Tuesta J, Sanchez J, Cisterna R. Prevalence of urinary tract infections in neonates. *Clin Microbiol Infect.* 2010;16:S713. | Editorial/commentary/letter/conference abstract |
| 215. Kool M, Elshout G, Koes BW, Bohnen AM, Berger MY. C-reactive protein level as diagnostic marker in young febrile children presenting in a general practice out-of-hours service. *J Am Board Fam Med.* 2016;29(4):460-468. | Wrong target condition (serious bacterial infection, vesical-urethral reflux (VUR), renal scarring, contamination ...) |
| 216. Bressan S, Andreola B, Zucchetta P, et al. Procalcitonin as a predictor of renal scarring in infants and young children. *Pediatr Nephrol.* 2009;24(6):1199-1204. | Double (duplicate study or multiple publication on same data) |
| 217. Brent AJ, Lakhanpaul M, Thompson M, et al. Risk score to stratify children with suspected serious bacterial infection: observational cohort study. *Arch Dis Child.* 2011;96(4):361-367. | Wrong target condition (serious bacterial infection, vesical-urethral reflux (VUR), renal scarring, contamination ...) |
| 218. Baker MD, Bell LM, Avner JR. The efficacy of routine outpatient management without antibiotics of fever in selected infants. *Pediatrics.* 1999;103(3):627-631. | Wrong target condition (serious bacterial infection, vesical-urethral reflux (VUR), renal scarring, contamination ...) |
| 219. Bachur R. Pediatric urinary tract infection. *Clin Pediatr Emerg Med.* 2004;5(1):28-36. | Editorial/commentary/letter/conference abstract |
| 220. Dubrovsky AS, Foster B, Jednak R, McGillivray D. Visibility of the urethral opening does not corelate with risk of urinary tract infection in uncircumcised boys. *Paediatrics and Child Health.* 2010;15:51A-52A. | Editorial/commentary/letter/conference abstract |
| 221. Deakin AG, Jones GR, Spencer JW, et al. A portable system for identifying urinary tract infection in primary care using a PC-based chromatic technique. *Physiol Meas.* 2014;35(5):793-805. | Wrong population (adults, healthy children, malnourished children, ..) |
| 222. Nigrovic LE, Mahajan PV, Tzimenatos L, et al. The accuracy of the yale observation scale score and unstructured clinician suspicion to identify febrile infants aged £60 days with serious bacterial infections. *Ann Emerg Med.* 2015;66(4):S86-S87. | Editorial/commentary/letter/conference abstract |
| 223. Cao Y, Cheng M, Hu C. UrineCART, a machine learning method for establishment of review rules based on UF-1000i flow cytometry and dipstick or reflectance photometer. *Clin Chem Lab Med.* 2012;50(12):2155-2161. | Wrong population (adults, healthy children, malnourished children, ..) |
| 224. Bongard E, Frimodt-Moller N, Gal M, et al. Analytic laboratory performance of a point of care urine culture kit for diagnosis and antibiotic susceptibility testing. *Eur J Clin Microbiol Infect Dis.* 2015;34(10):2111-2119. | Wrong population (adults, healthy children, malnourished children, ..) |
| 225. Baraff LJ. Management of infants and young children with fever without source. *Pediatr Ann.* 2008;37(10):673-679. | (systematic) review |
| 226. Edwards A, van der Voort J, Newcombe R, Thayer H, Verrier Jones K. A urine analysis method suitable for children's nappies. *J Clin Pathol.* 1997;50(7):569-572. | Wrong population (adults, healthy children, malnourished children, ..) |
| 227. Edwards B, White RH, Maxted H, Deverill I, White PA. Screening methods for covert bacteriuria in schoolgirls. *Br Med J.* 1975;2(5969):463-467. | Wrong population (adults, healthy children, malnourished children, ..) |
| 228. Busby J, Hay A, Butler C, et al. Cost effective use of urine sampling and dipstick testing to diagnose urinary tract infections in pre-school children presenting to primary care. *Value Health.* 2013;16(7):A630. | Editorial/commentary/letter/conference abstract |
| 229. Bigot S, Leblond P, Foucher C, Hue V, D'Herbomez M, Foulard M. Usefulness of procalcitonin for the diagnosis of acute pyelonephritis in children. *Arch Pediatr.* 2005;12(7):1075-1080. | Wrong study design (case-control study, epidemiologic study, qualitative study, merely prognostic outcome, sample size below 50 patients) |
| 230. Barrett MJ, Pollock J, Harney T, et al. The correlation between c-reactive protein and serious bacterial infection in a tertiary paediatric emergency department: An observational case-controlled cohort study. *Arch Dis Child.* 2012;97:A446-A447. | Editorial/commentary/letter/conference abstract |
| 231. Aronson PL, McCulloh RJ, Tieder JS, et al. Application of the Rochester Criteria to Identify Febrile Infants With Bacteremia and Meningitis. *Pediatr Emerg Care.* 2019;35(1):22-27. | Wrong target condition (serious bacterial infection, vesical-urethral reflux (VUR), renal scarring, contamination ...) |
| 232. Althaus T, Greer RC, Swe MMM, et al. Effect of point-of-care C-reactive protein testing on antibiotic prescription in febrile patients attending primary care in Thailand and Myanmar: an open-label, randomised, controlled trial. *The Lancet Global Health.* 2019;7(1):e119-e131. | Wrong population (adults, healthy children, malnourished children, ..) |
| 233. Almond C. Issues in paediatric triage. *Australian Emergency Nursing Journal.* 2000;3(1):12-14. | Editorial/commentary/letter/conference abstract |
| 234. Ali NS. Evaluation and management of urinary tract infection in children in general practice. *J Pak Med Assoc.* 2001;51(4):164-165. | Editorial/commentary/letter/conference abstract |
| 235. Alcalde Martín C, Centeno Malfaz F, González Armengod C, et al. Diagnostic test in emergency departments for bacterial infections in infants younger than 12 months. *Anales de Pediatria.* 2003;58(1):17-22. | Wrong study design (case-control study, epidemiologic study, qualitative study, merely prognostic outcome, sample size below 50 patients) |
| 236. Alam S, Voort JVD, Butler CC. Urine concentration should be taken into account when interpreting pyuria in infants. *Evid Based Med.* 2017;22(3):115. | Editorial/commentary/letter/conference abstract |
| 237. Al-Orifi F, McGillivray D, Tange S, Kramer MS. Urine culture from bag specimens in young children: are the risks too high? *J Pediatr.* 2000;137(2):221-226. | Wrong index tests |
| 238. Al-Majali RM. White blood cell count, absolute neutrophil count, as predictors of hidden bacterial infections in febrile children 1-18 months of age without focus. *Pakistan Journal of Medical Sciences.* 2004;20(2):97-100. | Wrong study design (case-control study, epidemiologic study, qualitative study, merely prognostic outcome, sample size below 50 patients) |
| 239. Ahmed SM, Swedlund SK. Evaluation and treatment of urinary tract infections in children. *Am Fam Physician.* 1998;57(7):1573-1580, 1583-1574. | Editorial/commentary/letter/conference abstract |
| 240. Ahmad FS. Acute assessment of infants presenting to primary care. *Br J Gen Pract.* 2002;52(477):330. | Editorial/commentary/letter/conference abstract |
| 241. Aguiar C, Abreu M, Pinto A, Oliveira J, Pereira S, Madalena C. Procalcitonin as a predictor of renal scarring and vesicoureteral reflux in pediatric acute pyelonephritis. *Pediatr Nephrol.* 2014;29(9):1788. | Editorial/commentary/letter/conference abstract |
| 242. Bahagon Y, Raveh D, Schlesinger Y, Rudensky B, Yinnon AM. Prevalence and predictive features of bacteremic urinary tract infection in emergency department patients. *Eur J Clin Microbiol Infect Dis.* 2007;26(5):349-352. | Wrong population (adults, healthy children, malnourished children, ..) |
| 243. Alper BS, Curry SH. Urinary tract infection in children. *Am Fam Physician.* 2005;72(12):2483-2488. | (systematic) review |
| 244. Alharthi AA, Taha AA, Edrees AE, Elnawawy AN, Abdelrahman AH. Screening for urine abnormalities among preschool children in western Saudi Arabia. *Saudi Med J.* 2014;35(12):1477-1481. | Wrong population (adults, healthy children, malnourished children, ..) |
| 245. Al Shammari SA, Al Quaiz JM, Chowdhury MNH. Presentation and management of urinary tract infections in primary care clinics in Riyadh, Saudi Arabia. *Med Sci Res.* 1995;23(12):807-809. | Wrong population (adults, healthy children, malnourished children, ..) |
| 246. Abdul-Massih CE, Sawaya RD, Mrad S, El Zahran T, Tamim H, Majdalani MN. Presentation and management of febrile children in atertiary care emergency department. *J Emerg Med.* 2017;53(3):433. | Editorial/commentary/letter/conference abstract |
| 247. Kasmire KE, Vega C, Bennett NJ, Laurich VM. Hypothermia: A Sign of Sepsis in Young Infants in the Emergency Department? *Pediatr Emerg Care.* 2021;37(3):e124-e128. | Wrong target condition (serious bacterial infection, vesical-urethral reflux (VUR), renal scarring, contamination ...) |
| 248. Yankova LC, Neuman MI, Wang ME, et al. Febrile Infants </=60 Days Old With Positive Urinalysis Results and Invasive Bacterial Infections. *Hosp Pediatr.* 2020;10(12):1120-1125. | Full text not found |
| 249. Hoberman A, Wald ER, Reynolds EA, Penchansky L, Charron M. Pyuria and bacteriuria in urine specimens obtained by catheter from young children with fever. *J Pediatr.* 1994;124(4):513-519. | Double (duplicate study or multiple publication on same data) |
| 250. Van den Bruel A, Aertgeerts B, Bruyninckx R, Aerts M, Buntinx F. Signs and symptoms for diagnosis of serious infections in children: a prospective study in primary care. *Br J Gen Pract.* 2007;57(540):538-546. | Wrong target condition (serious bacterial infection, vesical-urethral reflux (VUR), renal scarring, contamination ...) |
| 251. Lee HE, Kim DK, Kang HK, Park K. The diagnosis of febrile urinary tract infection in children may be facilitated by urinary biomarkers. *Pediatr Nephrol.* 2014;30(1):123-130. | Wrong study design (case-control study, epidemiologic study, qualitative study, merely prognostic outcome, sample size below 50 patients) |
| 252. Hay AD, Birnie K, Busby J, et al. The Diagnosis of Urinary Tract infection in Young children (DUTY): a diagnostic prospective observational study to derive and validate a clinical algorithm for the diagnosis of urinary tract infection in children presenting to primary care with an acute illness. *Health Technol Assess.* 2016;20(51):1-294. | Double (duplicate study or multiple publication on same data) |
| 253. Yoon SH, Shin H, Lee KH, et al. Predictive factors for bacteremia in febrile infants with urinary tract infection. *Sci Rep.* 2020;10(1). | Wrong setting (not relevant for ambulatory care, intensive care, admitted patients) |
| 254. Craig JC, Knight JF, Sureshkumar P, Mantz E, Roy LP. Effect of circumcision on incidence of urinary tract infection in preschool boys. *J Pediatr.* 1996;128(1):23-27. | Wrong study design (case-control study, epidemiologic study, qualitative study, merely prognostic outcome, sample size below 50 patients) |
| 255. Ramgopal S, Horvat CM, Yanamala N, Alpern ER. Machine Learning To Predict Serious Bacterial Infections in Young Febrile Infants. *Pediatrics.* 2020;146(3). | Wrong target condition (serious bacterial infection, vesical-urethral reflux (VUR), renal scarring, contamination ...) |
| 256. Kamyab F, Gholami M, Shaghaghi F, Bidkhori M, Kamali Z. Urine analysis with dipstick test in asymptomatic 7-year-old children. *Journal of Education and Health Promotion.* 2020;9(1). | Wrong population (adults, healthy children, malnourished children, ..) |
| 257. Holm A, Siersma V, Bjerrum L, Cordoba G. Availability of point-of-care culture and microscopy in general practice - does it lead to more appropriate use of antibiotics in patients with suspected urinary tract infection? *Eur J Gen Pract.* 2020;26(1):175-181. | Wrong population (adults, healthy children, malnourished children, ..) |
| 258. Guarino S, Capalbo D, Scalzone E, et al. Pilot study showed that poor feeding, especially with leucocyturia, increased the odds of non-febrile urinary tract infections in children who were not toilet trained. *Acta Paediatr.* 2020;109(3):602-606. | Wrong population (adults, healthy children, malnourished children, ..) |
| 259. Abuzeyad FH, Ashraf MK, Ebrahim A, et al. Clinical presentation, culture and sensitivity pattern of urinary tract infection. *Bahrain Medical Bulletin.* 2020;42(1):20-23. | Wrong population (adults, healthy children, malnourished children, ..) |
| 260. Al-Tikrity TAH, Al-Douri MTA, Hilal NN, Abdul-Aziz MM. Diagnostic values of some immunological markers in patients with urinary tract infection. *International Journal of Drug Delivery Technology.* 2019;9(4):635-639. | Wrong population (adults, healthy children, malnourished children, ..) |
| 261. Mahajan P, Tzimenatos L, Cruz AT, et al. Prevalence of bacteremia and meningitis in febrile infants ≤ 60days with positive urinalyses in a multicenter network. *Pediatrics.* 2021;147(3):509-510. | Editorial/commentary/letter/conference abstract |
| 262. Curley T, Forster C, Pohl HG, Chamberlain J, Hamdy RF. Urinary symptom management in children with neuropathic bladder presenting to the emergency department. *Open Forum Infectious Diseases* 2020;7(SUPPL 1):S690. | Editorial/commentary/letter/conference abstract |
| 263. Nji CP, Assob JCN, Akoachere J-FTK. Predictors of Urinary Tract Infections in Children and Antibiotic Susceptibility Pattern in the Buea Health District, South West Region, Cameroon. *Biomed Research International.* 2020;2020. | Wrong study design (case-control study, epidemiologic study, qualitative study, merely prognostic outcome, sample size below 50 patients) |
| 264. Alghounaim M, Ostrow O, Timberlake K, Richardson SE, Koyle M, Science M. Antibiotic Prescription Practice for Pediatric Urinary Tract Infection in a Tertiary Center. *Pediatr Emerg Care.* 2021;37(3):150-154. | Editorial/commentary/letter/conference abstract |
| 265. Sarbu V, Domilescu I, Bagiu I, et al. Urinalysis under the Microscope: Fast, Cheap and Common but Still Relevant. *Medical-Surgical Journal-Revista Medico-Chirurgicala.* 2020;124(4):527-533. | Necessary data not reported (no 2x2 table extraction possible, data not reported for UTI separately,…) |
| 266. Pintos C, Mintegi S, Benito J, Aranzamendi M, Bonilla L, Gomez B. Blood enterovirus polymerase chain reaction testing in young febrile infants. *Arch Dis Child.* 2021. | Wrong index tests |
| 267. Ramgopal S, Noorbakhsh KA, Pruitt CM, Aronson PL, Alpern ER, Hickey RW. Outcomes of Young Infants with Hypothermia Evaluated in the Emergency Department. *J Pediatr.* 2020;221:132-+. | Wrong setting (not relevant for ambulatory care, intensive care, admitted patients) |
| 268. Lee JH, Park YS, Kwon H, Suh D, Choi YJ, Kwak Y. Clinical predictors to estimate the probability of urinary tract infection in pediatric febrile patients younger than 2 years old: Retrospective analysis of fever registry. *Eur J Pediatr.* 2019;178(11):1715-1716. | Editorial/commentary/letter/conference abstract |
| 269. Murphy AE, Stultz J, Dannenberg J, et al. Development of a clinical pathway for diagnosis and treatment of urinary tract infection in children. *J Investig Med.* 2020;68(2):650-651. | Editorial/commentary/letter/conference abstract |
| 270. Kuppermann N, Dayan PS, VanBuren JM, Casper TC, Ramilo O, Mahajan PV. Validation of a prediction rule for febrile infants less than or equal to 60 days in a multicenter network. *Acad Emerg Med.* 2020;27:S43. | Editorial/commentary/letter/conference abstract |
| 271. Waterfield T, Maney JA, Lyttle MD, et al. Diagnostic test accuracy of point-of-care procalcitonin to diagnose serious bacterial infections in children. *BMC Pediatr.* 2020;20(1):487. | Wrong target condition (serious bacterial infection, vesical-urethral reflux (VUR), renal scarring, contamination ...) |
| 272. Park JS, Byun YH, Lee JY, Lee JS, Ryu JM, Choi SJ. Clinical utility of procalcitonin in febrile infants younger than 3 months of age visiting a pediatric emergency room: a retrospective single-center study. *BMC Pediatr.* 2021;21(1):109. | Wrong target condition (serious bacterial infection, vesical-urethral reflux (VUR), renal scarring, contamination ...) |
| 273. Velez Y, Waseem M, Roques E, Ciummo E, Gerber L. High proportion of false negative urinary tract infections among dilute urine samples. *Pediatrics.* 2018;142(1):2018. | Editorial/commentary/letter/conference abstract |
| 274. Schwaderer AL, Hains DS. Aptamer-based proteomics analysis reveals a urine protein signature that differentiates UTIS from culture-negative Pyuria and normal urine. *J Am Soc Nephrol.* 2019;20:726. | Wrong study design (case-control study, epidemiologic study, qualitative study, merely prognostic outcome, sample size below 50 patients) |
| 275. Malia L, Strumph K, Smith S, Brancato J, Johnson ST, Chicaiza H. Fast and Sensitive Automated Point-of-Care Urine Dips. *Pediatr Emerg Care.* 2020;36(10):486-488. | Double (duplicate study or multiple publication on same data) |
| 276. Chang SSY, Lim AZ, Ong GY-K, et al. Predictors of serious bacterial infections using serum biomarkers in an infant population aged 0 to 90 days: A prospective cohort study. *BMJ Paediatrics Open.* 2021;5(1). | Wrong target condition (serious bacterial infection, vesical-urethral reflux (VUR), renal scarring, contamination ...) |
| 277. Demir H, Tasar MA. The Significance of Clinical and Laboratory Findings in Predicting Serious Bacterial Infections in Children With Acute Fever Without a Focus. *Journal of Pediatric Infection.* 2020;14(4):215-223. | Wrong target condition (serious bacterial infection, vesical-urethral reflux (VUR), renal scarring, contamination ...) |
