# Supplementary material for: Diagnostic value of biomarkers for paediatric urinary tract infections in primary care: systematic review and meta-analysis
Source: BMC Fam Pract. 2021 Sep 27;22:193. doi: 10.1186/s12875-021-01530-9 (PMC8474745; doi:10.1186/s12875-021-01530-9)
Supplement: Supplementary file 4 — Additional file 4: Table S4. Characteristics of included studies. [file 12875_2021_1530_MOESM4_ESM.docx]

**Additional file 4. (Table S4).** Characteristics of included studies

**Table S4. Characteristics of included studies**

| **Study** | **Setting; country** | **Design** | **Age range,**  (median/mean^a^)  **Gender**  (No. (%) boys) | **Sample size, prevalence**  (No. (%) UTI) | **Index test(s)** | **Target condition(s)** |
| --- | --- | --- | --- | --- | --- | --- |
| **Amin *et al.* 2020^87^** | OD;  Egypt | Prosp,  random, cx | 2-7y  754 (62.8%) | 1200;  84 (7) | LE(NR) ; N | UTI= urine culture (threshold not reported) (MS) |
| **Anacleto *et al.* 2009**^72^ | OD;  Philippines | Prosp, cons, cx | 4m-7y (5y)  112 (56.0) | 200;  97 (48.5) | Uricult POCT ≥10^4^cfu/ml | UTI= urine culture 1 organism ≥10^4^cfu/ml (SPA, UC, CC, MS) |
| **Andreola *et al.* 2007**^20^ | ED;  Italy | Prosp,  cons, cx | 7d-3y (10m)  37 (50) | 74;  50 (69) | CRP ≥20/40/80mg/l; PCT ≥0.5/1/2ng/ml; WBCc ≥15 000/µl; ANC≥10 000/µl | APN= urine culture 1 pathogen ≥10^5^cfu/ml in 2 samples (BS, MS) and DMSA scan hypocaptation |
| **Antwi *et al.* 2008**^33^ | OD;  Ghana | Prosp  cons, cx | 3m-5y (20m)  126 (54.8) | 230;  30 (13.0) | LE (NS); N; WBCu>10/hpf | UTI= urine culture 1 organism ≥10^5^cfu/ml (MS); ≥10^4^cfu/ml (UC) |
| **Armengol *et al.* 2001**^34^ | ED;  USA | Retrosp cons, cx | <4y (NR)  NR | 260;260;  30 (11.5) | LE≥trace/1+; N; WBCu>5/hpf; any B | UTI= urine culture pathogen ≥10^4^cfu/ml (UC) |
| **Bachur *et al.* 2001**^35^ | ED;  USA | Retrosp  cx | <2y (11m)  NR | 8815;  705 (8.0) | LE ≥1+; N; WBCu≥5/hpf | UTI= urine culture 1 pathogen ≥10^3^cfu/ml (SPA); ≥10^4^cfu/ml (UC); ≥10^5^(MS) |
| **Bonsu *et al.* 2007**^36^ | ED;  USA | Retrosp cons | 0-89 d (NR)  NR | UTI  1516; 258 (17.0)  urosepsis  1516; 12 (0.01) | WBCu ≥10/hpf | UTI= urine culture ≥10^4^cfu/ml (UC); ≥10^5^cfu/ml (CC); ≥10^3^cfu/ml (SPA); Urosepsis= same organism recovered from urine and blood culture |
| **Bressan *et al.* 2009**^22^ | ED;  Italy | Prosp,  cx | 7d-3y (5m)  31 (43) | 72;  52 (72) | PCT≥0.5/1/2ng/ml | APN= DMSA scan renal hypocaptation |
| **Bulloch *et al.* 2000**^37^ | ED;  USA | Prosp, conv, cx | 1m-19y (NR)  36 (22.6) | 159;  29 (18.2) | LE≥1+; N; WBCu>5/hpf | UTI= urine culture ≥10^4^cfu/ml (UC); ≥10^5^cfu/ml (MS) |
| **Cannon *et al.* 1986**^73^ | ED, OD;  USA | Prosp | NR  NR | 306;  44 (14.4) | LE(NS), N | UTI= urine culture 1 organism ≥10⁵cfu/ml |
| **Cantey *et al.* 2015**^38^ | HC;  USA | Retrosp, cons, cx | ≤19y (4y)  194 (63.0) | 308;  39 (12.7) | LE≥trace+; N; WBCu>10/oif; >any B (Gram stain) | UTI= urine culture pathogen ≥5x10^4^cfu/ml (UC,CC) |
| **Chaudhari *et al.* 2017**^74^ | ED;  USA | Retrosp, cons, cx | <2y (6m)  3282 (39.6) | 7599;  612 (8.1) | LE≥1+; N | UTI= urine culture any cfu/ml (SPA); 1 pathogen ≥5x10^4^cfu/ml (UC) |
| **Chaudhari *et al.* 2018**^24^ | ED;  USA | Retrosp,  cx | <2y (6m)  1012 (33.4) | 2554;  494 (19.3) | WBCu*≥*5/hpf; B≥1+/2+/3+ | UTI= urine culture 1 pathogen ≥5x10^4^cfu/ml (UC) |
| **Chen *et al*. 2013**^94^ | ED;  Taiwan | Prosp, cons, cx | 1m-10y (87%<5y)  56 (41.2) | 136;  87 (64.0) | CRP >20/50/100 mg/l;  PCT ≥0.5/1.3/2 ng/ml;  WBCc≥12 000/16 500/ 20 000/µl | APN= DMSA scan renal hypocaptation |
| **Dayan *et al.* 2000**^39^ | ED;  USA | Prosp,  conv | 1-4y (98d)  50 (58) | 85;  6 (7) | WBCu≥5; ≥10/hpf; B (Gram stain) | UTI= urine culture ≥5x10^4^cfu/ml (UC) |
| **Dayan *et al.* 2002**^23^ | ED;  USA | Prosp, cons, cx | ≤60d (36%≤30d)  114 (49) | 232;  27 (11.6) | LE≥trace; N; WBCu ≥5,≥10/hpf; any B (Gram stain) | UTI= urine culture ≥10^4^cfu/ml UC ; ≥10^3^cfu/ml SPA |
| **De *et al.* 2013**^95^ | ED;  Australia | Prosp,  cons, cx | <5y (78.5% <3y)  1611 (44.1) | 3653;  362 (9.9) | NICE Traffic light score with N, LE | UTI= urine culture any cfu/ml (SPA); ≥10⁴cfu/ml (UC); ≥10⁵cfu/ml (CC, MS) |
| **Diaz *et al.* 2016**^88^ | ED;  Spain | Retrosp | <3m (43d^a^)  190 (60.0) | 318;  76 (23.9) | CRP>30mg/l; PCT>0.5, >2ng/ml | UTI= urine culture ≥5x10^4^cfu/ml (SPA, UC) |
| **Dobbs *et al.* 1987**^75^ | HC;  UK | Prosp,  cx | 0-14y (NR)  NR | 75;  16 (21) | N, Hb, protein | UTI= urine culture ≥10⁵cfu/ml (MS); ≥10⁴cfu/ml to ≥10⁵cfu/ml and ≥10^2^wbc/mm³ (MS) |
| **Doley *et al.* 2003**^76^ | ED;  Australia | Retrosp, cons | <10y (NR)  NR | 160 <2y:  NR (15.0)  215 2-10y:  NR (7.0) | N; blood≥trace; protein≥trace; LE≥trace | UTI= urine culture >10^5^cfu/ml (BS, CC, SPA) |
| **Duong *et al.* 2016**^40^ | ED;  Belgium | Prosp, cons, cx | ≤16y (44m)  508 (41.0) | 1247;  221 (17.7) | LE≥trace+; N; WBCu≥35/µl, ≥100/µl; CRP≥40 mg/l,≥100mg/l | UTI= urine culture 1 pathogen any cfu/ml (SPA) ≥10^5^cfu/ml (UC, CC) |
| **Felt *et al.* 2017**^41^ | ED;  USA | Prosp, conv, cx | <36m (9m)  54 (30.0) | 193;  15 (7.8) | N or LE>trace or WBCu>10/hpf or any B | UTI= urine culture any cfu/ml (SPA, UC) |
| **Festo *et al.* 2011**^42^ | HC;  Tanzania | Prosp, cons, cx | 2m-5y (18m)  194 (52.4) | 370;  147 (39.7) | LE(NS); N; WBCu≥5/hpf | UTI= Urine culture ≥10⁵cfu/ml (53% MS) or any cfu/ml (47% SPA) |
| **Gervaix *et al.* 2001**^89^ | ED; Switzerland | Prosp, cons, cx | 1w-16y (NR)  18 (33) | 54;  34 (63) | CRP≥400mg/l; PCT≥0.5ng/ml | APN= DMSA scan renal hypocaptation |
| **Glissmeyer *et al.* 2014**^43^ | HC;  USA | Retrosp,  cx | <90d (27%1-28d)  NR | 6394;  770 (12.0) | LE≥trace; N; WBCu≥10/hpf; any B | UTI= urine culture pathogen ≥10^5^cfu/ml (UC) |
| **Hay *et al.* 2016**^11^ | FP,ED,WC  UK | Prosp, cons, cx | <5y  CC (94%>2y);  1267 (46.2)  NP *(*82%<2y);  1183 (52.0) | CC 2740;  60 (2.2)  NP 2277;  30 (1.3) | LE≥trace/1+/2+; N; DUTY score +dipstick | UTI= urine culture 1 pathogen ≥10⁵cfu/ml (NP,CC,BS) or ≥10⁵cfu/ml with 1000-fold difference between the growth of this and the next species (NP, CC, BS) |
| **Hernandez-Bou *et al.* 2014**^90^ | ED;  Spain | Retrosp,  cx | 29d-3m(52%<2m)  NR | 266;  6 (0.023) | PCT ≥0.7ng/ml | Urosepsis= positive blood culture with the same organism recovered from the urine |
| **Herreros *et al.* 2018**^77^ | ED;  Spain | Prosp, cons, cx | <3m (40d)  42 (70) | 57;  37 (65) | LE (≥1+), N | UTI= urine culture 1 pathogen ≥10^5^cfu/ml (CC) |
| **Hildenwall *et al.* 2017**^17^ | OD;  Tanzania | Prosp, cons, cx | 3m-5y (20m)  NR | 315;  24 (7.6) | CRP≥20 mg/l;  WBCc <5 or >17.4x10^9^/liter | UTI= urine culture1 pathogen ≥10^5^cfu/ml (CC) |
| **Hoberman *et al.* 1993**^44^ | ED;  USA | Prosp, cons, cx | ≤1y (68% >2m)  526 (55.7) | 856;  50 (5.8) | WBCu≥≥1, ≥5, ≥10/hpf; Any B | UTI= urine culture ≥10⁴cfu/ml (UC) |
| **Hoberman *et al.* 1996**^45^ | ED;  USA | Prosp, cons, cx | <2y (NR)  NR | 4253;  212 (5.0) | WBCu ≥10/µl, ≥any B (Gram stain) | UTI= urine culture 1 pathogen ≥5x10^4^cfu/ml (UC) |
| **Kanegaye *et al.* 2014**^46^ | ED;  USA | Prosp, conv, cx | ≤4y (8m)  140 (41.0) | 342;  42 (12.3) | LE≥trace/1+/2+; N; WBCu ≥10, ≥25, ≥100/µl; B ≥50, ≥100/µl | UTI= urine culture ≥5x10^4^cfu/ml (UC) |
| **Kuppermann *et al.* 2019**^96^ | ED;  USA | Prosp, cons, cx | <60d (36d^a^)  535 (58.6) | 908 (der) ; 69 (7.6)  913 (val);  82 (9.0) | ANC ≤4090/μl, PCT ≤1.71ng/ml, LE, N and <5WBCu/hpf | UTI= urine culture 1 pathogen ≥10^3^cfu/ml (SPA); ≥5x10^4^cfu/ml (UC) ; ≥10^4^ to 5x10^4^cfu/ml (UC) and LE or nitrite or >5WBCu/hpf |
| **Lagos *et al.* 1994**^78^ | ED;  Chile | Prosp,  cx | <15y (59% <5y)  737 (74.0) | 990;  348 (35.2) | LE≥trace; N | UTI= urine culture ≥10⁵cfu/ml (MS, BS or SPA) and ≥10WBC/mm³ or 2 positive cultures within 5d free of antibiotics |
| **Littlewood *et al.* 1977**^47^ | OD;  UK | Prosp | 2w-14y (NR)  NR | 189;  38 (20.1) | B>10 per field | UTI= urine culture ≥10^5^cfu/ml(BS, CC, MS,SPA) |
| **Lo *et al.* 2018**^48^ | ED;  Brazil | Prosp, cons, cx | 0-3m (1.5m^a^)  399 (76.9) | 519;  65 (12.5) | N; WBCu≥10/µl | UTI= urine culture ≥10⁵cfu/ml (UC) |
| **Lockhart *et al.* 1995**^49^ | ED;  USA | Prosp,  cx | <6m (NR)  NR | 207;  18 (8.7) | LE≥trace; N; B (Gram stain) ≥1/oif; WBCu>5/hpf; | UTI= urine culture 1 pathogen ≥10^3^cfu/ml (SPA,UC) |
| **Lohr *et al.* 1993**^50^ | OD;  USA | Retrosp,  cx | 1m-16y (NR)  NR | 689;  102 (14.8) | LE≥trace; N;  B (Gram stain) ≥1/oif; WBCu>5/hpf | UTI= urine culture 1 pathogen >10^2^cfu/ml (SPA); >10^3^cfu/ml (UC); >10^4^cfu/ml (MS) |
| **Lubell *et al.* 2017**^51^ | ED;  USA | Prosp, conv,cx | <2y (213d)  118 (45.0) | 260;  35 (13.5) | LE≥trace/1+/2+/3+;N; B (Gram stain); NGAL>39.1ng/ml | UTI= urine culture 1 pathogen ≥10^5^cfu/ml (NR) |
| **Luco *et al.* 2006**^52^ | ED;  Chile | Retrosp, cons | 11d-14y (4y^a^)  (NR) | 1173;  246 (21.0) | WBCu≥10/µl; ≥any B | UTI= urine culture 1 pathogen ≥10^3^cfu/ml (SPA); ≥10^4^ cfu/ml (UC); ≥10^5^cfu/ml (MS, BS) |
| **Lunn *et al.* 2010**^79^ | OD;  UK | Prosp, cons, cx | 0–19y (10y2m)  143 (54.0) | 280;  19 (6.8) | LE≥trace; N | UTI= urine culture pathogen ≥10^5^cfu/ml (BS,MS/CC) |
| **Malia *et al.* 2017**^53^ | ED;  UK | Retrosp,  cx | 4d-18y (NR)  77 (23.0) | 334;  35 (10.5) | LE≥trace; N ; WBCu>10/hpf | UTI= urine culture 1 pathogen ≥10^5^cfu/ml (UC, CC) |
| **Marr *et al.* 1975**^80^ | OD;  USA | Prosp,  cx | 1m-16y (NR)  (NR) | 108;  42 (38.9) | Microstix POCT: nitrite test area and culture area >10^5^cfu/ml | UTI= urine culture >10^5^cfu/ml (NR) |
| **Mcgillivray *et al.* 2005**^54^ | ED;  Canada | Prosp,  cx | <3y (18%<3m)  102 (33.6) | 303;  82 (27.1) | LE≥trace; N; WBCu>5/hpf | UTI= urine culture 1 pathogen ≥10^3^cfu/ml (UC) |
| **Mitiku *et al.* 2018**^55^ | OD;  Ethiopia | Prosp, cons, cx | <15y (5y^a^)  166 (61.7) | 269;  74 (27.5) | LE(NS); N; pH; glucose; protein; WBCu>5/hpf; RBCu>5/hpf | UTI= urine culture 1 organism ≥10^5^cfu/ml (MS) |
| **Molyneux *et al*. 1995**^56^ | ED;  UK | Prosp,  cx | NR | 248;  19 (7.7) | LE(NS); WBCu>100/cmm-1 | UTI= urine culture 1 organism (MS, BS) |
| **Musa Aisien *et al.* 2003**^57^ | ED;  Nigeria | Prosp, cons, cx | 1m-5y (18m)  177 (59.0) | 300;  26 (8.7) | WBCu≥5, ≥10/µl | UTI= urine culture ≥10⁵cfu/ml (SPA, CC or MS) |
| **Nadeem *et al.* 2021**^86^ | ED;  USA | Retro, cons, cx | <2y  9955 (41.2) | 24 171;  2003 (8.3) | LE≥trace, 1+; N;  WBCu ≥5, ≥10/hpf | UTI= urine culture 1 pathogen ≥5x10^4^cfu/ml (UC), ≥10⁵cfu/ml (MS) |
| **Nijman *et al.* 2018**^91^ | ED; Netherlands | Prosp,  cons, cx | 1m-16y (3y^a^)  606 (55.9) | 1085;  (5.2) | CRP ≥20/80mg/l; PCT ≥0.5/2ng/ml | UTI= urine culture pathogen (NR) |
| **Nikfar *et al.* 2010**^92^ | HC;  Iran | ncc | 1m-14y (NR)  19 (19) | 100;  63 (63) | CRP>20mg/l; PCT>0.5ng/ml | APN= DMSA scan renal hypocaptation |
| **Paalanne *et al.* 2020**^85^ | ED;  Finland | Prosp,  cx | Children that wore diapers (11.8*m)  153 (44.8) | 565 ;  143 (25.3) | Diaper embedded dipstick test: (Tena-U); LE(NR); N; | UTI= urine culture ≥10^4^cfu/ml in 2 subsequent urine samples (4%CC, 86%NP) or ≥10^5^cfu/ml in 1 sample (4%CC, 86%NP) or any cfu/ml (10%SPA) |
| **Pylkkanen *et al.* 1979**^58^ | OD;  Finland | Prosp,  cx | ≤18y (64.5≤2y)  NR | 477;  322 (67.5) | WBCu>10/µl; >2/hpf; B (any) | UTI= urine culture any cfu/ml (Uricult and blood agar plate; MS, SPA) |
| **Ramlakhan *et al.* 2011**^81^ | ED;  UK | Retrosp, cons, cx | ≤2y (9m^a^)  119 (37.0) | 321;  78 (24.3) | LE≥trace; N; blood; protein | UTI= urine culture 1 pathogen ≥10⁵cfu/ml (BS, CC) ≥10^4^cfu/ml (UC, SPA) |
| **Reardon *et al.* 2009**^59^ | ED;  USA | Prosp,  cons, cx | <2y (13m^a^)  NR | 435;  45 (10.3) | LE≥trace; N; WBCu>5/hpf | UTI= urine culture ≥10^4^cfu/ml (UC) |
| **Saxena *et al.* 1975**^60^ | OD;  India | Prosp | 4-12y (21%<5y)  27 (39) | 70;  26 (37) | WBCu>10µl | UTI= urine culture (MS) |
| **Shah *et al.* 2014**^25^ | ED;  USA | Prosp, cons, cx | 0-19y (81%<2y)  232 (33.0) | 703;  49 (7.0) | WBCu≥10/µl and B (Gram stain) ≥any/oif; WBCu ≥2/hpf and B≥any/oif | UTI= urine culture 1 pathogen ≥5x10^4^cfu/ml(UC,MS) |
| **Shaikh *et al.* 2018**^16^ | ED;  USA | Retrosp,  ncc | 2m-2y  (der: 73%<1y val: 60%<1y)  470 (27.9) (der)  93 (24.2) (val) | 1686 (der);  542 (32.1)  384 (val);  30 (7.8) | UTIcalc with urine dipstick, Gram stain, and WBCu;  LE≥trace | UTI= urine culture ≥5x10^4^cfu/ml (UC) and pyuria |
| **Shaikh *et al.* (a) 2019**^82^ | ED;  USA | Retrosp,  cons, cx | <2y (7m)  3335 (33.1) | 10 078;  617 (6.1) | LE≥1+ | UTI= urine culture 1 pathogen ≥5x10^4^cfu/ml (UC) ≥10⁵cfu/ml (CC) |
| **Shaikh et al. (b) 2019**^61^ | ED;  USA | Prosp, conv, cx | 1m-10y (NR)  NR | 56;  35 (63) | IL-1; CCL2; CXCL9; CXCL1; HGF; CXCL12; INF-Ƴ; IL-2R alfa; NGAL | APN= DMSA scan renal hypocaptation without cortical loss (within 14d) |
| **Shaw *et al.* 1991**^62^ | ED;  USA | Prosp, cons, cx | 14d-19y (6y4m^a^)  NR (37) | 491;  45 (9.2) | LE≥ 1+; N; WBCu>5 or 10/hpf; B | UTI= urine culture 1 or 2 pathogen(s) ≥10^3^cfu/ml (UC); ≥10⁵cfu/ml (CC, MS, BS) |
| **Shaw *et al.* 1997**^63^ | ED;  USA | Prosp, cons, cx | 2d-19y (50%≤2y)  NR (47) | 1298;  92 (7.1) | LE; N; B or WBCu≥5/hpf; Filtra-check POCT | UTI= urine culture 1 pathogen ≥10^4^cfu/ml (UC) ≥10^5^cfu/ml (MS, CC) |
| **Shaw *et al.* 1998**^64^ | ED;  USA | Prosp,  cx | <2y (9m^a^)  NR (26.0) | 3873;  105 (2.7) | LE≥trace; N; WBCu≥10/µl; B (Gram stain) | UTI= urine culture pathogen ≥10^4^cfu/ml (99%UC, 1%MS) |
| **Tzimenatos *et al.* 2018**^65^ | ED;  USA | Prosp,  conv, cx | ≤2m (38d)  2376 (57.3) | UTI  4147; 289 (7.0)  Urosepsis  4147; 27 (0.007) | LE≥trace/2+/3+; N; >5WBCu/hpf | UTI=urine culture pathogen ≥5x10^4^cfu/ml (UC); ≥10^3^cfu/ml (SPA); Urosepsis= same organism recovered from urine and blood culture |
| **Velasco *et al.* 2015**^18^ | ED;  Spain | Prosp, cons, cx | <3m (46d)  2029 (59.7) | 3401;  547 (16.1) | LE≥1+; N; CRP≥20mg/l; PCT≥0.5ng/ml; ANC >10 000/µl; WBCc ≥15 000/µl | UTI= urine culture 1 pathogen ≥5x10^4^cfu/ml (UC,SPA) |
| **Velasco *et al.* 2017**^19^ | ED;  Spain | Retrosp,  cons, cx | <3m (51d^a^)  273 (69.9) | 391;  26 (0.07) | Predictive model: Well appearing, >21d, CRP≤20mg/l, PCT≤0.5ng/ml | Urosepsis= same organism recovered from urine (≥10^4^cfu/ml(UC), ≥10^3^cfu/ml (SPA) and blood or CSF culture |
| **Verbakel *et al.* 2015**^93^ | FP, ED,OD; Belgium | Prosp,  cons, cx | 1m-16y (2y)  UTI 394 (52.1)APN NR (53.0) | UTI  756; 87 (11.5)APN  6815; 55 (0.01) | CRP≥5/10/20/80/200 mg/l | UTI= urine culture ≥10⁵cfu/ml (NR)  APN= changes on DMSA scan or Doppler ultrasound compatible with pyelonephritis |
| **Vickers *et al.* 1991**^66^ | OD;  UK | Prosp, cons, cx | 6w-18y (NR)  NR | 342;  24 (7.0) | B>8/hpf | UTI= urine culture 1 organism ≥10⁵cfu/ml (BS, MS) |
| **Waisman *et al.* 1999**^67^ | ED;  Israel | Prosp, random,cx | 1m-17y (29%<2y)  39 (32.2) | 121;  35 (28.9) | LE≥trace; N; WBCu>10/hpf; Uriscreen POCT>any foam | UTI= urine culture ≥10^2^cfu/ml (SPA); ≥10^3^cfu/ml (SPA); ≥10⁵cfu/ml (BS, CC, MS) |
| **Waterfield *et al.* 2018**^21^ | ED;  Ireland | Prosp, conv, cx | <3m (42d)  NR (51.0) | 126;  12 (9.5) | CRP ≥20/50/100 mg/l; PCT ≥0.25/0.5/1 ng/ml | UTI= urine culture 1 organism ≥10^5^cfu/ml 1x UC/SPA or 2x urine pad with same organism |
| **Watson *et al.* 2016**^68^ | ED;  USA | Prosp,  cx | ≤18y (5y)  43 (21.6) | 199;  29 (14.6) | LE≥trace/1+/2+/3+; N; HD5 >174 mg/mgCr; HNP1-3 >384 pg/mgCr | UTI= urine culture 1 pathogen ≥5x10^4^cfu/ml (CC, UC) |
| **Weinberg *et al.* 1991**^69^ | HC;  USA | Retrosp, cons, cx | ≤18y (NR)  NR | 1019;  41 (4.0) | LE(NS); N; B (Gram stain) ≥1/2/5 /oif; WBCu≥5/10/hpf | UTI= urine culture ≥10^5^cfu/ml (BS, UC, SPA, MS) |
| **Williams-Smith *et al.* 2020**^84^ | ED; Switzerland | Pro,  cx | 0-3y (4.4m)  93 (53.4) | 173:  47 (27.2) | LE (≥trace); N ;  CRP≥40 mg/L;  WBCc ≥15 000/µl | UTI= urine culture 1 pathogen ≥10^4^cfu/ml (UC) |
| **Yavaş *et al.***  **2021**^83^ | OD;  Turkey | Retro, cons | 2m-18y  39 (13.6) | 287  99 (34.5) | N; WBCu ≥25/µl  CRP≥5mg/l | UTI= urine culture 1 pathogen 5x≥10^4^cfu/ml (6%UC), ≥10⁵cfu/ml (18%BS, 76%MS) |
| **Yildirim *et al.* 2008**^70^ | HC;  Turkey | Prosp,  cx | 9m-14y (6y^a^)  NR (18.4) | 239;  98 (41.0) | LE(NS); WBCu>5/hpf; CRP | UTI= urine culture ≥10^5^cfu/ml (UC, SPA, MS) |
| **Yodoshi *et al*. 2019**^71^ | ED, OD;  Japan | Retrosp,  cons, cx | ≤3y (3m)  850 (55.0) | 1546;  183 (11.8) | any B (Gram stain) and WBCu>5/oif | UTI= urine culture pathogen ≥10^4^cfu/ml (UC) |

UTI= urinary tract infection, APN= pyelonephritis, ED= Emergency department, FP= familu practices, OD= outpatient department of a hospital, HC=health centre, PO= paediatricians’ office, USA= United States America, UK= United Kingdom, Prosp= Prospective design; retrosp= Retrospective design; cx= Cross-sectional design, ncc= nested case-control, cons= Consecutive enrolment, Conv= convenience enrolment, FWS = fever without a source for infection, °C= degrees Celsius, In case median age was not presented, mean age is presented and indicated with an asterisk (*), LE = leucocyte esterase (urine dipstick), N= nitrite (urine dipstick), B=bacteria (urine), Microscopy (a) = automatic microscopy, Microscopy (m)= manual microscopy, WBCu = white blood cells (urine), RBCu= red blood cells (urine), Hb= Haemoglobin (urine dipstick), WBCc = white blood cell count (blood), ANC = Absolute Neutrophil Count, CRP = C-reactive protein, PCT= procalcitonin, NGAL= Neutrophilic Gelatinase Associated Lipocalin, KIM= Kidney Injury Molecule, HNP= Human Neutrophilic Peptides, IL= interleukin, IFN= interferon, CCL= chemokine C-C motif ligand, CXCL= C-X-C Motif Chemokine Ligand, HGF= Hepatocyte Growth Factor, IL-2 R alpha= interleukin 2 receptor alpha, RT-qPCT= Reverse transcription polymerase chain reaction, POCT= point-of-care test, UTIcalc= UTI calculator, DUTYscore= Diagnosis of Urinary Tract Infections in children, prediction rule, NICE = National Institute for Health and Care Excellence, PAT= Pediatric Assessment Triangle, UC=urethral catheterization, SPA=suprapubic aspiration, MS=midstream sample, CC= clean catch sample (first stream), BS=bag specimen, NP= nappy pad sample, l= litre, ml= millilitre, µl= microliter, mm^3^= cubic millimetre, mg=milligram, ng= nanogram, pg= picogram, pg/mgCr= picogram per mgCr, cfu= colony forming units, CSF= cerebrospinal fluid, DMSA= Di-Mercapto-Succinic-Acid, hpf= high power field, oif= oil immersion fiel
