# Supplementary material for: Diagnostic value of biomarkers for paediatric urinary tract infections in primary care: systematic review and meta-analysis
Source: BMC Fam Pract. 2021 Sep 27;22:193. doi: 10.1186/s12875-021-01530-9 (PMC8474745; doi:10.1186/s12875-021-01530-9)
Supplement: Supplementary file 5 — Additional file 5: Figures S1–21. Dumbbell plots of biomarkers, point-of-care tests and prediction rules for urinary tract infection. [file 12875_2021_1530_MOESM5_ESM.docx]

**Additional file 5. (Figures S1-21).** Dumbbell plots of biomarkers, point-of-care tests and prediction rules for urinary tract infection

**Figures S1- 21 Likelihood ratios and post-test disease probabilities (%) (dumbbell plots) of biomarkers, point-of-care tests and prediction rules for UTI**

**Figure S1 Nitrite (urine dipstick) for cystitis**

**

**

Prevalence= number of children with uncomplicated UTI /sample size (n), LR+ = positive likelihood ratio, LR- = negative likelihood ratio, 95%CI = 95% confidence intervals, UTI= urinary tract infection, *Study data from Herreros et al (2018) was not added to the meta-analysis, + = positive, - = negative

**Figure S2 Leukocyte esterase (urine dipstick) for cystitis**

**

**

Prevalence= number of children with uncomplicated UTI /sample size (n), LR+ = positive likelihood ratio, LR- = negative likelihood ratio, 95%CI = 95% confidence intervals, UTI= urinary tract infection, + = positive, - = negative, NS= not specified, *Study data from Herreros et al (2018) was not added to the meta-analysis, **When no threshold was reported for LE (NS) we assumed ‘any discoloration’ as the positivity threshold (≥trace) for the meta-analysis

**Figure S3 Protein, hemoglobin and glucose (urine dipstick) for cystitis**

**
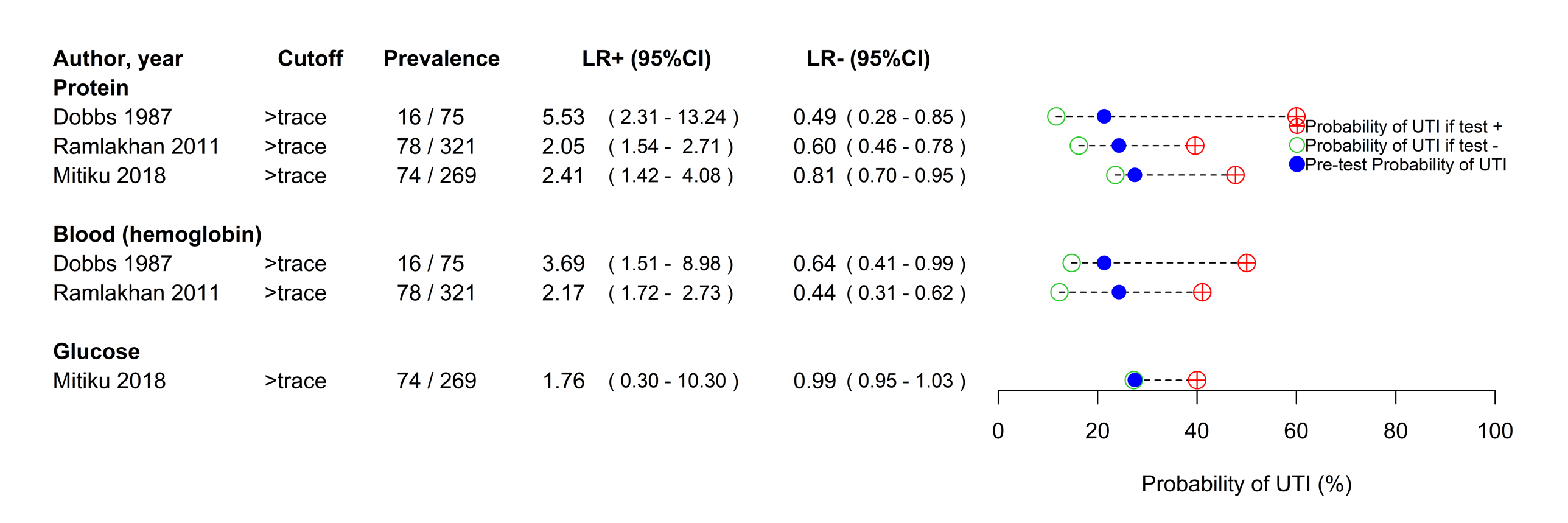
**

Prevalence= number of children with uncomplicated UTI /sample size (n), LR+ = positive likelihood ratio, LR- = negative likelihood ratio, 95%CI = 95% confidence intervals, UTI= urinary tract infection, + = positive, - = negative

**Figure S4 Combination of Leukocyte Esterase or nitrite positive for cystitis**

**

**

Prevalence= number of children with uncomplicated UTI /sample size (n), LR+ = positive likelihood ratio, LR- = negative likelihood ratio, 95%CI = 95% confidence intervals, UTI= urinary tract infection, *Study data from Herreros et al (2018) was not added to the meta-analysis, + = positive, - = negative

**Figure S5 Combination of Leukocyte Esterase and nitrite positive for cystitis**

**
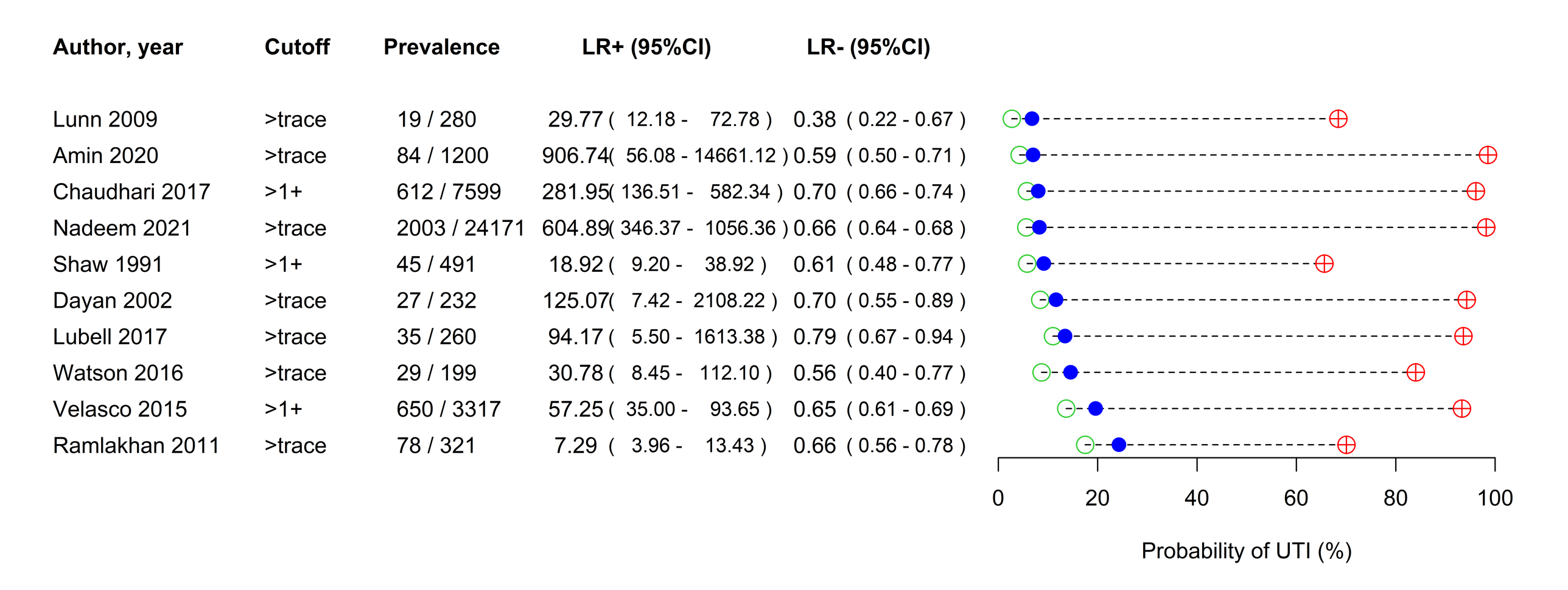
**

Prevalence= number of children with uncomplicated UTI /sample size (n), LR+ = positive likelihood ratio, LR- = negative likelihood ratio, 95%CI = 95% confidence intervals, UTI= urinary tract infection, + = positive, - = negative

**Figure S6 Combination of urine dipstick markers (Leukocyte Esterase, Nitrite, Hemoglobin and Protein) for cystitis**

**
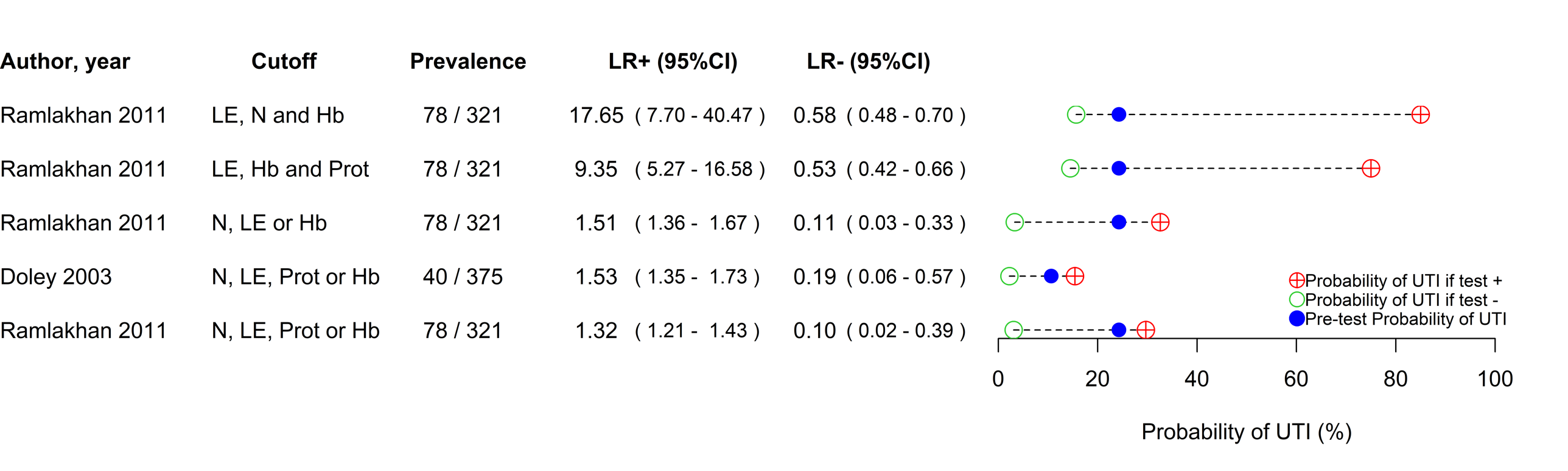
**

Prevalence= number of children with uncomplicated UTI /sample size (n), UTI= urinary tract infection, LE= Leucocyte Esterase > trace, N= Nitrite, Hb= Hemoglobin>trace, Prot= Protein>trace, n= sample size, LR+ = positive likelihood ratio, LR- = negative likelihood ratio, + = positive, - = negative

**Figure S7 Urine white blood cells (manual microscopy) for cystitis**

**

**

Prevalence= number of children with uncomplicated UTI /sample size (n), LR+ = positive likelihood ratio, LR- = negative likelihood ratio, 95%CI = 95% confidence intervals, UTI= urinary tract infection, *Study data from Pylkkanen et al. (1979) was not added to the meta-analysis, + = positive, - = negative, hpf= high power field

**Figure S8 Urine white blood cells (automatic microscopy) for cystitis**





Prevalence= number of children with uncomplicated UTI /sample size (n), LR+ = positive likelihood ratio, LR- = negative likelihood ratio, 95%CI = 95% confidence intervals, UTI= urinary tract infection, + = positive, - = negative, µl= microliter, *5 white blood cells per oil-immersion-fields (data from this study not added to the meta-analysis)

**Figure S9 Urine unstained bacteria for cystitis**

1. **Manual microscopy**

**
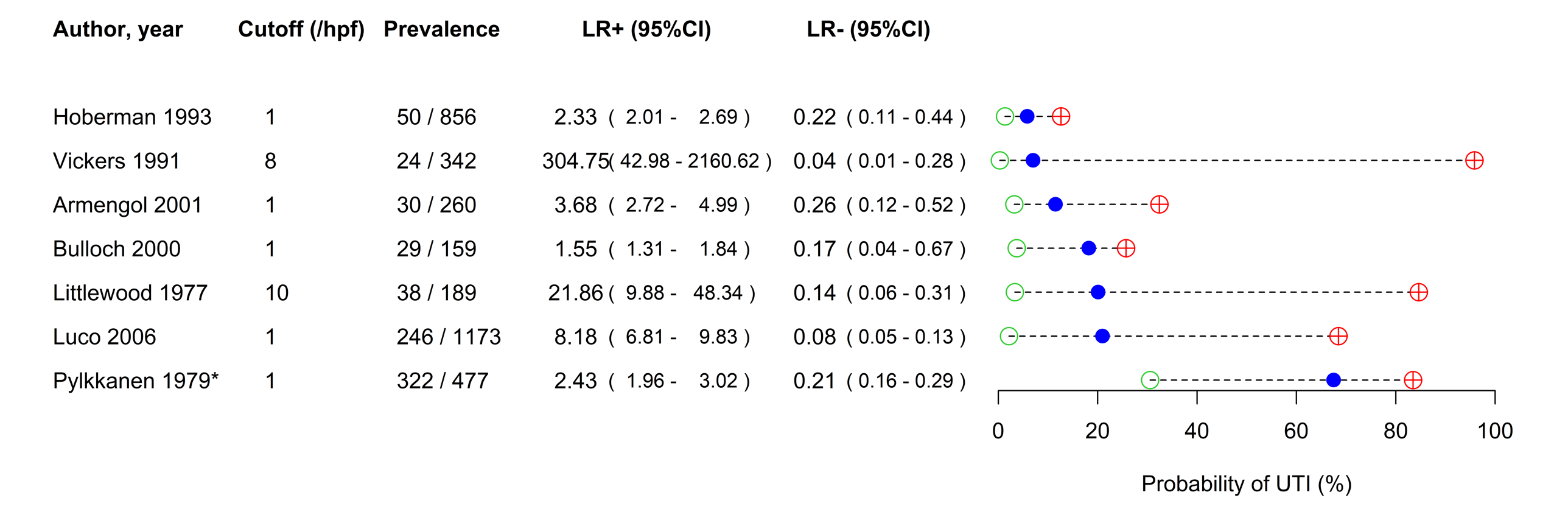
**

1. **Automated microscopy**


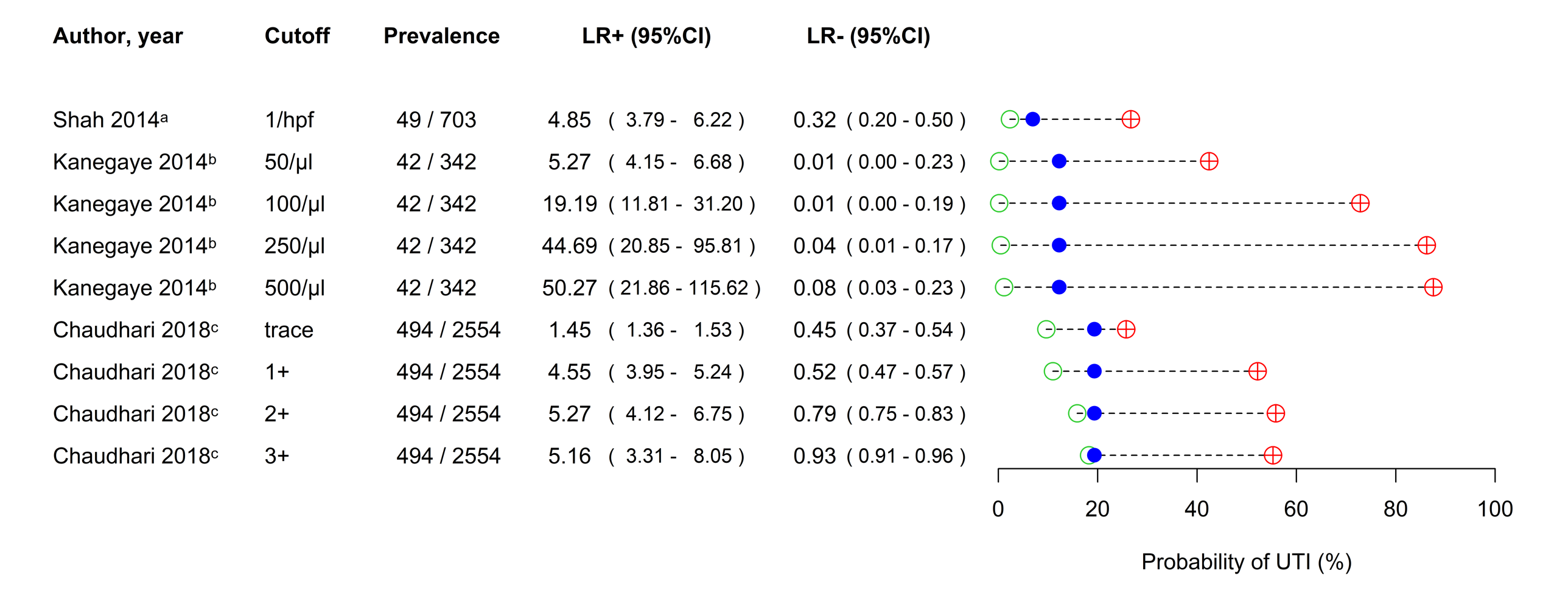


Prevalence= number of children with uncomplicated UTI /sample size (n), LR+ = positive likelihood ratio, LR- = negative likelihood ratio, 95%CI = 95% confidence intervals, UTI= urinary tract infection, *Study data from Pylkkanen et al. (1979) was not added to the meta-analysis, + = positive, - = negative, hpf = high power field, µl= microliter, ^a^= IRIS IQ200 analyzer, ^b^= UF1000i analyzer, ^c^=IQ200 analyzer

**Figure S10 Urine gram stained bacteria (microscopy) for cystitis**

**

**

Prevalence= number of children with uncomplicated UTI /sample size (n), LR+ = positive likelihood ratio, LR- = negative likelihood ratio, 95%CI = 95% confidence intervals, UTI= urinary tract infection, + = positive, - = negative, hpf = high power field

**Figure S11 Urine red blood cells, NGAL, HD-5, HNP 1-3 for cystitis**


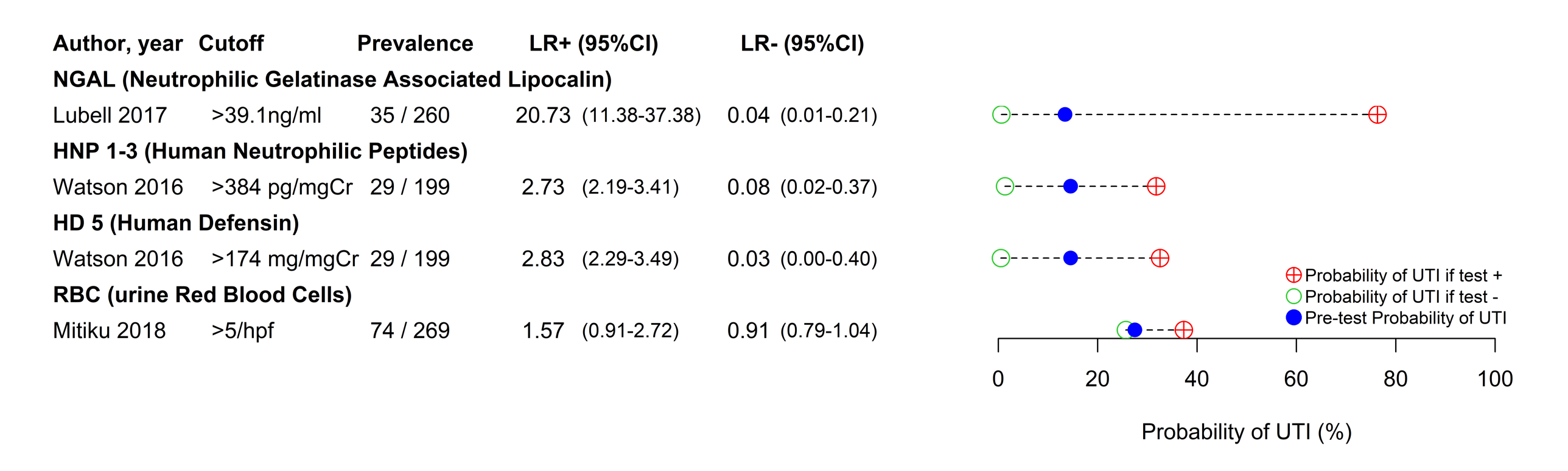
 Prevalence= number of children with uncomplicated UTI /sample size (n), LR+ = positive likelihood ratio, LR- = negative likelihood ratio, 95%CI = 95% confidence intervals, UTI= urinary tract infection, + = positive, - = negative, hpf= high power field, mg = milligram, ng= nanogram, pg= piccogram, ml= milliliter

**Figure S12 Combination of urine bacteria and white blood cells (microscopy) for cystitis**



Prevalence= number of children with uncomplicated UTI /sample size (n), LR+ = positive likelihood ratio, LR- = negative likelihood ratio, 95%CI = 95% confidence intervals, WBC= urine white blood cells, B= bacteria, B(g)= gram stained bacteria, hpf= high power field, mm^3^= cubic milliliter, UTI= urinary tract infection, + = positive, - = negative

**Figure S13 Combination of urine dipstick test with urine white blood cells for cystitis**


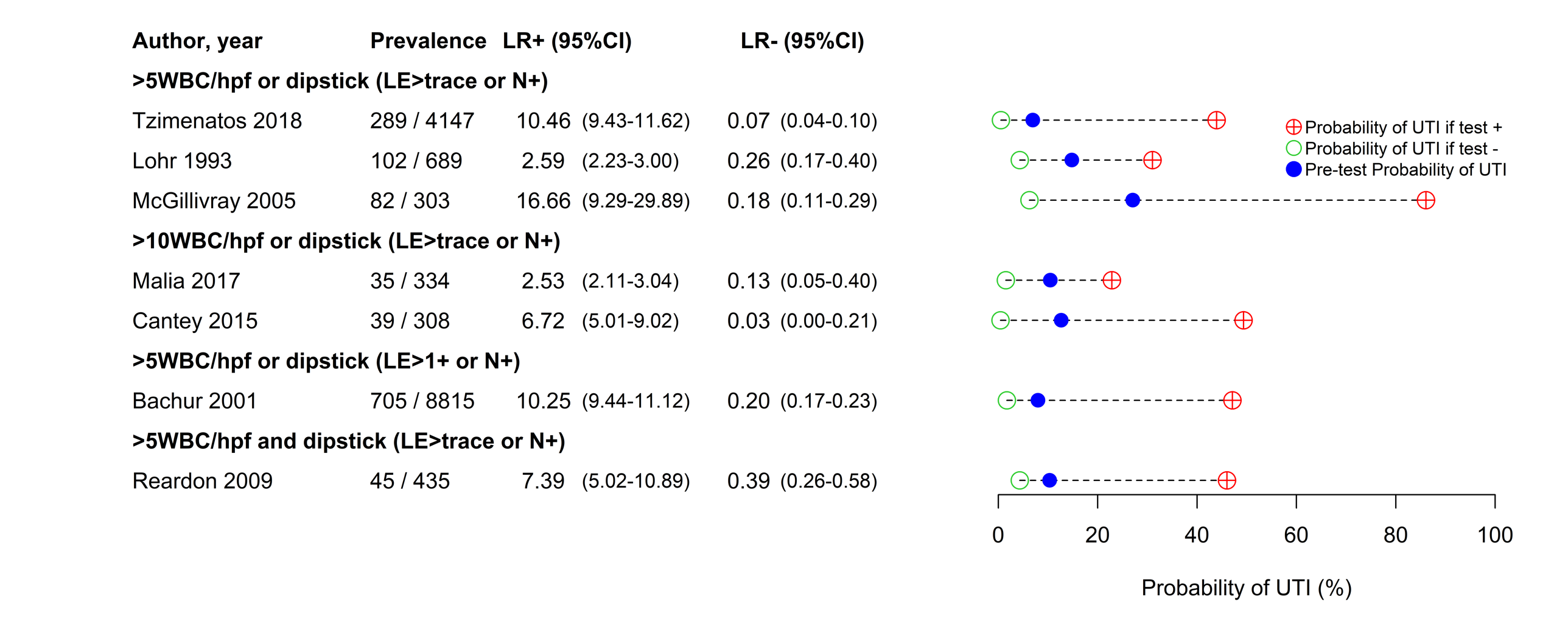


Prevalence= number of children with uncomplicated UTI /sample size (n), LR+ = positive likelihood ratio, LR- = negative likelihood ratio, 95%CI = 95% confidence intervals, UTI= urinary tract infection,+ = positive, - = negative, hpf= high power field

**Figure S14 Combination of urine dipstick test, urine bacteria and urine white blood cells for cystitis**

**
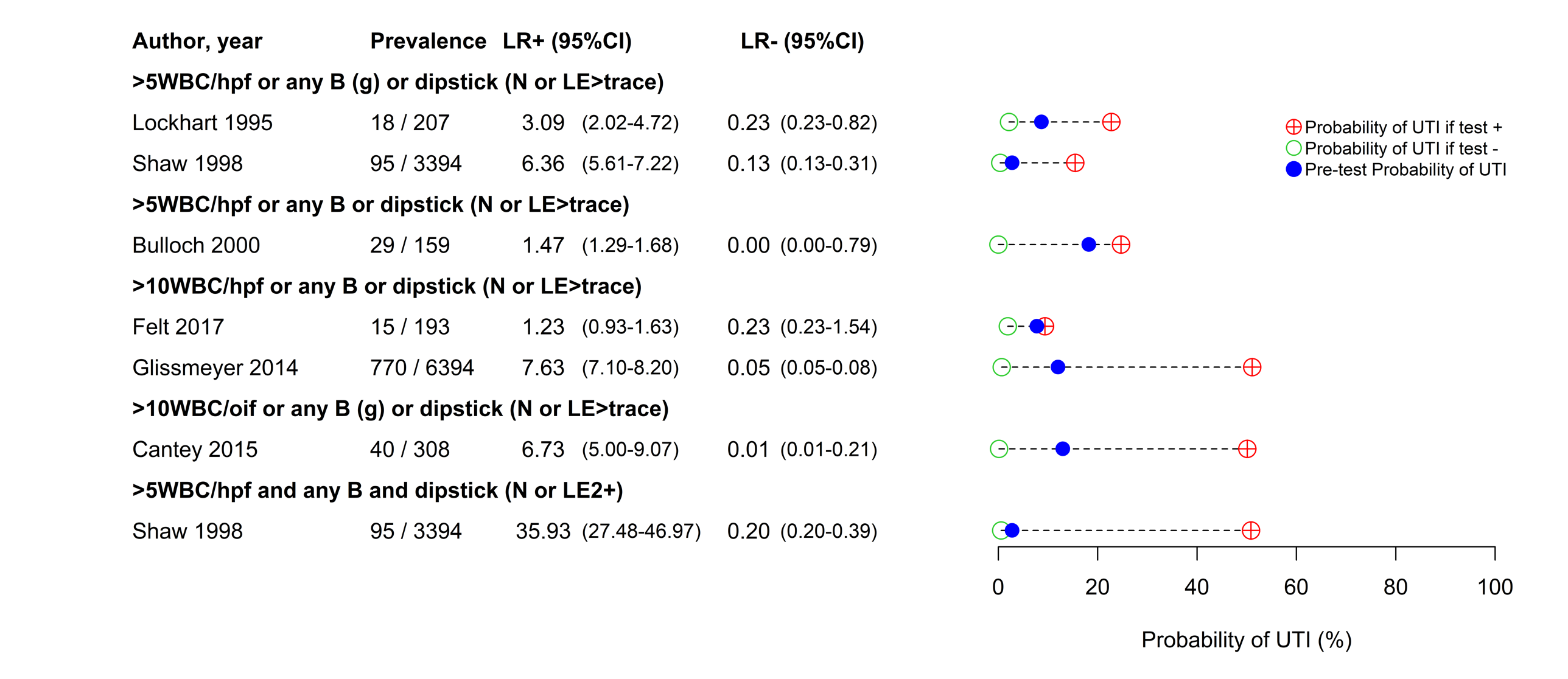
**

Prevalence= number of children with uncomplicated UTI /sample size (n), LR+ = positive likelihood ratio, LR- = negative likelihood ratio, 95%CI = 95% confidence intervals, UTI= urinary tract infection, + = positive, - = negative, hpf= high power field, oif= oil immersion fields, N= nitrite, LE= leucocyte esterase, B= bacteria unstained (urine), B (g)= gram stained bacteria (urine)

**Figure S15 C-reactive protein (blood) for cystitis**

**

**

Prevalence= number of children with uncomplicated UTI /sample size (n), LR+ = positive likelihood ratio, LR- = negative likelihood ratio, 95%CI = 95% confidence intervals, NS= not specified, UTI= urinary tract infection, *Study data from Yildirim et al. (2008) were not included in the meta-analysis because the threshold was not specified , + = positive, - = negative, mg= milligram, l= liter

**Figure S16 Procalcitonin (blood) for cystitis**

**
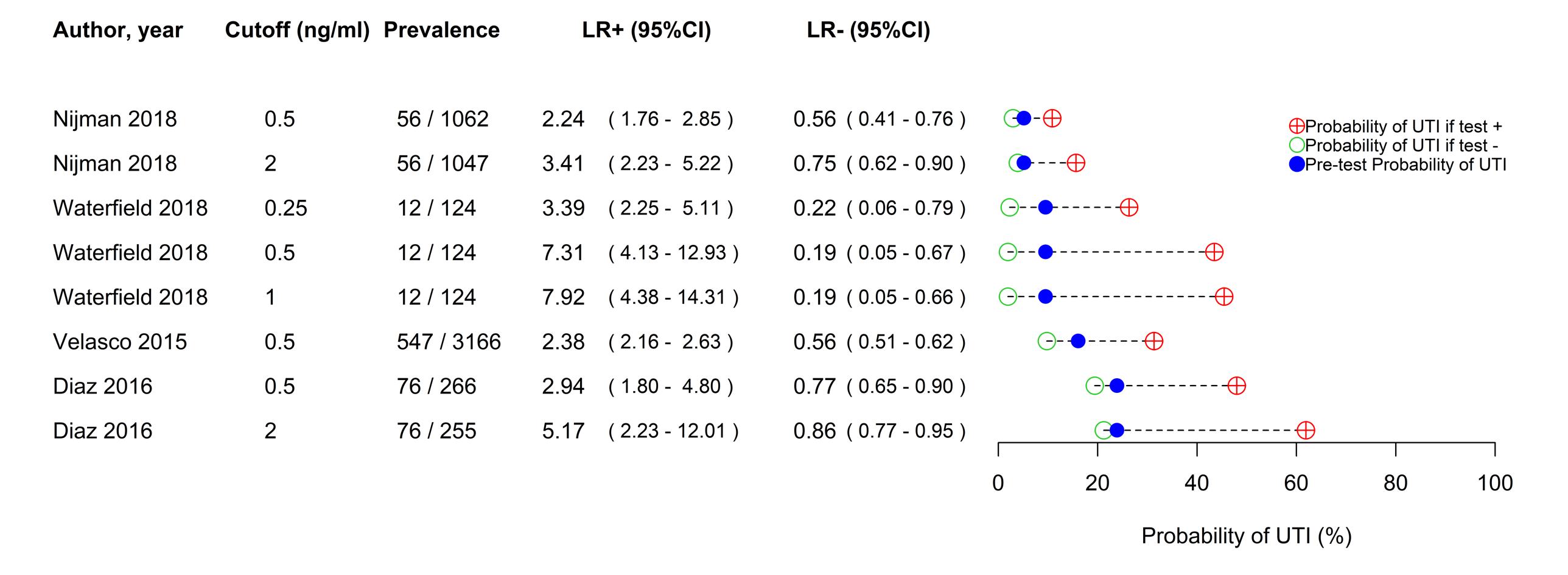
**

Prevalence= number of children with uncomplicated UTI /sample size (n), LR+ = positive likelihood ratio, LR- = negative likelihood ratio, 95%CI = 95% confidence intervals, UTI= urinary tract infection, + = positive, - = negative, ng= nanogram, ml= milliliter

**Figure S17 White blood cell count and Absolute Neutrophil Count (blood) for pyelonephritis**


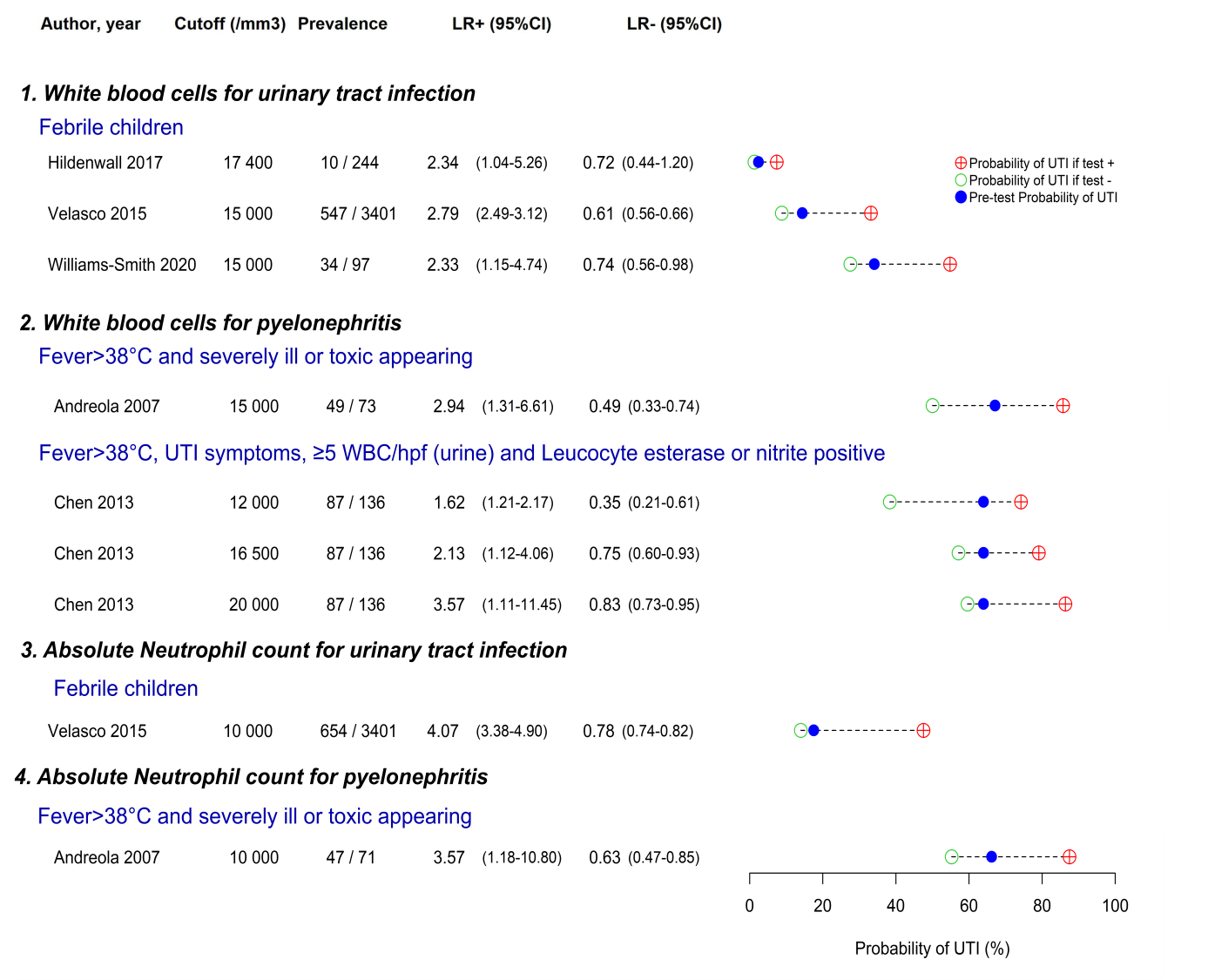


Prevalence= number of children with uncomplicated UTI /sample size (n), LR+ = positive likelihood ratio, LR- = negative likelihood ratio, 95%CI = 95% confidence intervals, UTI= urinary tract infection, + = positive, - = negative, mm^3^= cubic millimeter

**Figure S18 Urine point-of-care tests for cystitis**


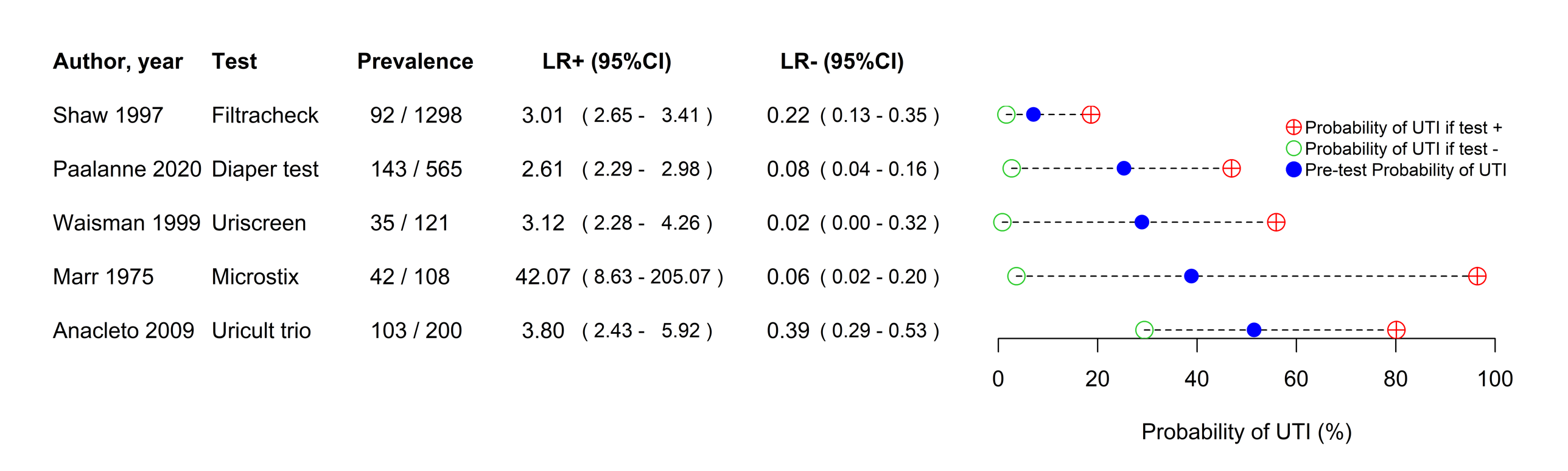


| **POCT** | **VARIABLE** | **MECHANISM** | **THRESHOLD** | **TIME** |
| --- | --- | --- | --- | --- |
| **Uriscreen** | Bacteria, RBC, WBC | Catalase activity | >continuous ring of foam formation if any variable present | 2 minutes |
| **Microstix dipslide** | Bacteria, nitrite | One nitrite test area (Griess reaction); one gram negative and one gram positive test area (culture media) | >10^5^ cfu/ml on a culture pad  >any degree of pink on nitrite test area | Nitrite: 30 seconds  Bacteria: 18-24 hours |
| **Uricult dipslide** | Bacteria | Dipslide test with 3 culture media: E. Coli, CLED and MacConkey media | >10^4^ cfu/ml on CLED medium | 24-48 hours |
| **Filtracheck** | Bacteria, WBC | Colorimetric negatively charged filter | Color intensity >1+ (scale 0 to 4+) | 2 minutes |
| **Diaper embedded test device** | Leukocyte esterase and/or nitrite | Qualitative reagent strip | Any discoloration | Directly available after urination |

POCT= point-of-care test, N= number of studies, RBC= urine red blood cells, WBC= urine white blood cells, E.Coli= Escherichia Coli, CLED= Cystine-lactose-electrolyte-deficient agar, cfu= colony forming units, ml= millilitre

Prevalence= number of children with uncomplicated UTI /sample size (n), LR+ = positive likelihood ratio, LR- = negative likelihood ratio, 95%CI = 95% confidence intervals, UTI= urinary tract infection, + = positive, - = negative

**Figure S19 Clinical prediction rules for cystitis**

**

**

| **RULE** | **VARIABLES** |
| --- | --- |
| **A** | DUTY score (for nappy pad samples) with urine dipstick test (0 to 14 points (p) ) |
| **B** | DUTY score (for clean catch samples) with urine dipstick test (0 to 13 points (p) ) |
| **C** | ANC ≤4090/μl, PCT ≤1.71ng/ml, LE negative, N negative and <5WBC/hpf |
| **D** | NICE traffic light (amber or red colour) or dipstick (N positive) |
| **E** | NICE traffic light (amber or red colour) or dipstick (LE>trace) |
| **F** | NICE traffic light (amber or red colour) or dipstick (N positive or LE>trace) |
| **G** | NICE traffic light (amber or red colour) or dipstick (N positive and LE>trace) |
| **H** | UTIcalc with dipstick (LE>1+ or nitrite positive) |
| **I** | UTIcalc with dipstick (LE>1+ or nitrite positive) or gram stain (>any bacteria) |
| **J** | UTIcalc with dipstick (LE>1+ or nitrite positive) or urine white blood cell count (>10/µl) |
| **K** | UTIcalc with dipstick (LE>1+ or nitrite positive) or urine white blood cell count (>10/µl) or gram stain (>any bacteria) |

Prevalence= number of children with uncomplicated UTI /sample size (n), LR+ = positive likelihood ratio, LR- = negative likelihood ratio, 95%CI = 95% confidence intervals, UTI= urinary tract infection, p= points, NCC= nested case control study, Prosp= prospective study, Retrosp= retrospective study, der= derivation study, val= validation study, y= year, NICE= National Institute for Health and Care Excellence, + = positive, - = negative, μl = microliter, ml= milliliter, ng= nano gram, LE= leucocyte esterase, N= nitrite, ANC= absolute neutrophil count, PCT= procalcitonin, WBC= white blood cell

**Figure S20 Urine biomarkers for pyelonephritis (data from Shaikh et al. 2019b^50^)**


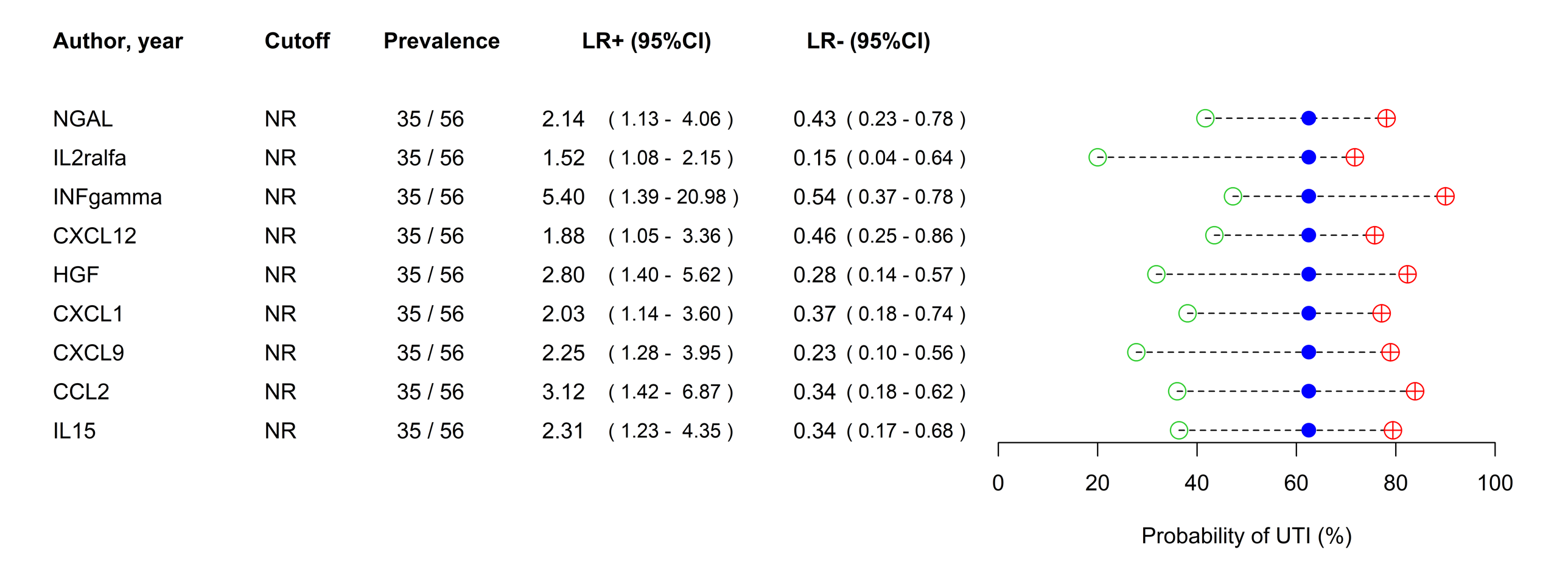
Prevalence= number of children with uncomplicated UTI /sample size (n), LR+ = positive likelihood ratio, LR- = negative likelihood ratio, 95%CI = 95% confidence intervals, UTI= urinary tract infection, NR= not reported, NGAL= Neutrophil Gelatinase Associated Lipocalin, IL= Interleukin, IFN=interferon, CXCL=chemokine, HGF= Hepatocyte growth factor, CCL= urinary Chemokine (C-C Motif) Ligand

**Figure S21 Biomarkers for urinary tract infection with associated bacteraemia**

**
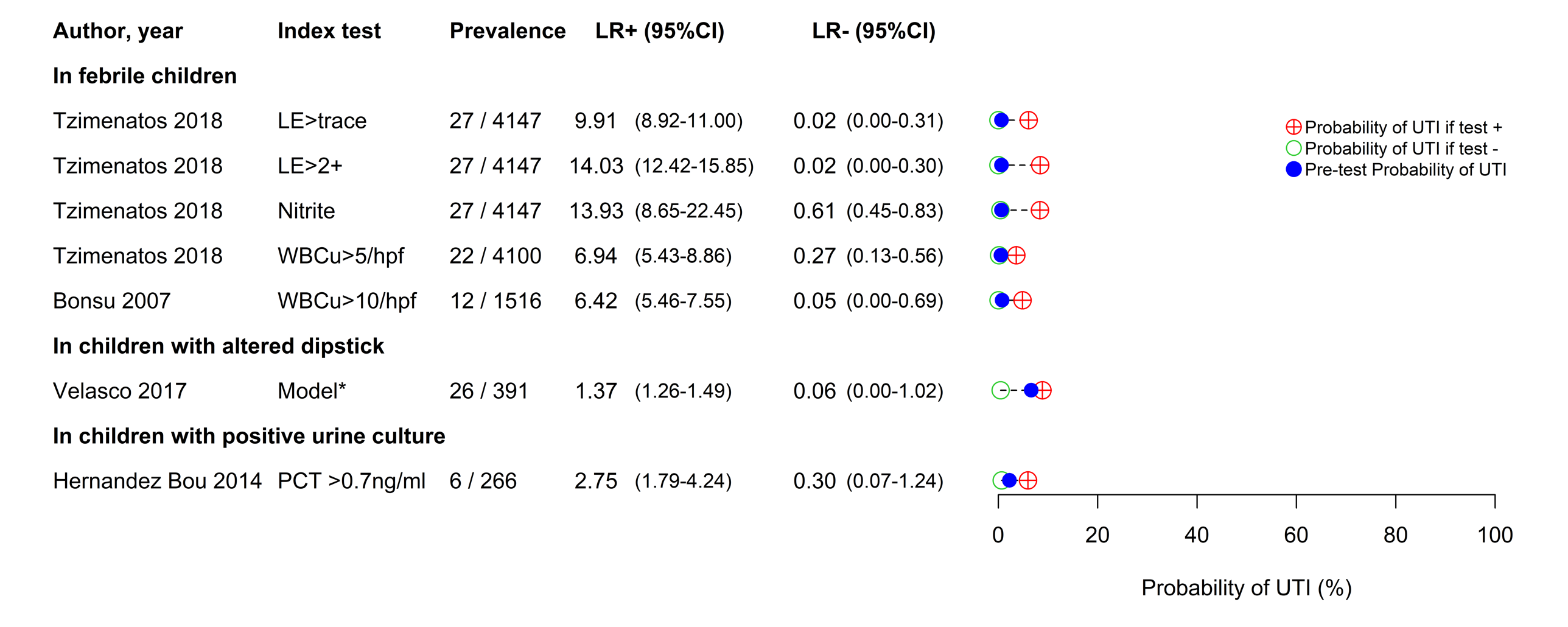
**Prevalence= number of children with uncomplicated UTI /sample size (n), LR+ = positive likelihood ratio, LR- = negative likelihood ratio, 95%CI = 95% confidence intervals, UTI= urinary tract infection, Model* = Not well appearing, <21d, C-reactive protein>20mg/l, PCT>0.5ng/ml (positive if one or more variables present), + = positive, - = negative, WBCu= urine white blood cells, hpf= high power field, LE = leucocyte esterase, PCT= procalcitonin
