# Supplementary material for: Diagnostic value of biomarkers for paediatric urinary tract infections in primary care: systematic review and meta-analysis
Source: BMC Fam Pract. 2021 Sep 27;22:193. doi: 10.1186/s12875-021-01530-9 (PMC8474745; doi:10.1186/s12875-021-01530-9)
Supplement: Supplementary file 6 — Additional file 6: Figures S22–23. Risk of bias and applicability assessment. [file 12875_2021_1530_MOESM6_ESM.docx]

**Additional file 6. (Figures S22-23).** Risk of bias and applicability assessment

**Figure S22 Risk of bias and applicability concerns about each domain for each included study**


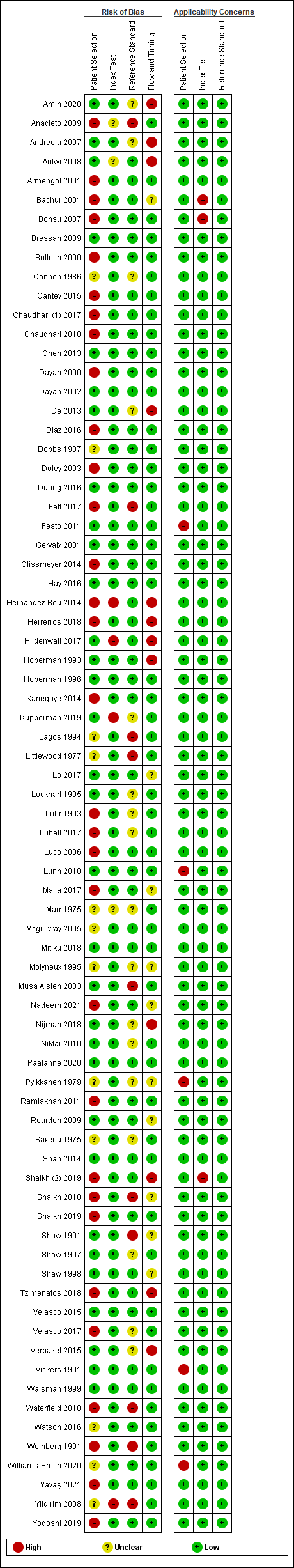


Risk of bias and applicability concerns summary: review authors' judgements about each domain for each included study; assessed by using the QUADAS-2 = Quality assessment of diagnostic accuracy studies

**Figure S23 Risk of bias and applicability concerns as percentages across included studies**


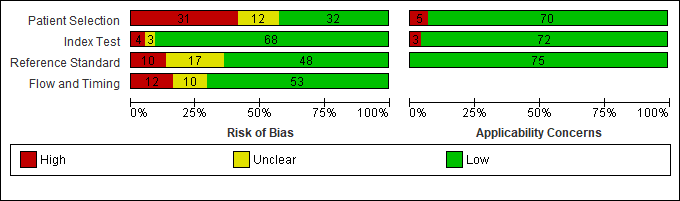


Risk of bias and applicability concerns graph: review authors' judgements about each domain presented as percentages across included studies; assessed by using the QUADAS-2 = Quality assessment of diagnostic accuracy studies
